# Identification of a Ligand Binding Pocket in LdtR from *Liberibacter asiaticus*

**DOI:** 10.3389/fmicb.2015.01314

**Published:** 2015-11-25

**Authors:** Fernando A. Pagliai, Claudio F. Gonzalez, Graciela L. Lorca

**Affiliations:** Department of Microbiology and Cell Science, Genetics Institute and Institute of Food and Agricultural Sciences, University of Florida, GainesvilleFL, USA

**Keywords:** MarR family, benzbromarone, Benz1, bacterial transcription, small molecule

## Abstract

LdtR is a transcriptional activator involved in the regulation of a putative L,D transpeptidase in *Liberibacter asiaticus*, an unculturable pathogen and one of the causative agents of Huanglongbing disease. Using small molecule screens we identified benzbromarone as an inhibitor of LdtR activity, which was confirmed using *in vivo* and *in vitro* assays. Based on these previous results, the objective of this work was to identify the LdtR ligand binding pocket and characterize its interactions with benzbromarone. A structural model of LdtR was constructed and the molecular interactions with the ligand were predicted using the SwissDock interface. Using site-directed mutagenesis, these residues were changed to alanine. Electrophoretic mobility shift assays, thermal denaturation, isothermal titration calorimetry experiments, and *in vivo* assays were used to identify residues T43, L61, and F64 in the Benz1 pocket of LdtR as the amino acids most likely involved in the binding to benzbromarone. These results provide new information on the binding mechanism of LdtR to a modulatory molecule and provide a blue print for the design of therapeutics for other members of the MarR family of transcriptional regulators involved in pathogenicity.

## Introduction

Huanglongbing (HLB) or citrus greening disease has caused devastation to the citrus industry around the world, with no solutions in sight (da Graça, 1991; Bové, 2006; Duan et al., 2009; Gottwald, 2010). Three species of phloem-limited α-proteobacteria have been associated to HLB in different parts of the world: *Liberibacter asiaticus, L. americanus*, and *L. africanus* (Jagoueix et al., 1994; Teixeira et al., 2005). In the USA, *L. asiaticus* has been identified as the causal agent of HLB (Jagoueix et al., 1994). While several efforts had been made to culture this microorganism in laboratory conditions, they have had little success in sustaining growth as axenic cultures (Sechler et al., 2009; Parker et al., 2014). Analyses of the genome sequence revealed that *L. asiaticus* possesses a very simple gene regulatory system, where only 10 transcription factors may control the expression of the entire transcriptome. Based on these observations, we hypothesized that these transcription factors could be used as targets for the development of new therapeutics. LdtR is a member of the MarR family of transcriptional regulators. It acts as a transcriptional activator by binding to the promoter region of the *ldtR* and the downstream gene *ldtP*. Based on sequence identity, *ldtP* was predicted as a L,D-transpeptidase. We found that in the closely related species *Sinorhizobium* *meliloti*, the inactivation of *ldtR* or *ldtP* resulted in morphological changes and reduced tolerance to osmotic stress (Pagliai et al., 2014a). These results indicated that LdtP might play a role in the remodeling of the cell wall in *L. asiaticus*, although it has not been biochemically characterized yet. Based on the reduced survival of the *ldtR* mutant under osmotic stress conditions and the biological relevance of fluctuations in osmolarity in the phloem sap for *L. asiaticus*, LdtR was used as a target for the design of antimicrobials. Using a small molecule screening assay, we identified phloretin, hexestrol, and benzbromarone as inhibitors of LdtR binding to DNA. These observed *in vitro* effects were correlated with a decreased transcriptional activity of *L. asiaticus* in infected citrus shoots after incubation with these chemicals (Pagliai et al., 2014a). Due to the strong effect observed for benzbromarone, it was chosen for further mechanistic studies. Benzbromarone is a non-purine drug that act as a non-competitive analog of the xanthine oxidase. It has been used for more than 30 years in several countries as a therapeutic agent for the treatment of gout, since it reduces the absorption of uric acid in the kidneys (Heel et al., 1977). In humans, benzbromarone is metabolized in the liver by the cytochrome P450 mono-oxygenase and excreted through the bile (Ferber et al., 1981; De Vries et al., 1993).

Members of the MarR family of transcriptional regulators are small proteins that work as sensors of environmental changes (Wilkinson and Grove, 2006), and have been extensively associated with multidrug resistance. However, after years of research it is clear that MarR homologs regulate a variety of metabolic pathways and they are not restricted to the initial assigned role (Mongkolsuk et al., 1998; Providenti and Wyndham, 2001; Stapleton et al., 2002; Wilkinson and Grove, 2004; Hommais et al., 2008; Haque et al., 2009; Davis et al., 2013).

The majority of the reported MarR homologs, negatively regulate transcription by binding as dimers to a DNA sequence located in the promoter region (Perera and Grove, 2010; Grove, 2013). Upon binding of a signal molecule in the dimer interface, these transcription factors lose DNA binding capabilities, allowing transcription to occur. In contrast, only few reports have described MarR family members acting as transcriptional activators (Baumgarth et al., 2005; Manna and Cheung, 2006; Di Fiore et al., 2009; Ludwig et al., 2014; Pagliai et al., 2014b). These include ChlR, a transcription activator that utilizes an oxygen-labile [4Fe–4S] cluster to sense oxygen in *Synechococcus* sp. PCC 7002 (Ludwig et al., 2014); BdlR, a transcriptional activator involved in the detoxification of aromatic compounds in the archaea *Sulfolobus solfataricus* (Di Fiore et al., 2009); and SarR, a transcriptional activator involved in the up-regulation of virulence genes in *Staphylococcus aureus* (Manna and Cheung, 2006). While the roles of these regulators have been addressed, very little is known about the ligand binding pockets or the signal molecules that modulate their activity.

In contrast, several MarR repressors have been described at the molecular level, in presence and absence of inducer molecules (Evans et al., 2001; Wilkinson and Grove, 2006; Saridakis et al., 2008; Kumarevel et al., 2009; Perera et al., 2009; Pagliai et al., 2014b). The structure of MTH313 from *Methanothermobacter thermautotrophicus* was elucidated in presence of salicylate in the dimerization interface, in two asymmetrical pockets. The first site, named SAL1, is located between the dimer interface and the DNA binding domain, while SAL2 was found asymmetrically located 5 Å away from the corresponding symmetrical site (Saridakis et al., 2008). In the crystal structure of ST1710 from *Sulfolobus tokodaii*, salicylate was found only in the corresponding SAL1 pocket (Kumarevel et al., 2009). Based on the location of the SAL2 pocket in MTH313, and the findings of a unique salicylate molecule in ST1710, the biological relevance of the SAL2 site remains unknown in those proteins.

Here, we directed our research to the identification of the binding site of benzbromarone in LdtR, as a step in the understanding of the mechanism of ligand sensing by a MarR protein that acts as a transcriptional activator. Since currently there are not any available structures for MarR members that have been biochemically or genetically characterized as transcriptional activators, we used an unbiased *in silico* modeling to determine the binding site of benzbromarone. Using site-directed mutagenesis we characterized the ligand binding pocket of LdtR. The decreased ligand binding affinities observed for some LdtR mutants *in vitro* was confirmed using *in vivo* experiments in model strains. The results obtained contribute to the understanding of the protein–ligand interaction in the MarR family of transcriptional activators, and provides foundations for the designs of new therapeutics against HLB.

## Materials and Methods

### Bacterial Strains

*Escherichia coli* DH5α cells were used to carry and propagate all vectors. Cultures were grown in Luria-Bertani medium (LB, Difco) at 37°C. *Bacillus subtilis* strains were grown in LB medium at 37°C. *S. meliloti* strains were grown at 30°C in either LB medium or M9 minimal medium with glucose. All strains were grown under aerobic conditions.

When necessary, media was supplemented with ampicillin (100 μg/ml) for *E. coli*; neomycin (100 μg/ml), gentamicin (30 μg/ml), and streptomycin (500 μg/ml) for *S. meliloti*; or with erythromycin (1 μg/ml) for *B. subtilis*. All antibiotics and chemicals were purchased from Sigma-Aldrich (St. Louis, MO, USA). The strains used for this study are listed in **Table 1**.

**Table 1:** Strains and plasmid used in this study.

Name	Relevant genotype	Origin/reference
**Bacterial strains**		
*Escherichia coli* DH5α	φ80 d*lacZ*ΔM15Δ(*lacZYA-argF*)U169 *recA1 endA1 hsdR17* (rk^-^. mk^+^) *supE44 thi-1 gyrA relA1*	Laboratory stock
*E. coli* BL21 (DE3)	*F – ompT gal dcm lon hsdSB*(*rB- mB-*) *aaa*(*DE3* [*lacI lacUV5-T7 gene 1 ind1 sam7 nin5]*)	Life Technologies
*Bacillus subtilis* 168	*trpC2*	*Bacillus* Genetic Stock Center
*Sinorhizobium meliloti* 1021	*expR102*::IS*Rm*2011-1 *expR*. Sm^r^	Galibert et al., 2001
BS6	168 *ΔthrC*::[*P_ldtR_-ldtR-P_ldtP_* (-395 to +792)*-lacZ*]. Em^r^	Pagliai et al., 2014a
BS6A	BS6 *ldtR*(T43A). Em^r^	This work
BS6B	BS6 *ldtR*(L61A). Em^r^	This work
BS6C	BS6 *ldtR*(F64A). Em^r^	This work
SMP2	*S. meliloti* 1021 *ldtR_SMc_* (+29)::*uidA*. Sm^r^. Neo^r^	Pagliai et al., 2014a
SMP2A	*S. meliloti* SMP2 pBBR1MCS-5. Sm^r^. Neo^r^. Gm^r^	Pagliai et al., 2014a
SMP2B	*S. meliloti* SMP2 pSMP4. Sm^r^. Neo^r^. Gm^r^	Pagliai et al., 2014a
SMP4A	*S. meliloti* SMP2 pSMP4 (T43A). Sm^r^. Neo^r^. Gm^r^	This work
SMP4B	*S. meliloti* SMP2 pSMP4 (L61A). Sm^r^. Neo^r^. Gm^r^	This work
SMP4C	*S. meliloti* SMP2 pSMP4 (F64A). Sm^r^. Neo^r^. Gm^r^	This work
SMP5	1021 [*pBBR1MCS-5*]. Sm^r^. Gm^r^	This work
**Plasmids**		
p15TV-L	Expression vector for purification of proteins by nickel affinity chromatography. Ap^r^	Pagliai et al., 2010
pDG1663	*B. subtilis* vector for ectopic integration into *thrC* site containing *E. coli spoVG-lacZ*. Ap^r^, Em^r^	Guérout-Fleury et al., 1996
pRK600	Helper plasmid for triparental mating. pRK2013 Nm::Tn9. Cm^r^	Oke and Long, 1999
pBS6	*P_ldtR_-ldtR-P_ldtP_-lacZ* transcriptional fusion carrying *Liberibacter asiaticus s*equence from -395 to +792 in pDG1663. Ap^r^, Em^r^	Pagliai et al., 2014a
pBS6A	pBS6 (T43A). Ap^r^, Em^r^	This work
pBS6B	pBS6 (L61A). Ap^r^, Em^r^	This work
pBS6C	pBS6 (F64A). Ap^r^, Em^r^	This work
pSMP2	407 bp of *PldtR_SMc_ and ldtR_SMc_* (from -378 to +29) in pVMG. Neo^r^	Pagliai et al., 2014a
pBBR1MCS-5	Broad host range vector. Gm^r^	Kovach et al., 1995
pSMP4	*ldtR* (from +1 to +516) cloned into pBBR1MCS-5 (EcoRI/BamHI). Gm^r^	Pagliai et al., 2014a
pSMP4A	pSMP4 (T43A). Gm^r^	This work
pSMP4B	pSMP4 (L61A). Gm^r^	This work
pSMP4C	pSMP4 (F64A). Gm^r^	This work

### DNA Manipulations and Gene Cloning

Standard methods were used for chromosomal DNA isolation, restriction enzyme digestion, ligation, transformation, and agarose gel electrophoresis (Sambrook et al., 1989). Plasmids were isolated using QIAprep Spin Miniprep Kit (Qiagen, Valencia, CA, USA) and PCR products were purified using QIAquick purification Kit (Qiagen).

Site-directed mutagenesis was performed using the QuikChange Site-directed Mutagenesis kit (Agilent Technologies, Santa Clara, CA, USA) according to the manufacturer’s protocol. The plasmids p15TV-LdtR, pBS6 (Pagliai et al., 2014a), and pBBR1MCS5-LdtR were used as the templates. All selected amino acids were changed to alanine. Mutations were verified by DNA sequencing using T7 and M13 primers. The new generated strains and primers used are described in detail in **Tables 1** and **2**, respectively.

**Table 2:** Oligonucleotides used in this work.

Primer	Oligonucleotide sequence (5′→3′)
**Site-directed mutagenesis**	
LdtR_V26A_Fw	gatatctctggtctatatgcggaatgcttgcgtttggtt
LdtR_V26A_Rv	aaccaaacgcaagcattccgcatatagaccagagatatc
LdtR_R30A_Fw	ctatatgtggaatgcttggctttggttgagcgattacac
LdtR_R30A_Rv	gtgtaatcgctcaaccaaagccaagcattccacatatag
LdtR_E33A_Fw	ggaatgcttgcgtttggttgcgcgattacacagaagtcttttgg
LdtR_E33A_Rv	ccaaaagacttctgtgtaatcgcgcaaccaaacgcaagcattcc
LdtR_R34A_Fw	ggaatgcttgcgtttggttgaggcattacacagaagtcttttgg
LdtR_R34A_Rv	ccaaaagacttctgtgtaatgcctcaaccaaacgcaagcattcc
LdtR_R37A_Fw	cgtttggttgagcgattacacgcaagtcttttggatgttacaagg
LdtR_R37A_Rv	ccttgtaacatccaaaagacttgcgtgtaatcgctcaaccaaacg
LdtR_T43A_Fw	agaagtcttttggatgttgcaagggatgagtttgaaaga
LdtR_T43A_Rv	tctttcaaactcatcccttgcaacatccaaaagacttct
LdtR_L61A_Fw	gtgaatgctgtgcaagcagctttacttttcaatataggtg
LdtR_L61A_Rv	cacctatattgaaaagtaaagctgcttgcacagcattcac
LdtR_F64A_Fw	gctgtgcaagcacttttacttgccaatataggtgatcttgagttaacagc
LdtR_F64A_Rv	gctgttaactcaagatcacctatattggcaagtaaaagtgcttgcacagc
LdtR_N65A_Fw	gctgtgcaagcacttttacttttcgctataggtgatcttgagttaacagc
LdtR_N65A_Rv	gctgttaactcaagatcacctatagcgaaaagtaaaagtgcttgcacagc
LdtR_Y81A_Fw	ggagaattacgttcaagaggagcttatttgggttctaatgtatc
LdtR_Y81A_Rv	gatacattagaacccaaataagctcctcttgaacgtaattctcc
LdtR_Y82A_Fw	ggagaattacgttcaagaggatatgctttgggttctaatgtatc
LdtR_Y82A_Rv	gatacattagaacccaaagcatatcctcttgaacgtaattctcc
LdtR_Y131A_Fw	gagactatttctcaactcgctcaacgtcatatagagtcg
LdtR_Y131A_Rv	cgactctatatgacgttgagcgagttgagaaatagtctc
**Sequencing**	
M13_Fw	gttgtaaaacgacggccagt
M13_Rv	aggaaacagctatgaccatg
T7	taatacgactcactataggg
T7 term	gctagttattgctcagcgg

### Protein Purification

Protein expression and purification was performed as described previously (Pagliai et al., 2014a). Briefly, the His-tagged fusion proteins were overexpressed in *E. coli* BL21-Star(DE3) cells (Life Technologies, Grand Island, NY, USA). The cells were grown with shaking in LB broth at 37°C. Protein expression was induced with 0.5 mM IPTG at OD600 = 0.5. After addition of IPTG, the cells were incubated with shaking at 17°C overnight. The cells were recovered by centrifugation and the pellet resuspended in binding buffer (500 mM NaCl, 5% glycerol, 50 mM Tris pH 8.0, 5 mM imidazole, and 0.5 mM TCEP), and stored at -80°C. The thawed cells were lysed using a French Press, and the lysate centrifuged at 15,000 rpm for 30 min at 4°C. The cell free extract was applied to a metal chelate affinity-column charged with Ni^2+^ (Qiagen). After the column was washed with binding buffer supplemented with 25 mM imidazole, the protein was eluted using elution buffer (binding buffer with 250 mM imidazole). The six-histidine tag was removed from the eluted proteins by treatment with a recombinant His-tagged TEV protease. The cleaved protein was separated from the hexa-histidine tag and the TEV protease by passing the samples through a second column charged with Ni^2+^. Finally, the eluted cleaved proteins were dialyzed against 500 mM NaCl, 5% glycerol, 50 mM Tris pH 8.0, and 0.5 mM TCEP. After dialysis the proteins were aliquoted and stored at -80°C. Protein concentration was determined using the Bio-Rad protein assay (Bio-Rad, Hercules, CA, USA).

### Electrophoresis Mobility Shift Assays (EMSAs)

Gel shift assays of LdtR wild type (WT) and LdtR mutants over DNA containing *P_ldtP_* were conducted using proteins purified and stored according to the procedures described above. Fragments of the *ldtP* promoter were generated by PCR as described previously (Pagliai et al., 2014a). The reaction mix for electrophoresis mobility shift assay (EMSA) contained 1 ng of 5′-Biotin labeled DNA probe, 50 mM Tris-HCl pH 7.2, 150 mM KCl, 10 mg MgCl_2_, 0.01% Triton X100, 12.5 ng/μl Poly(dI-dC) and Poly(dA-dT) non-specific competitor DNAs, purified WT or mutant LdtR (0–800 nM), and benzbromarone (0–1 mM), when indicated. After incubating the mix for 20 min at 37°C, the samples were electrophoresed at 4°C on a 6% acrylamide/bisacrylamide non-denaturing gels, in 0.5X Tris-borate EDTA buffer (TBE) pH 8.3. Then, the DNA was transferred from the polyacrylamide gel to a Hybond-N^+^ membrane (GE Healthcare, Pittsburgh, PA, USA) by electroblotting at 250 mA for 45 min in a semidry transfer (Fisher Scientific, Pittsburgh, PA, USA). The transferred DNA was UV-crosslinked and the biotin labeled DNA was detected using the Phototope-Star Detection Kit (New England Biolabs, Ipswich, MA, USA). Membranes were exposed to Kodak X-ray films. Benzbromarone was prepared as a 100 mM stock solution in 100% DMSO. It was further diluted in LdtR’s dialysis buffer for EMSAs. Vehicle controls were included in all the assays.

### Size-exclusion Chromatography

Protein samples of 100 μL contained 10 μM WT or LdtR mutants, 50 mM Tris pH 8.0, 500 mM NaCl, and 5% glycerol. The sample was incubated 20 min on ice and then injected onto a prepacked Superose 12 10/300 GL gel filtration column (GE Healthcare), connected to a LCC-501 plus (GE Healthcare), and equilibrated with 50 mM Tris pH 8.0, 500 mM NaCl, and 5% glycerol. Filtration was performed in a flow rate of 0.5 ml/min at 4°C. The eluted protein was checked continuously for absorbance at 280 nm using a UV-M II monitor (GE Healthcare). The void volume of the column was determined using Blue dextran 2000. A combination of protein molecular weight standards, including IgG (150 kDa), BSA (66 kDa), Albumin (45 kDa), Trypsinogen (24 kDa), Cytochrome C (12.4 kDa), and Vitamin B12 (1.36 kDa) was applied to the column under the same conditions. The elution volume and molecular mass of each protein standard was used to elaborate a standard curve for further determination of the molecular weight of LdtR and LdtR mutants in the ligand binding pocket. The theoretical molecular weight of LdtR and the mutants was calculated from the amino acid sequence using the Compute pI/Mw tool at the ExPASy Proteomics Server (http://ca.expasy.org/tools/pi_tool.html).

### Thermal Stability Screening by Differential Scanning Fluorimetry

Wild type LdtR and mutants in the ligand binding pocket were screened against different concentrations of benzbromarone (0–65 μM), as previously described (Vedadi et al., 2006; Pagliai et al., 2014a). Proteins were diluted to a final concentration of 30 μM, using 100 mM Tris pH 8.0 and NaCl 150 mM. SYPRO orange (Life Technologies) was added to a final concentration of 5X. Aliquots of 25 μL of protein solution were placed in triplicates in 96-well plates (Bio-Rad), and heated from 25 to 80°C at the rate of 1°C per min. A multicolor real-time PCR detection system (iCycler iQ, Bio-Rad) was used to monitor the unfolding of LdtR, by measuring the increase in the fluorescence of the SYPRO orange. Fluorescence intensities were plotted against temperature for each sample and the generated curves were fitted using the Bolztmann equation with Origin 9 software (Northampton, MA, USA). The midpoint of each transition was calculated and compared to the calculated midpoint for the reference/control sample.

### Isothermal Titration Calorimetry

Measurements were conducted in a MicroCal ITC200 system (GE Healthcare) at 30°C. WT LdtR mutants in the ligand binding pocket were dialyzed overnight against 500 mM NaCl, 5% glycerol, 50 mM Tris pH 8.0, and 0.5 mM TCEP. A solution of 0.5 mM benzbromarone was directly prepared in dialysis buffer. Each titration consisted in a series of 1 μl injections of the ligand into the protein solution. The mean enthalpies measured from injection of the ligand into the buffer were subtracted from raw titration data prior to analysis. Titration curves were fitted by a non-linear least squares method, using Origin 9 software, to a function for the binding of a ligand to a macromolecule (Wiseman et al., 1989). From the curve thus fitted, the parameters Δ*H* (reaction enthalpy), *K_A_* (binding constant, *K_A_* = 1/*K_D_*), and *N* (reaction stoichiometry) were determined. From the values of *K_A_* and Δ*H*, the changes in free energy (Δ*G*) and entropy (Δ*S*) were calculated with the equation Δ*G =* -RT ln*K_A_* = Δ*H* - TΔ*S*, where R is the universal molar gas constant and T is the absolute temperature.

### Construction of Complemented Strains of *S. meliloti*

Plasmid pBBR1MCS5 or its variants pSMP4, pSMP4A-C, harboring the copy of WT LdtR or with mutations in the ligand binding pocket (**Table 1**), were propagated in DH5α, and mobilized into *S. meliloti* SMP2 strain by triparental mating with the aid of plasmid pRK600 (Finan et al., 1986). Transconjugants were selected on M9 medium supplemented with 0.4% sucrose, 120 μg/ml neomycin, 500 μg/ml streptomycin, and 90 μg/ml gentamicin plates. The presence of the plasmids were confirmed by PCR using universal M13 primers.

### *S. meliloti* Osmotic Stress Assays

For the osmotic stress assays, *S. meliloti* cells were grown in M9-glucose minimal broth, supplemented with increasing concentrations of NaCl (8.5–200 mM) in the presence or absence of 25 μM benzbromarone. The OD600 of cells grown in M9 minimal media was continuously monitored, and the growth rate constant (*k*) was calculated as the slope of a plot containing the log_2_OD600 versus time, during the exponential phase of growth. The mean generation time (*g*) was calculated as 1/*k*. The assays were performed in triplicates.

### Construction of *lacZ* Fusions in *B. subtilis*

The strains used for the β-galactosidase assays were constructed as described in Pagliai et al. (2014a) and are listed in **Table 1**.

### β-Galactosidase Assays

The β-galactosidase assays were conducted in *B. subtilis* cells grown at 37°C in LB medium, in absence or presence of different concentrations of benzbromarone (0.1–2.5 μM). After the cultures reached an OD600 = 0.3 (mid-exponential phase), the cells were collected and washed twice with 0.9% NaCl, and then permeabilized with 1% toluene in Z-buffer (60 mM Na_2_HPO_4_, 40 mM NaH_2_PO_4_, 10 mM KCl, 1 mM MgSO_4_, 50 mM β-mercaptoethanol; Miller, 1972). β-galactosidase activity was assayed by following the hydrolysis of chlorophenol red-β-D-galactopyranoside (Sigma-Aldrich). The absorbance at 570 nm was read continuously using a Synergy HT 96-well plate reader (BioTek, Winooski, VT, USA). β-galactosidase activity, expressed as arbitrary units (AUs), was calculated using the slope of absorbance curve normalized with the initial cell density (OD600). The assays were performed in triplicates.

### Statistical Analyses

The statistical significance of the β-galactosidase activities was determined using a Student’s *t*-test.

## Results

### Model of Benzbromarone Binding in LdtR

LdtR was modeled *in silico* using the automated mode of the SWISS-MODEL server (Arnold et al., 2006), and PHYRE2 server (Kelley and Sternberg, 2009). The crystal structure of SP03579 from *Ruegeria pomeroyi* DSS-3 (PDB# 3BJ6) was retrieved as the best template, despite sharing low sequence identity (21%). Since LdtR, as well as other members of the MarR family are commonly found as homodimers in solution (Perera and Grove, 2010), a model of the dimer was generated using 3BJ6 as a template (see Supplementary Data Sheet 2).

**FIGURE 1:**
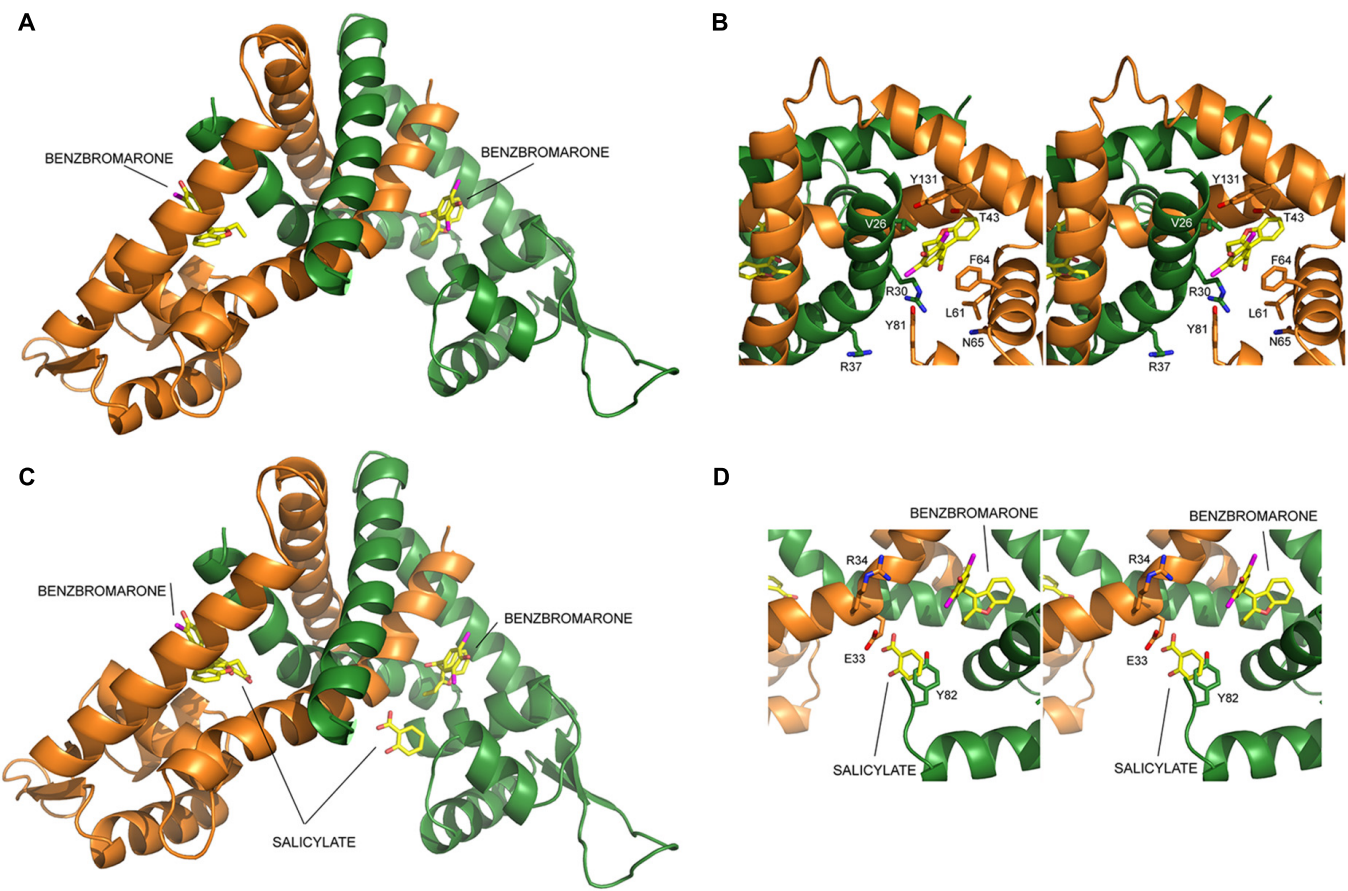
**LdtR model and identification of critical amino acids involved in ligand interaction.** Structural-based identification of residues involved in ligand binding in LdtR. The protein is shown in cartoon representation, with each subunit in a different color (monomer A, orange; monomer B, green), whereas ligands and selected amino acid side chains are shown in sticks representation and labeled, coloring O, N, and Br atoms in red, blue and magenta, whereas the C atoms are shown in yellow for the ligands and in the color of the corresponding subunit for amino acid side-chains. **(A)** Model of the LdtR dimer, with benzbromarone docked in both subunits, suggesting the presence of one ligand binding pocket per subunit. **(B)** Close view of the predicted Benz1 pocket oriented to show the amino acid residues that would be within 4 Å from the docked ligand. **(C)** Ribbon representation of the modeled LdtR dimer superimposing on it the salicylate molecules found in the SAL1 and SAL2 sites of the homologous protein MTH313 of *Methanothermobacter thermautotrophicus* (PBD# 3BPX, Saridakis et al., 2008). **(D)** Close view of the Benz2 pocket, formed by the amino acid residues within a distance of 4 Å of the LdtR SAL2-equivalent pocket.

The molecular interactions between the LdtR dimer and benzbromarone were predicted using the SwissDock web server (Grosdidier et al., 2011), and the DockingServer web interface (Hazai et al., 2009). The molecule of benzbromarone was docked in a variety of orientations within one pocket symmetrically located on both dimer interfaces (**Figure 1A**). One molecule of benzbromarone docked with a predicted Gibbs free energy (Δ*G*) of -8.97 kcal/mol, and it was located between helices Aα1-Aα2-Aα5, and Bα1 (A, chain A; B, chain B, **Figure 1A**). The other molecule docked with a predicted Δ*G* of -7.49 kcal/mol between helices Bα1-Bα2-Bα5, and Aα1 (**Figure 1A**).

In both cases, benzbromarone interacts with residues V26, R30, R37, T43, L61, F64, N65, Y81, and Y131 (**Figure 1B**). In the most favorable model, L61 interacts with benzbromarone carbon atoms C9 and C10 via hydrophobic interactions. Similarly, F64 was predicted to establish hydrophobic interactions with atoms C13-C14-C15-C16 of benzbromarone. T43 was found to establish polar interactions with atom O3. Residue N65 was predicted to establish a halogen bond with Br1, although this interaction was predicted to be less favorable. The type of interaction between other residues of the pocket with benzbromarone could not be predicted.

The docked model of LdtR with benzbromarone was then compared to the structure of MTH313 with salicylate (PDB# 3BPX; Saridakis et al., 2008). We found that the SAL1 pocket in MTH313 aligned with the location where benzbromarone docked with a Δ*G* of -7.49 kcal/mol. This SAL1 equivalent pocket in LdtR was named Benz1 (**Figures 1B,C**). SAL2 did not align with any of the predicted pockets in LdtR, due to the asymmetric location of salicylate in the crystal structure of MTH313. The structural alignment between LdtR and MTH313 also served to identify the equivalent residues for SAL2 in LdtR. The residues E33, R34, and Y82 in LdtR were identified within a distance of 4 Å of the salicylate molecule (**Figure 1D**). This pocket in LdtR was named Benz2.

To determine which of the amino acids in the predicted Benz1 and Benz2 pockets in LdtR are biochemically and biologically relevant, site-directed mutagenesis (to alanine) was conducted on all of them. The mutant proteins were purified and analyzed by size exclusion chromatography, and confirmed that the mutations did not affect dimer formation in solution (data not shown).

### Mutations in LdtR Reduce Binding to *P_ldtP_*

**FIGURE 2:**
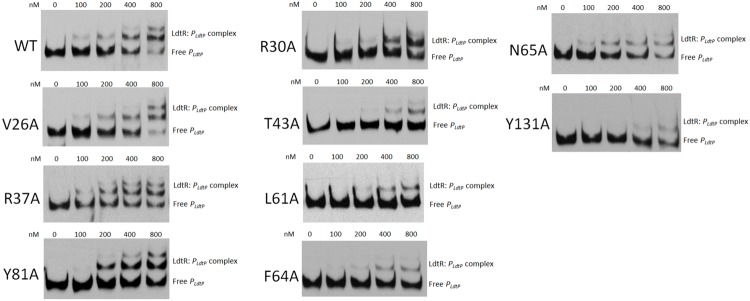
**LdtR mutants in residues R30, T43, L61, F64, N65, and Y131 bind DNA with lower affinity compared to the WT LdtR.** *P_ldtP_* DNA probe was incubated with increasing concentration of WT LdtR or mutants in the Benz1 pocket (V26A, R30A, R37A, T43A, L61A, F64A, N65A, Y81, and Y131A), as indicated on top of each panel. No protein was added to the first lane.

The binding pockets Benz1 and Benz2 are located in the dimerization domains of LdtR. Previous studies of MarR homologs have reported that that mutagenesis in the dimerization interface can directly affect DNA binding activity (Saridakis et al., 2008; Pagliai et al., 2010, 2014b; Gupta and Grove, 2014). Based on these observations, the purified LdtR mutant variants were tested for DNA binding activity on the promoter region of the downstream gene *ldtP* (*P_ldtP_*; Pagliai et al., 2014a). Under the current DNA binding assays conditions, an approximated 50% of binding of LdtR WT to *P_ldtP_* was achieved at 200 nM (**Figure 2**). Under these conditions, only one LdtR:DNA complex was observed. In Benz1 pocket of LdtR, mutants V26A, R37A, and Y81A had similar binding affinities for *P_ldtP_* when compared to the WT LdtR. Mutants R30A, T43A, L61A, F64A, N65A, and Y131A showed decreased affinity for DNA, reaching around 50% of binding to the DNA at approximately 400 nM of protein (**Figure 2**). Conversely, LdtR mutants E33A, R34A, and Y82A, located in the Benz2 pocket, displayed binding affinities for *P_ldtP_* similar to the WT LdtR (Supplementary Figure S1). The decreased binding to DNA observed in some mutants suggest that these residues play a role in the stabilization or flexibility of the dimer form of LdtR (**Figure 1B**).

**FIGURE 3:**
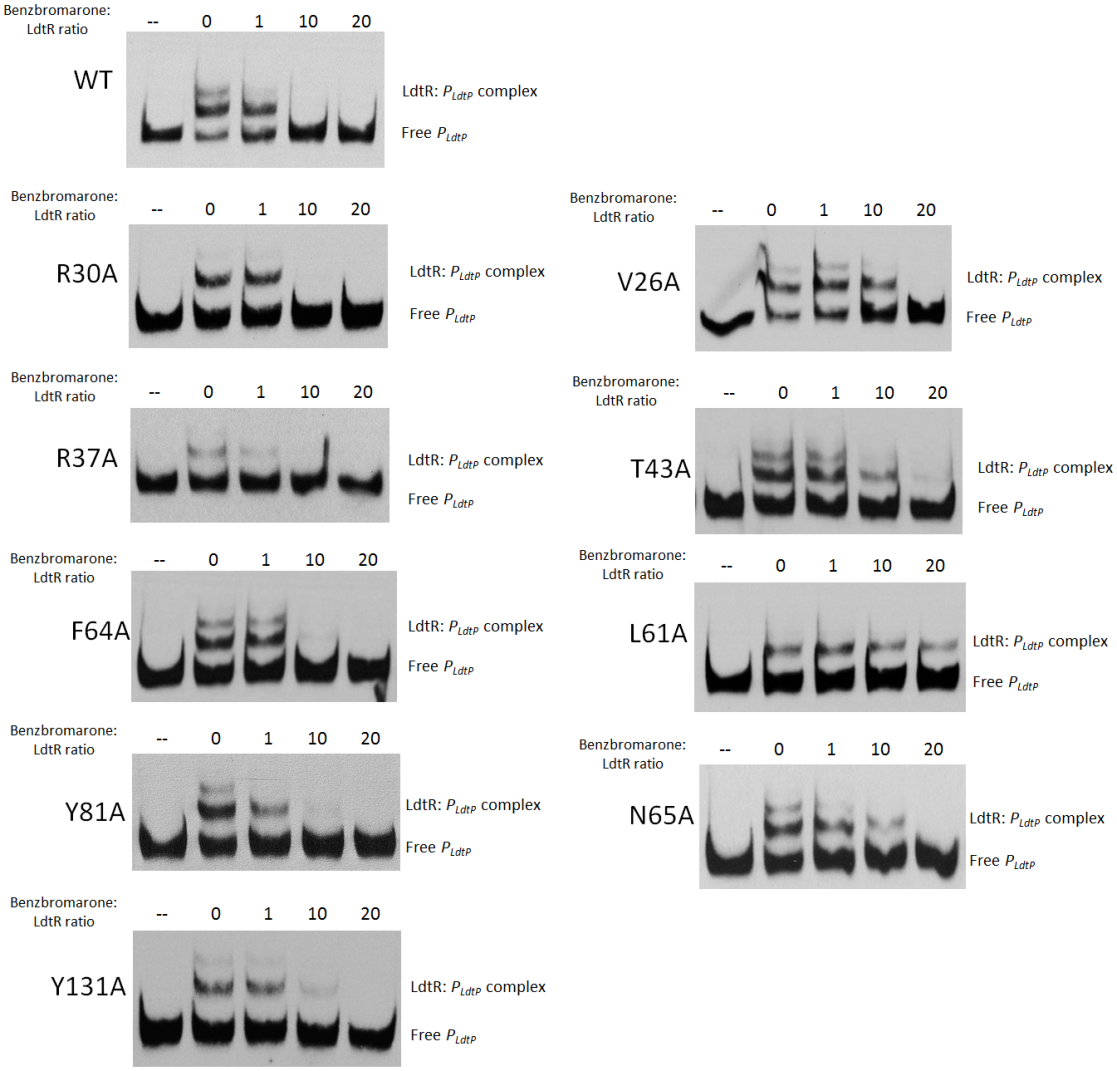
**LdtR residues V26, T43, L61, and N65 may mediate binding to benzbromarone.** *P_ldtP_* DNA probe and WT LdtR or mutants in the Benz1 pocket (V26A, R30A, R37A, T43A, L61A, F64A, N65A, Y81, and Y131A) were incubated with increasing concentrations of benzbromarone. Since some mutant proteins bind DNA with different affinities (**Figure 2**), the benzbromarone:LdtR molar ratio was kept constant, as indicated on top of each panel. No protein was added to the first lane.

The ability of these LdtR mutant proteins to sense and respond to benzbromarone was then tested at the conditions where around 50% of binding to DNA was achieved. In Benz1, the mutant protein in residues R30, R37, F64, Y81, and Y131 interacted with benzbromarone similarly to the WT LdtR, with disruption of the complex occurring at a 10-fold excess of benzbromarone (**Figure 3**). However, LdtR mutants V26A, T43A, and N65A required a 20-fold excess of benzbromarone to disrupt the complex (**Figure 3**). Interestingly, the L61A mutant no longer responded to benzbromarone (**Figure 3**). These results suggest that V26, T43, L61, and N65 might be involved in the interaction of LdtR with benzbromarone *in vitro*.

The LdtR mutants in Benz2, E33A, R34A, and Y82A, bound benzbromarone similarly to the WT LdtR, disrupting the complex formation at similar ratios (data not shown). These results indicate that the residues in the Benz2 pocket may not be relevant for the protein–ligand interactions in LdtR. These results also suggest that benzbromarone binds LdtR in one pocket located in the dimerization interface (Benz1).

### Mutations in Benz1 Affect the Thermal Stability of LdtR

A fluorescence based screening assay was used to assess the thermal stability of mutants in the Benz1 pocket (Pagliai et al., 2010; McFedries et al., 2013). In the absence of benzbromarone the *Tm* of the WT LdtR was 45.1 ± 0.2°C. The mutant Y81A showed a similar *Tm* (44.9 ± 0.12°C) compared to the WT LdtR. The substitution to alanine in residues R37, N65, and Y131 increased their thermal stability (47.8 ± 0.03°C, 46.9 ± 0.14°C, and 46.7 ± 0.25°C, respectively; Supplementary Table S1), while changes in residues V26, R30, T43, and L61 decreased their thermal stability (40.1 ± 0.01°C, 41.3 ± 0.05°C, 42.3 ± 0.07°C, and 42.4 ± 0.13°C, respectively, Supplementary Table S1). These results suggest that these residues may be involved in the stability of the dimer; however, all the mutant proteins behaved as dimers in solution. Additionally, there was no correlation between the potential disturbance in the thermal stability of the dimer and the DNA binding capabilities of the mutants observed in the EMSAs (**Figure 2**).

**FIGURE 4:**
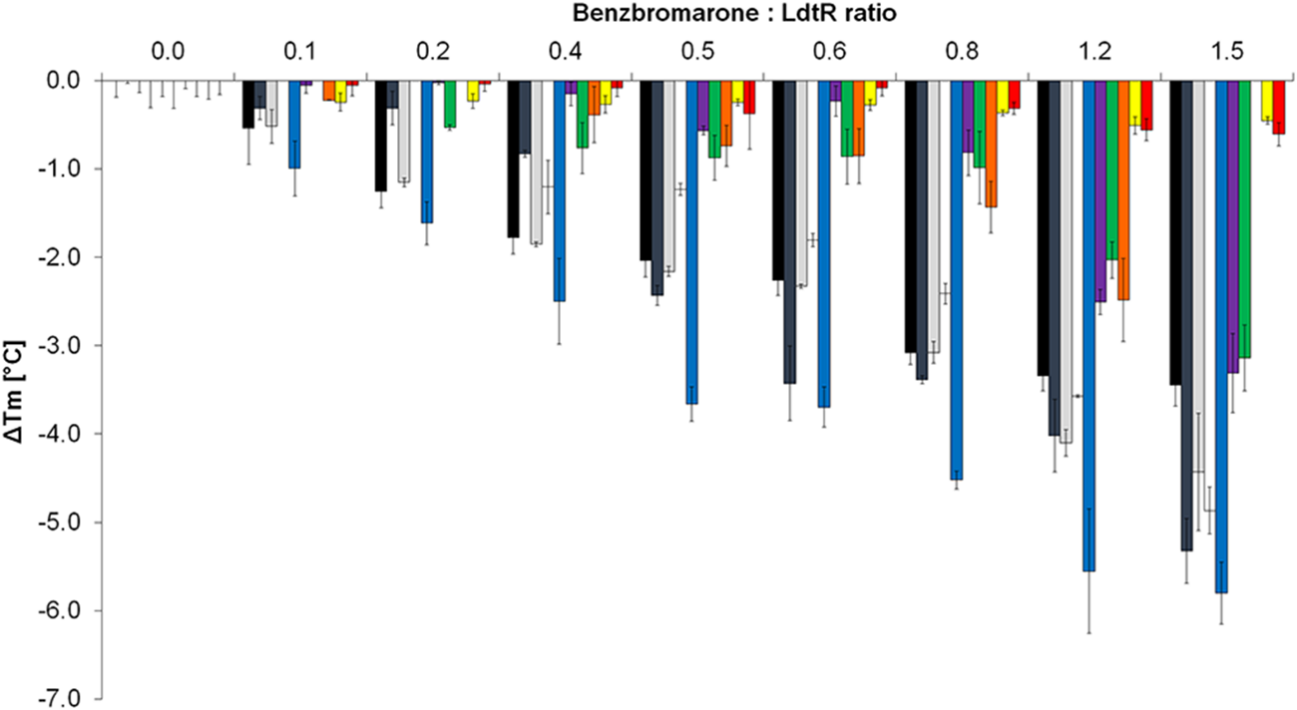
**T43A and L61A mutations increase the thermal stability of LdtR in presence of benzbromarone.** The changes in melting temperatures (Δ*Tm*) of WT LdtR (black bars) and each mutant in the Benz1 pocket were calculated at increasing benzbromarone:protein ratios. The changes in melting temperatures (Δ*Tm*) of LdtR mutants are depicted in different colors (V26A: dark gray; R30A: light gray; R37A: white; Y131A: blue; N65A: purple; Y81A: green; F64A: orange; T43A: yellow; and L61A: red).

The melting temperature (Δ*Tm*) was measured for each LdtR mutant at increasing ratios of benzbromarone and protein (ranging from 0:1 to 1.5:1). It was found that benzbromarone decreased the stability of WT LdtR in a concentration dependent manner (**Figure 4**). At low benzbromarone:LdtR molar ratios (i.e., 0.4:1), the melting temperature of LdtR decreased around 2°C (Δ*Tm* = -1.8 ± 0.2°C), while at a higher ligand:protein molar ratio (i.e., 1.5:1), the melting temperature decreased 3.6 ± 0.2°C.

A similar decrease in thermal stability was observed with mutants V26A, R30A, and R37A when compared to the WT LdtR in presence of different concentrations of benzbromarone (**Figure 4**). These results confirm previous observations that residues R30 and R37 may not be involved in sensing benzbromarone. Noteworthy, mutations in residues V26 and Y131 caused a higher decrease in the melting temperature of LdtR (Δ*Tm* = -5.3 ± 0.4°C and -5.9 ± 0.3°C, respectively) at a 1.5:1 ratio. These results suggest that the change to alanine at the residues V26 or Y131, leads to a protein that is easily destabilized in the presence of high concentrations of benzbromarone.

Mutations T43A, L61A, and F64A resulted in proteins more stable to increasing temperatures, and were not destabilized by benzbromarone at 0:1 to 1:1 ratios. At higher benzbromarone:protein molar ratios (1.5:1), F64A displayed a small decrease in the melting temperature (Δ*Tm* = -2.5 ± 0.4°C), while T43A and L61A were not affected. An intermediate effect of benzbromarone was observed in mutants N65A or Y81A at high ligand:protein molar ratios (Δ*Tm* = -3.3 ± 0.4°C, Δ*Tm* = -3.1 ± 0.4°C; for N65A and Y81A, respectively, **Figure 4**). Taken together, these results indicate that LdtR might bind benzbromarone through interactions with T43, L61, and F64 residues. Residues N65 and Y81 may be involved in further stabilization of these interactions, as suggested by the initial docking model.

### Mutations in Benz1 Decreases the Affinity of LdtR for Benzbromarone

Isothermal titration calorimetry was used to determine the thermodynamic binding parameters for the interactions between LdtR (WT, T43A, L61A, and F64A) and benzbromarone (**Table 3**). The mutant V26A was also included due to the conflicting results between EMSA and thermal stability assays.

**Table 3:** Thermodynamic parameters for the calorimetric titration of LdtR with benzbromarone.

Protein	*N*	*K_D_* [μM]	*K_D_* wt/*K_D_*	Δ*H* [kcal/mol]	Δ*S* [cal/mol/deg]	Δ*G* [kcal/mol]
Wild type	0.34 ± 0.08	4.0 ± 1.6	1.00	-3.0 ± 1.0	14.9	-7.5
V26A	0.30 ± 0.09	9.3 ± 4.6	0.43	-4.7 ± 1.9	7.47	-7.0
T43A	0.30 ± 0.10	17.5 ± 7.5	0.23	-5.5 ± 0.8	3.56	-6.6
L61A	No binding	No binding	ND	ND	ND	ND
F64A	0.34 ± 0.15	16.5 ± 6.0	0.24	-7.0 ± 3.9	-1.35	-6.6

An exothermal heat exchange was observed in the titration of LdtR with benzbromarone, and the data was fitted using a model of “one set of sites” (Supplementary Figure S2). The dissociation constant (*K_D_*) of WT LdtR for benzbromarone was in the low micromolar range (4.0 ± 1.6 μM), in agreement with the EMSA results (**Figure 2**). The stoichiometry of the reaction was around 0.5 moles of benzbromarone per mole of LdtR monomer, which suggest the chemical only binds to one pocket in the LdtR dimer. The affinities of Benz1 mutants for benzbromarone decreased twofold for V26A, and fourfold for T43A and F64A mutants, while the enthalpic contribution to the binding of benzbromarone to L61A mutant was marginal and no detectable affinities could be determined. The stoichiometry of the reaction for T43A and F64A mutants was close to 0.5 moles of benzbromarone per mole of protein. Altogether, these results confirmed the crucial role of residues L61, T43, and F64 from Benz1 in the interaction with benzbromarone.

### Residues in Benz1 Modulate the Regulatory Activity of LdtR

The ability of LdtR mutants in Benz1 to sense and respond to benzbromarone was tested *in vivo* using a transcriptional fusion to the *lacZ* reporter gene in *B. subtilis* (Pagliai et al., 2014a). Mutations R37A, T43A, L61A, F64A, N65A, or Y81A were introduced in *ldtR* contained in plasmid pBS6 (**Table 1**).

**FIGURE 5:**
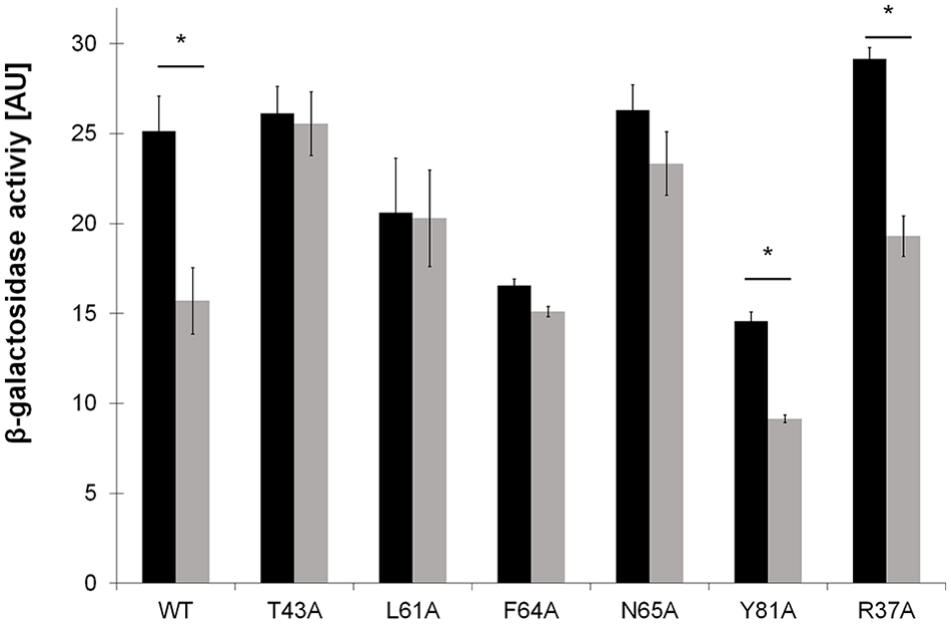
***In vivo* assessment of mutations in Benz1 on the modulation of gene expression by LdtR.** β-galactosidase activities (AU) were determined at mid-exponential phase in *Bacillus subtilis* cells grown in the absence (black bars) or presence (light gray bars) of 1 μM benzbromarone. The experiments were performed in triplicates and the statistical significance (*p* < 0.05) of the β-galactosidase activities in the absence or presence of benzbromarone is depicted with an asterisk.

First, the ability of LdtR containing mutations in Benz1 to induce the expression of the *lacZ* reporter gene was determined. It was found that LdtR mutants in R37A, T43A, and N65A had similar values of β-galactosidase activities compared to the WT LdtR (*p* < 0.05; **Figure 5**). However, mutants L61A, F64A, and Y81A showed a 20, 35, and 45% decrease, respectively, in β-galactosidase activities when compared to the WT LdtR. These results are in agreement with the reduced DNA binding ability of L61A and F64A mutants observed in EMSA.

The modulatory effects of benzbromarone on the trans-criptional activity of LdtR or its mutant variants was then determined by measuring β-galactosidase activity. In *B. subtilis* strain BS6 containing the WT LdtR it was observed that the transcriptional activation of the reporter gene decreased as the concentration of benzbromarone increased in the media (Supplementary Figure S3). 1 μM benzbromarone reduced the β-galactosidase activity by 40%, while concentrations higher than 5 μM were found to be toxic to the cells (data not shown). Similar to the WT LdtR, a significant decrease (around 40%) in the β-galactosidase activity was observed in LdtR mutants Y81A and R37A in presence of 1 μM benzbromarone compared to the control conditions (*p* < 0.05; **Figure 5**). The LdtR mutant N65A showed a non-statistically significant decrease of 12% in the β-galactosidase activity compared to the control cells grown in absence benzbromarone (*p* < 0.05; **Figure 5**). In contrast, mutants T43A, L61A, and F64A, showed similar levels of β-galactosidase activity in presence or absence of benzbromarone (**Figure 5**). These results suggest that residues T43, L61, and F64 may mediate interactions of LdtR with benzbromarone *in vivo*.

### Mutations in Benz1 Decreased the Osmotic Stress Tolerance in *S. meliloti*

As described earlier, the disruption of the *ldtR* homolog gene in *S. meliloti* (strain SMP2) resulted in decreased tolerance to osmotic stress. This phenotype could be rescued by the ectopic expression of WT LdtR (from *L. asiaticus*, strain SMP2B; Pagliai et al., 2014a). To determine the effect of mutations in Benz1 on the tolerance to osmotic stress, we used strain SMP2 (*ldtR*_Smc_ mutant) as the recipient strain. The SMP2 strain was transformed with the empty pBBR1MCS-5 vector as a control (strain SMP2A), WT LdtR (strain SMP2B), or the Benz1 mutants T43A, L61A, or F64A (strains SMP4A, SMP4B, or SMP4C, respectively; **Table 1**). The tolerance to osmotic stress was assessed by determining the growth rate constant (*k*) and the mean generation time (*g*). To this end, each *S. meliloti* strain was grown in liquid cultures with increasing concentrations of sodium chloride, in presence or absence of 25 μM benzbromarone (**Table 4**).

**Table 4:** Mutations in LdtR Benz1 pocket reduce osmotic stress tolerance in *S. meliloti*.

*S. meliloti* strain	Relevant genotype	Growth rate constant, *k* (generations/h)^1^	Mean generation time (h)^2^	Growth rate constant, *k* (generations/h)	Mean generation time (h)
		**No benzbromarone**		**25 μM benzbromarone**	
SMP5	1021-pBBR1MCS-5	0.061 ± 0.003	16.4	0.044 ± 0.003	22.7
SMP2A	Δ*ldtR*_SMc_-pBBR1MCS-5	0.043 ± 0.003	23.3	0.042 ± 0.002	23.8
SMP2B	Δ*ldtR*_SMc_-pBBR1MCS-5::LdtR	0.056 ± 0.004	17.9	0.045 ± 0.002	22.2
SMP4A	Δ*ldtR*_SMc_-pBBR1MCS-5::LdtR(T43A)	0.057 ± 0.002	17.5	0.056 ± 0.004	17.9
SMP4B	Δ*ldtR*_SMc_-pBBR1MCS-5::LdtR(L61A)	0.057 ± 0.003	17.5	0.056 ± 0.005	17.9
SMP4C	Δ*ldtR*_SMc_-pBBR1MCS-5::LdtR(F64A)	0.059 ± 0.002	16.9	0.058 ± 0.003	17.2

*^1^Growth rate constant (*k*) was calculated from the plot of log_2_OD600 versus time.*

*^2^The mean generation time was calculated as 1/*k*.*

When grown in presence of 50 mM NaCl, the *S. meliloti* WT strain harboring an empty pBBR1MCS-5 plasmid (SMP5) had a duplication time of 16.4 h while in the *ldtR*_Smc_ mutant SMP2A, the duplication time increased by 42% (23.3 h). In contrast, the strain SMP2B (containing the WT LdtR from *L. asiaticus*) showed a 9% increase (17.9 h) in the duplication time when compared to the WT strain (**Table 4**). These results confirmed that LdtR from *L. asiaticus* is able to complement the activity of its homolog in *S. meliloti.* Similarly, strains SMP4A, SMP4B, or SMP4C, complemented with Benz1 mutants, showed a similar effect to the WT LdtR (strain SMP2B) in the duplication time (17.5, 17.5, and 16.9 h, respectively, **Table 4**).

The effect of benzbromarone was determined in each *S. meliloti* strain grown in the presence of 50 mM NaCl (**Table 4**). For strain SMP2A, the absence or presence of benzbromarone did not affect the generation time (23.3 and 23.8 h, respectively). However, the addition of benzbromarone caused strains SMP5 and SMP2B to increase the duplication time by 39 and 25%. Interestingly, in strains SMP4A, SMP4B, or SMP4C, no statistical differences in the duplication time were observed. These results indicate that binding of benzbromarone to WT LdtR may competitively inhibit the binding of an unknown native ligand, decreasing the binding affinity of LdtR to DNA. Consequently, a reduced osmotic stress tolerance is observed. The addition of benzbromarone to mutants in Benz1 (strains SMP4A, SMP4B, or SMP4C) had no effect on tolerance to osmotic stress. Taken together, these results confirmed the role of residues T43, L61, and F64 in mediating interactions with benzbromarone.

## Discussion

Benzbromarone was previously identified as an effector molecule of LdtR activity *in vivo* and *in vitro* (Pagliai et al., 2014a). Although benzbromarone might not be a native ligand for LdtR, previous results indicated that it competes with a yet unknown native ligand to decrease binding of LdtR to its cognate binding site, thus inhibiting transcriptional activation (Pagliai et al., 2014a). In this report, using structural modeling we proposed Benz1 as the benzbromarone binding pocket in LdtR. The role of the residues T43, L61, and F64 in the modulation of transcriptional activity by benzbromarone was evaluated *in vitro*, through EMSA and ITC titrations, and *in vivo* using reporter assays and genetic complementation in model microorganisms.

Despite the large number of MarR homologs described in diverse microbial genomes, the affinities and stoichiometry of effector molecules has only been characterized in a few members. We determined that benzbromarone binds LdtR with an affinity within the low micromolar range (*K_D_* = 4.0 ± 1.6 μM) and a stoichiometry close to 0.5:1. Although the initial docking prediction suggested the presence of two ligand pockets in the LdtR dimer, the ITC data confirmed that only one site in the dimeric LdtR is occupied by benzbromarone. The stoichiometry of the reaction between benzbromarone and LdtR follow the half-of-the-sites reactivity phenomena (Levitzki et al., 1971; Anderson et al., 1999), where the binding of the first molecule of benzbromarone may induced a conformational change in LdtR that precludes the binding of the second molecule in the opposite ligand binding pocket. These results may explain, at the biochemical level, the large structural movement observed in the MTH313 DNA binding lobes upon binding of salicylate in the SAL1 pocket. In absence of salicylate, the DNA binding domains of MTH313 are separated by 14 Å while in presence of salicylate, the DNA binding lobes are separated by 21 Å (Saridakis et al., 2008). This movement would allow the binding or release of the protein from the cognate promoter. Furthermore, the affinity value between benzbromarone and LdtR is similar to those reported for other MarR homologs, including EmrR binding to CCCP (between 2 and 25 μM; Brooun et al., 1999; Xiong et al., 2000), and ST1710 binding to ethidium (13.7 μM; Yu et al., 2009). For non-cooperative binders, the reported stoichiometry fall within a narrow range from 2:1 for novobiocin: LVIS553 (Pagliai et al., 2010) to 1:1 for ethidium: ST1710 or CCCP: ST1710 (Yu et al., 2009).

The availability of crystal structures for several members of the MarR family have provided insights into the binding sites of several molecules; however, the functional relevance of these sites has only been addressed in a few cases. In *E. coli*, the structure of MarR showed salicylate bound to sites A and B, both located in the DNA binding domain (Alekshun et al., 2001). In contrast, in MTH313 salicylate was located in the dimer interface in two asymmetric pockets, SAL1 and SAL2 (Saridakis et al., 2008). A recent report showed that mutagenesis of the residues in the SAL-A and SAL-B pockets in *E. coli* did not affect the interactions with salicylate (Duval et al., 2013). However, mutations in residues located in the SAL1 pocket (corresponding to those in MTH313) showed decreased binding to salicylate. Here, we performed a detailed structure based site-directed mutagenesis in LdtR to show that benzbromarone may bind to residues T43, L61, F64 in Benz1 (equivalent to SAL1 pocket), while residues in the predicted SAL2 pocket were not involved in ligand binding. These results are in agreement with our previous findings in *Lactobacillus brevis*, where mutations in the LVIS553 predicted SAL1 pocket impaired binding to its native ligand novobiocin, and mutations in the TstR SAL1 equivalent pocket impaired binding to sulfite (Pagliai et al., 2010, 2014b). Although the decreased interaction between the ligand and the mutants T43A, L61A, and F64A may suggest that these residues are involved in the interaction with benzbromarone, the possibility that the decreased interaction is due to changes in the overall structure of the dimer cannot be ruled out. Altogether, these data indicate that small molecules binding in the SAL1 pocket might be a common regulatory mechanism in the MarR family of regulators.

A structural alignment of LdtR homologs showed that all residues in the Benz1 pocket, with the exception of T43 (often replaced by isoleucine), are conserved in other rhizobiales (Supplementary Figure S4). These observations suggest that residues in Benz1 may also be involved in sensing the natural ligand of LdtR, which acts as an inducer of DNA binding. The conservation of key amino acids in the ligand binding pocket in other causative agents of HLB, such as *L. americanus* and *L. africanus*, provides the foundation for the use of Benz1 as an attractive target for the design of novel and environmentally safer therapeutic agents to treat HLB.

In summary, the identification of the precise location of the ligand pocket aids in the understanding of the mechanism of signal transduction for the MarR family, while the definition of key chemical moieties in the ligand binding pocket will: (i) provide clues into the ligands that are specifically sensed by transcription factors, improving their functional classification; and (ii) aid in the design of new and safer synthetic molecules that specifically target transcription factors, for use as new therapeutics against microorganisms involved in pathogenesis.

## Author Contributions

FP and GL designed, performed, and analyzed the experiments. CG provided assistance and contributed in the analysis and preparation of the manuscript. FP and GL wrote the paper. GL conceived and coordinated the study. All authors reviewed the results and approved the final version of the manuscript.

## Conflict of Interest Statement

The authors declare that the research was conducted in the absence of any commercial or financial relationships that could be construed as a potential conflict of interest.

## References

[B1] Alekshun M. N. Levy S. B. Mealy T. R. Seaton B. A. Head J. F. (2001). The crystal structure of MarR, a regulator of multiple antibiotic resistance, at 2.3 A resolution. *Nat. Struct. Biol.* 8 710–714. 10.1038/90429 11473263

[B2] Anderson A. C. O’Neil R. H. DeLano W. L. Stroud R. M. (1999). The structural mechanism for half-the-sites reactivity in an enzyme, thymidylate synthase, involves a relay of changes between subunits. *Biochemistry* 38 13829–13836. 10.1021/bi991610i 10529228

[B3] Arnold K. Bordoli L. Kopp J. Schwede T. (2006). The SWISS-MODEL workspace: a web-based environment for protein structure homology modelling. *Bioinformatics* 22 195–201. 10.1093/bioinformatics/bti770 16301204

[B4] Baumgarth B. Bartels F. W. Anselmetti D. Becker A. Ros R. (2005). Detailed studies of the binding mechanism of the *Sinorhizobium meliloti* transcriptional activator ExpG to DNA. *Microbiology* 151 259–268. 10.1099/mic.0.27442-0 15632443

[B5] Bové J. M. (2006). Huanglongbing: a destructive, newly-emerging, century-old disease of citrus. *J. Plant Pathol.* 88 7–37. 10.4454/jpp.v88i1.828

[B6] Brooun A. Tomashek J. J. Lewis K. (1999). Purification and ligand binding of EmrR, a regulator of a multidrug transporter. *J. Bacteriol.* 181 5131–5133. 10438794 10.1128/jb.181.16.5131-5133.1999 PMC94011

[B7] da Graça J. V. (1991). Citrus greening disease. *Annu. Rev. Phytopathol.* 29 109–136. 10.1146/annurev.py.29.090191.000545

[B8] Davis J. R. Brown B. L. Page R. Sello J. K. (2013). Study of PcaV from *Streptomyces coelicolor* yields new insights into ligand-responsive MarR family transcription factors. *Nucleic Acids Res.* 41 3888–3900. 10.1093/nar/gkt009 23396446 PMC3616709

[B9] De Vries J. X. Walter-Sack I. Voss A. Forster W. Ilisistegui Pons P. Stoetzer F. (1993). Metabolism of benzbromarone in man: structures of new oxidative metabolites, 6-hydroxy- and 1′-oxo-benzbromarone, and the enantioselective formation and elimination of 1′-hydroxybenzbromarone. *Xenobiotica* 23 1435–1450. 10.3109/00498259309059452 8135044

[B10] Di Fiore A. Fiorentino G. Vitale R. M. Ronca R. Amodeo P. Pedone C. (2009). Structural analysis of BldR from *Sulfolobus solfataricus* provides insights into the molecular basis of transcriptional activation in archaea by marr family proteins. *J. Mol. Biol.* 388 559–569. 10.1016/j.jmb.2009.03.030 19298823

[B11] Duan Y. Zhou L. Hall D. G. Li W. Doddapaneni H. Lin H. (2009). Complete genome sequence of citrus huanglongbing bacterium, “Candidatus *Liberibacter asiaticus*” obtained through metagenomics. *Mol. Plant. Microbe. Interact.* 22 1011–1020. 10.1094/MPMI-22-8-1011 19589076

[B12] Duval V. McMurry L. M. Foster K. Head J. F. Levy S. B. (2013). Mutational analysis of the multiple-antibiotic resistance regulator MarR reveals a ligand binding pocket at the interface between the dimerization and DNA binding domains. *J. Bacteriol.* 195 3341–3351. 10.1128/JB.02224-12 23687277 PMC3719538

[B13] Evans K. Adewoye L. Poole K. (2001). MexR repressor of the mexAB-oprM multidrug eﬄux operon of *Pseudomonas aeruginosa*: identification of MexR binding sites in the mexA-mexR intergenic region. *J. Bacteriol.* 183 807–812. 10.1128/JB.183.3.807-812.2001 11208776 PMC94945

[B14] Ferber H. Vergin H. Hitzenberger G. (1981). Pharmacokinetics and biotransformation of benzbromarone in man. *Eur. J. Clin. Pharmacol.* 19 431–435. 10.1007/BF00548587 7250176

[B15] Finan T. M. Kunkel B. De Vos G. F. Signer E. R. (1986). Second symbiotic megaplasmid in *Rhizobium meliloti* carrying exopolysaccharide and thiamine synthesis genes. *J. Bacteriol.* 167 66–72. 3013840 10.1128/jb.167.1.66-72.1986 PMC212841

[B16] Galibert F. Finan T. M. Long S. R. Puhler A. Abola P. Ampe F. (2001). The composite genome of the legume symbiont *Sinorhizobium meliloti*. *Science* 293 668–672. 10.1126/science.1060966 11474104

[B17] Gottwald T. R. (2010). Current epidemiological understanding of citrus Huanglongbing. *Annu. Rev. Phytopathol.* 48 119–139. 10.1146/annurev-phyto-073009-114418 20415578

[B18] Grosdidier A. Zoete V. Michielin O. (2011). SwissDock, a protein-small molecule docking web service based on EADock DSS. *Nucleic Acids Res.* 39 W270–W277. 10.1093/nar/gkr366 21624888 PMC3125772

[B19] Grove A. (2013). MarR family transcription factors. *Curr. Biol.* 23 R142–R143. 10.1016/j.cub.2013.01.013 23428319

[B20] Guérout-Fleury A. M. Frandsen N. Stragier P. (1996). Plasmids for ectopic integration in *Bacillus subtilis*. *Gene* 180 57–61. 10.1016/S0378-1119(96)00404-0 8973347

[B21] Gupta A. Grove A. (2014). Ligand-binding pocket bridges DNA-binding and dimerization domains of the urate-responsive MarR homologue MftR from *Burkholderia thailandensis*. *Biochemistry* 53 4368–4380. 10.1021/bi500219t 24955985 PMC4100783

[B22] Haque M. M. Kabir M. S. Aini L. Q. Hirata H. Tsuyumu S. (2009). SlyA, a MarR family transcriptional regulator, is essential for virulence in *Dickeya dadantii* 3937. *J. Bacteriol.* 191 5409–5418. 10.1128/JB.00240-09 19542281 PMC2725626

[B23] Hazai E. Kovács S. Demkó L. Bikádi Z. (2009). DockingServer: molecular docking calculations online. *Acta Pharm. Hung.* 79 17–21. 19526678

[B24] Heel R. C. Brogden R. N. Speight T. M. Avery G. S. (1977). Benzbromarone: a review of its pharmacological properties and therapeutic use in gout and hyperuricaemia. *Drugs* 14 349–366. 10.2165/00003495-197714050-00002 338280

[B25] Hommais F. Oger-Desfeux C. Van Gijsegem F. Castang S. Ligori S. Expert D. (2008). PecS is a global regulator of the symptomatic phase in the phytopathogenic bacterium *Erwinia chrysanthemi* 3937. *J. Bacteriol.* 190 7508–7522. 10.1128/JB.00553-08 18790868 PMC2576657

[B26] Jagoueix S. Bove J. M. Garnier M. (1994). The phloem-limited bacterium of greening disease of citrus is a member of the alpha subdivision of the *Proteobacteria*. *Int. J. Syst. Bacteriol.* 44 379–386. 10.1099/00207713-44-3-379 7520729

[B27] Kelley L. A. Sternberg M. J. E. (2009). Protein structure prediction on the Web: a case study using the Phyre server. *Nat. Protoc.* 4 363–371. 10.1038/nprot.2009.2 19247286

[B28] Kovach M. E. Elzer P. H. Hill D. S. Robertson G. T. Farris M. A. Roop R. M. (1995). Four new derivatives of the broad-host-range cloning vector pBBR1MCS, carrying different antibiotic-resistance cassettes. *Gene* 166 175–176. 10.1016/0378-1119(95)00584-1 8529885

[B29] Kumarevel T. Tanaka T. Umehara T. Yokoyama S. (2009). ST1710-DNA complex crystal structure reveals the DNA binding mechanism of the MarR family of regulators. *Nucleic Acids Res.* 37 4723–4735. 10.1093/nar/gkp496 19509310 PMC2724296

[B30] Levitzki A. Stallcup W. B. Koshland D. E. (1971). Half-of-the-sites reactivity and the conformational states of cytidine triphosphate synthetase. *Biochemistry* 10 3371–3378. 10.1021/bi00794a009 4940762

[B31] Ludwig M. Pandelia M. E. Chew C. Y. Zhang B. Golbeck J. H. Krebs C. (2014). ChlR protein of *Synechococcus* sp. PCC 7002 is a transcription activator that uses an oxygen-sensitive [4Fe-4S] cluster to control genes involved in pigment biosynthesis. *J. Biol. Chem.* 289 16624–16639. 10.1074/jbc.M114.561233 24782315 PMC4059106

[B32] Manna A. C. Cheung A. L. (2006). Transcriptional regulation of the agr locus and the identification of DNA binding residues of the global regulatory protein SarR in *Staphylococcus aureus*. *Mol. Microbiol.* 60 1289–1301. 10.1111/j.1365-2958.2006.05171.x 16689803

[B33] McFedries A. Schwaid A. Saghatelian A. (2013). Methods for the elucidation of protein-small molecule interactions. *Chem. Biol.* 20 667–673. 10.1016/j.chembiol.2013.04.008 23706633

[B34] Miller J. H. (1972). *Experiments in Molecular Genetics.* New York, NY: Cold Spring Harbor.

[B35] Mongkolsuk S. Praituan W. Loprasert S. Fuangthong M. Chamnongpol S. (1998). Identification and characterization of a new organic hydroperoxide resistance (ohr) gene with a novel pattern of oxidative stress regulation from *Xanthomonas campestris* pv. *phaseoli*. *J. Bacteriol.* 180 2636–2643. 9573147 10.1128/jb.180.10.2636-2643.1998 PMC107214

[B36] Oke V. Long S. R. (1999). Bacteroid formation in the *Rhizobium*-legume symbiosis. *Curr. Opin. Microbiol.* 2 641–646. 10.1016/S1369-5274(99)00035-1 10607628

[B37] Pagliai F. A. Gardner C. L. Bojilova L. Sarnegrim A. Tamayo C. Potts A. H. (2014a). The transcriptional activator LdtR from “Candidatus *Liberibacter* asiaticus” mediates osmotic stress tolerance. *PLoS Pathog.* 10:e1004101 10.1371/journal.ppat.1004101 PMC3999280 24763829

[B38] Pagliai F. A. Murdoch C. C. Brown S. M. Gonzalez C. F. Lorca G. L. (2014b). A dual role of the transcriptional regulator TstR provides insights into cyanide detoxification in Lactobacillus brevis. *Mol. Microbiol.* 92 853–871. 10.1111/mmi.12598 24684290 PMC4038355

[B39] Pagliai F. A. Gardner C. L. Pande S. G. Lorca G. L. (2010). LVIS553 transcriptional regulator specifically recognizes novobiocin as an effector molecule. *J. Biol. Chem.* 285 16921–16930. 10.1074/jbc.M110.111138 20308066 PMC2878039

[B40] Parker J. K. Wisotsky S. R. Johnson E. G. Hijaz F. M. Killiny N. Hilf M. E. (2014). Viability of “Candidatus *Liberibacter asiaticus*” prolonged by addition of citrus juice to culture medium. *Phytopathology* 104 15–26. 10.1094/PHYTO-05-13-0119-R 23883155

[B41] Perera I. C. Grove A. (2010). Molecular mechanisms of ligand-mediated attenuation of DNA binding by MarR family transcriptional regulators. *J. Mol. Cell Biol.* 2 243–254. 10.1093/jmcb/mjq021 20716550

[B42] Perera I. C. Lee Y. H. Wilkinson S. P. Grove A. (2009). Mechanism for attenuation of DNA binding by MarR family transcriptional regulators by small molecule ligands. *J. Mol. Biol.* 390 1019–1029. 10.1016/j.jmb.2009.06.002 19501097

[B43] Providenti M. A. Wyndham R. C. (2001). Identification and functional characterization of CbaR, a MarR-Like modulator of the cbaABC-encoded chlorobenzoate catabolism pathway. *Appl. Environ. Microbiol.* 67 3530–3541. 10.1128/AEM.67.8.3530-3541.2001 11472929 PMC93053

[B44] Sambrook J. Fritsch E. F. Maniatis T. (1989). *Molecular Cloning: A Laboratory Manual.* New York, NY: Cold Spring Harbor laboratory press.

[B45] Saridakis V. Shahinas D. Xu X. Christendat D. (2008). Structural insight on the mechanism of regulation of the MarR family of proteins: high-resolution crystal structure of a transcriptional repressor from *Methanobacterium* thermoautotrophicum. *J. Mol. Biol.* 377 655–667. 10.1016/j.jmb.2008.01.001 18272181

[B46] Sechler A. Schuenzel E. L. Cooke P. Donnua S. Thaveechai N. Postnikova E. (2009). Cultivation of “Candidatus *Liberibacter asiaticus*”, “ca. *L. africanus*”, *and* “Ca. *L. americanus*” associated with huanglongbing. *Phytopathology* 99 480–486. 10.1094/PHYTO-99-5-0480 19351243

[B47] Stapleton M. R. Norte V. A. Read R. C. Green J. (2002). Interaction of the *Salmonella* typhimurium transcription and virulence factor SlyA with target DNA and identification of members of the SlyA regulon. *J. Biol. Chem.* 277 17630–17637. 10.1074/jbc.M110178200 11882648

[B48] Teixeira D do C. Saillard C. Eveillard S. Danet J. L. da Costa P. I. (2005). “Candidatus *Liberibacter americanus*”, associated with citrus huanglongbing (greening disease) in São Paulo State. Brazil. *Int. J. Syst. Evol. Microbiol.* 55 1857–1862. 10.1099/ijs.0.63677-0 16166678

[B49] Vedadi M. Niesen F. H. Allali-Hassani A. Fedorov O. Y. Finerty P. J. Wasney G. A. (2006). Chemical screening methods to identify ligands that promote protein stability, protein crystallization, and structure determination. *Proc. Natl. Acad. Sci. U.S.A.* 103 15835–15840. 10.1073/pnas.0605224103 17035505 PMC1595307

[B50] Wilkinson S. P. Grove A. (2004). HucR, a novel uric acid-responsive member of the MarR family of transcriptional regulators from *Deinococcus radiodurans*. *J. Biol. Chem.* 279 51442–51450. 10.1074/jbc.M405586200 15448166

[B51] Wilkinson S. P. Grove A. (2006). Ligand-responsive transcriptional regulation by members of the MarR family of winged helix proteins. *Curr. Issues Mol. Biol.* 8 51–62. 16450885

[B52] Wiseman T. Williston S. Brandts J. F. Lin L. N. (1989). Rapid measurement of binding constants and heats of binding using a new titration calorimeter. *Anal. Biochem.* 179 131–137. 10.1016/0003-2697(89)90213-3 2757186

[B53] Xiong A. Gottman A. Park C. Baetens M. Pandza S. Matin A. (2000). The EmrR protein represses the *Escherichia coli* emrRAB multidrug resistance operon by directly binding to its promoter region. *Antimicrob. Agents Chemother.* 44 2905–2907. 10.1128/AAC.44.10.2905-2907.2000 10991887 PMC90178

[B54] Yu L. Fang J. Wei Y. (2009). Characterization of the ligand and DNA binding properties of a putative archaeal regulator ST1710. *Biochemistry* 48 2099–2108. 10.1021/bi801662s 19166356

