# Supplementary material for: Identification of a Ligand Binding Pocket in LdtR from Liberibacter asiaticus
Source: Front Microbiol. 2015 Nov 25;6:1314. doi: 10.3389/fmicb.2015.01314 (PMC4658428; doi:10.3389/fmicb.2015.01314)
Supplement: Supplementary file 1 [file Data_Sheet_1.DOCX]

Supplementary Material

**Identification of the Ligand Binding Pocket in LdtR from *Liberibacter asiaticus***

*Fernando A. Pagliai^1^, Claudio F. Gonzalez^1^, and Graciela L. Lorca^1,*^*

^1^Department of Microbiology and Cell Science, Genetics Institute. Institute of Food and Agricultural Sciences, University of Florida. 2033 Mowry road. PO Box 103610. Gainesville, FL 32610-3610. USA.

**Running Head**: Molecular mechanisms of ligand binding in LdtR

^*^Correspondence:

Dr. Graciela L. Lorca,

Department of Microbiology and Cell Science, Genetics Institute and Institute of Food and Agricultural Sciences, University of Florida,

2033 Mowry Road, Room 307, Genetics Institute 325E, P.O. Box 103610,

Gainesville, FL 32610- 3610, USA

Email: glorca@ufl.edu, Phone: (352) 273-8090


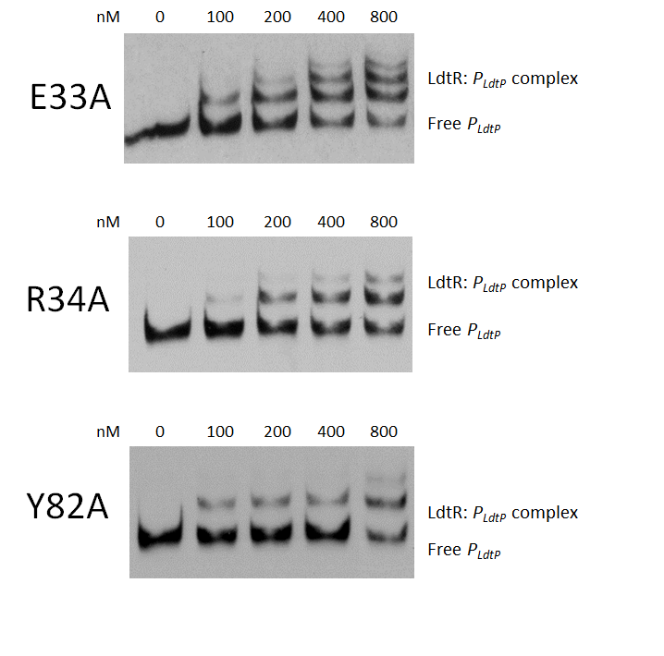


**Supplementary Figure 1.** Mutants in the LdtR Benz2 (SAL2-equivalent pocket) bind DNA similar to the WT LdtR. *P_ldtP_* DNA probe was incubated with increasing concentration of mutants in the Benz2 pocket (E33A, R34A, Y82A). The concentration of each LdtR mutant used in EMSA is indicated on top of each panel. No protein was added to the first lane.


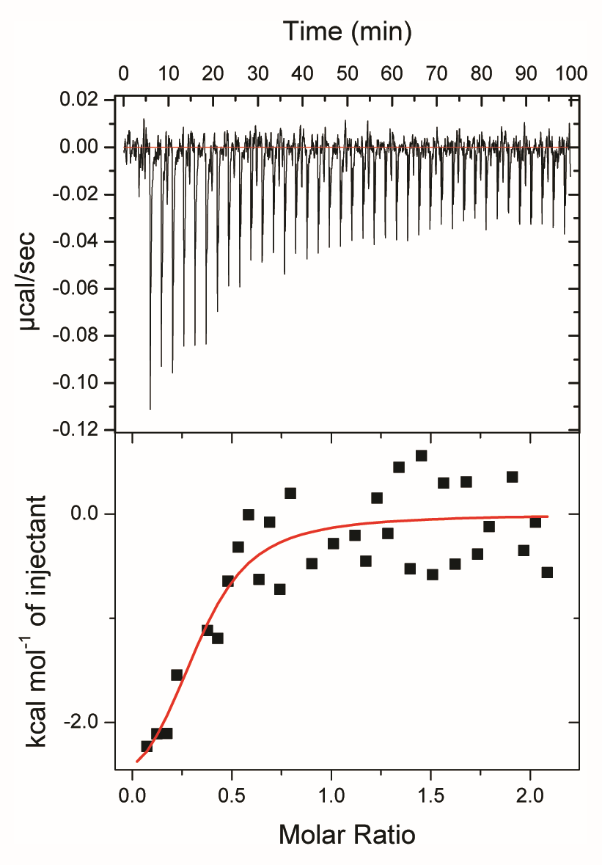


**Supplementary Figure 2.** Isothermal titration calorimetry data for the binding of benzbromarone to WT LdtR. The measurement of the heat changes (upper panel) and the integrated peak areas (lower panel) were conducted in a series of 1 μl injections of 0.5 mM benzbromarone into 50 μM of LdtR. The protein was dialyzed overnight against 50 mM Tris pH 8.0, 500 mM NaCl, 5% glycerol, and 0.5 mM TCEP. Benzbromarone was prepared at a stock of 100 mM in 100 % DMSO and diluted to 0.5 mM in the same dialysis buffer. The experiments were carried out at 30°C in a MicroCal ITC200 system.


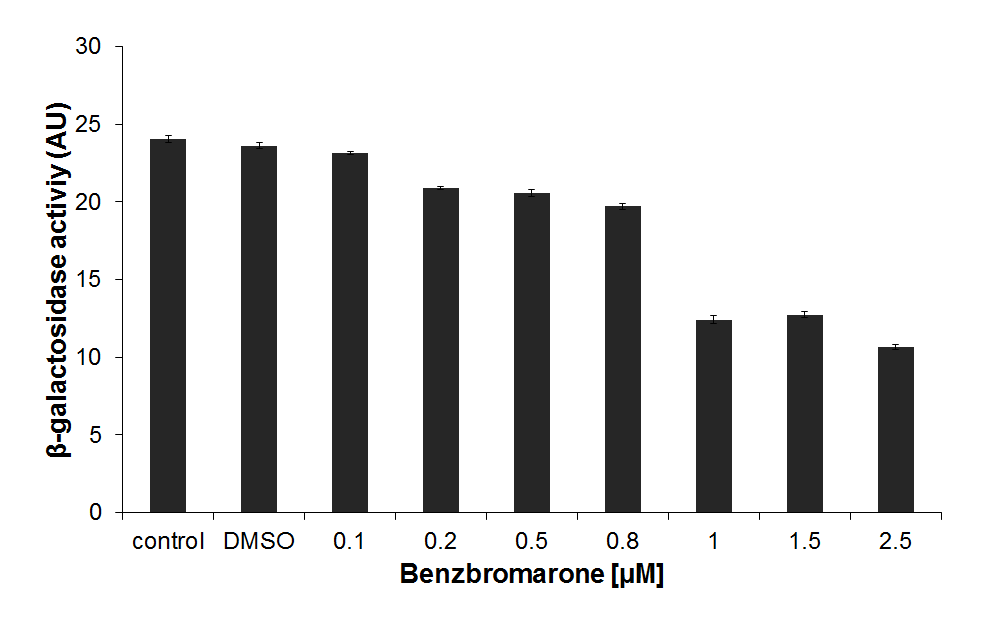


**Supplementary Figure 3.** Benzbromarone decreases the transcriptional activation of the *lacZ* reporter gene in a concentration dependent manner. The effect of different concentration of benzbromarone (0 ─ 2.5 µM) was tested in BS6 strain, carrying WT LdtR (Pagliai et al., 2014).

**
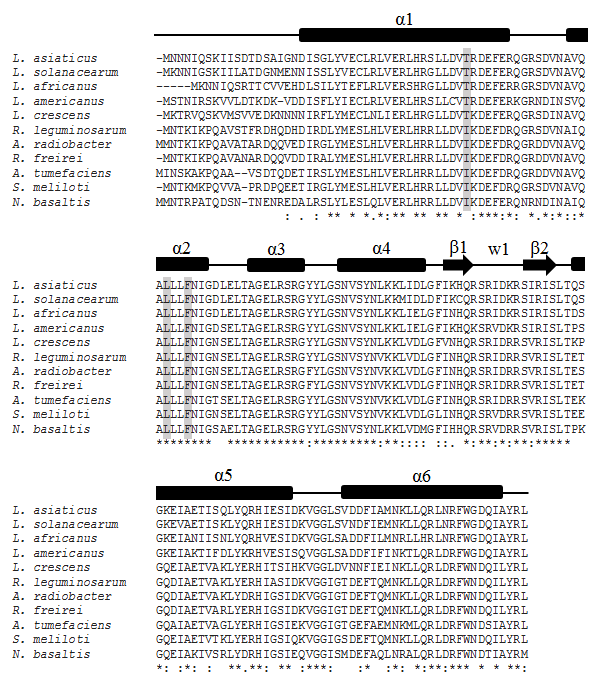
**

**Supplementary Figure 4.** Sequence based alignment of LdtR and its close homologs from the rhizobiales order. The alignment was performed using Clustal Omega (Sievers et al., 2011). The alignment included LdtR homologs from *L. solanacearum* (gi|736420077), *L. africanus* (gi|828173143), *L. americanus* (gi|494821547), *L. crescens* (gi|505086406), *Rhizobium leguminosarum* (gi|653789377), *Agrobacterium radiobacter* (gi|221722952), *R. freirei* (gi|739290927), *A. tumefaciens* (gi|756167895), *S. meliloti* (gi|504339572), and *Nitratireductor basaltis* (gi|738532899). The predicted secondary structure elements are depicted on top of the alignment, α helices as rectangles and β barrels as arrows. The residues from Benz1 that interact with benzbromarone are boxed in a grey rectangle.

**Supplementary Table 1.** Determination of the melting temperature (*Tm*) of WT LdtR and the mutants in the Benz1 pocket.

| **LdtR** | ***Tm* (°C)** |
| --- | --- |
| WT | 45.1 ±0.2 |
| V26A | 40.1 ±0.01 |
| R30A | 41.3 ±0.05 |
| R37A | 47.8 ±0.03 |
| T43A | 42.3 ±0.07 |
| L61A | 42.4 ±0.13 |
| F64A | 43.3 ±0.16 |
| N65A | 46.9 ±0.14 |
| Y81A | 44.9 ±0.12 |
| Y131A | 46.7 ±0.25 |

**References.**

Pagliai, F. A., Gardner, C. L., Bojilova, L., Sarnegrim, A., Tamayo, C., Potts, A. H., et al. (2014). The Transcriptional Activator LdtR from “Candidatus Liberibacter asiaticus” Mediates Osmotic Stress Tolerance. *PLoS Pathog.* 10, e1004101. doi:10.1371/journal.ppat.1004101.

Sievers, F., Wilm, A., Dineen, D., Gibson, T. J., Karplus, K., Li, W., et al. (2011). Fast, scalable generation of high-quality protein multiple sequence alignments using Clustal Omega. *Mol. Syst. Biol.* 7. doi:10.1038/msb.2011.75.
