# Supplementary material for: Identification of a Ligand Binding Pocket in LdtR from Liberibacter asiaticus
Source: Front Microbiol. 2015 Nov 25;6:1314. doi: 10.3389/fmicb.2015.01314 (PMC4658428; doi:10.3389/fmicb.2015.01314)
Supplement: Supplementary file 2 [file Data_Sheet_2.DOCX]

ATOM 1 N ILE A 21 7.597 42.189 17.303 1.00 0.63 N

ATOM 2 CA ILE A 21 8.460 41.029 17.625 1.00 0.63 C

ATOM 3 C ILE A 21 7.643 39.772 17.984 1.00 0.63 C

ATOM 4 O ILE A 21 7.966 38.665 17.564 1.00 0.63 O

ATOM 5 CB ILE A 21 9.402 41.367 18.795 1.00 0.63 C

ATOM 6 CG1 ILE A 21 8.610 41.697 20.074 1.00 0.63 C

ATOM 7 CG2 ILE A 21 10.343 42.515 18.396 1.00 0.63 C

ATOM 8 CD1 ILE A 21 9.476 41.908 21.313 1.00 0.63 C

ATOM 9 N SER A 22 6.547 39.991 18.714 1.00 0.75 N

ATOM 10 CA SER A 22 5.675 38.889 19.150 1.00 0.75 C

ATOM 11 C SER A 22 4.880 38.334 17.967 1.00 0.75 C

ATOM 12 O SER A 22 4.664 37.133 17.868 1.00 0.75 O

ATOM 13 CB SER A 22 4.717 39.352 20.248 1.00 0.75 C

ATOM 14 OG SER A 22 3.972 38.220 20.685 1.00 0.75 O

ATOM 15 N GLY A 23 4.492 39.240 17.057 1.00 0.80 N

ATOM 16 CA GLY A 23 3.815 38.865 15.800 1.00 0.80 C

ATOM 17 C GLY A 23 4.734 37.981 14.935 1.00 0.80 C

ATOM 18 O GLY A 23 4.363 37.022 14.268 1.00 0.80 O

ATOM 19 N LEU A 24 6.003 38.328 14.920 1.00 0.71 N

ATOM 20 CA LEU A 24 6.984 37.561 14.145 1.00 0.71 C

ATOM 21 C LEU A 24 7.279 36.212 14.821 1.00 0.71 C

ATOM 22 O LEU A 24 7.379 35.185 14.151 1.00 0.71 O

ATOM 23 CB LEU A 24 8.201 38.466 13.958 1.00 0.71 C

ATOM 24 CG LEU A 24 9.302 37.811 13.144 1.00 0.71 C

ATOM 25 CD1 LEU A 24 10.188 38.824 12.427 1.00 0.71 C

ATOM 26 CD2 LEU A 24 10.181 37.023 14.118 1.00 0.71 C

ATOM 27 N TYR A 25 7.393 36.239 16.145 1.00 0.71 N

ATOM 28 CA TYR A 25 7.671 35.026 16.926 1.00 0.71 C

ATOM 29 C TYR A 25 6.488 34.056 16.853 1.00 0.71 C

ATOM 30 O TYR A 25 6.672 32.856 16.675 1.00 0.71 O

ATOM 31 CB TYR A 25 7.968 35.401 18.375 1.00 0.71 C

ATOM 32 CG TYR A 25 8.316 34.159 19.175 1.00 0.71 C

ATOM 33 CD1 TYR A 25 9.619 33.683 19.110 1.00 0.71 C

ATOM 34 CD2 TYR A 25 7.327 33.617 19.988 1.00 0.71 C

ATOM 35 CE1 TYR A 25 9.960 32.673 19.934 1.00 0.71 C

ATOM 36 CE2 TYR A 25 7.632 32.639 20.846 1.00 0.71 C

ATOM 37 CZ TYR A 25 8.914 32.306 20.718 1.00 0.71 C

ATOM 38 OH TYR A 25 8.895 31.159 20.977 1.00 0.71 O

ATOM 39 N VAL A 26 5.278 34.607 16.908 1.00 0.76 N

ATOM 40 CA VAL A 26 4.048 33.809 16.763 1.00 0.76 C

ATOM 41 C VAL A 26 4.023 33.080 15.414 1.00 0.76 C

ATOM 42 O VAL A 26 3.663 31.909 15.356 1.00 0.76 O

ATOM 43 CB VAL A 26 2.785 34.673 16.954 1.00 0.76 C

ATOM 44 CG1 VAL A 26 2.582 35.726 15.891 1.00 0.76 C

ATOM 45 CG2 VAL A 26 1.504 33.855 16.851 1.00 0.76 C

ATOM 46 N GLU A 27 4.440 33.790 14.370 1.00 0.72 N

ATOM 47 CA GLU A 27 4.495 33.224 13.024 1.00 0.72 C

ATOM 48 C GLU A 27 5.470 32.039 12.995 1.00 0.72 C

ATOM 49 O GLU A 27 5.293 31.033 12.318 1.00 0.72 O

ATOM 50 CB GLU A 27 4.882 34.334 12.049 1.00 0.72 C

ATOM 51 CG GLU A 27 5.086 33.865 10.606 1.00 0.72 C

ATOM 52 CD GLU A 27 6.181 32.882 10.281 1.00 0.72 C

ATOM 53 OE1 GLU A 27 7.220 33.143 10.904 1.00 0.72 O

ATOM 54 OE2 GLU A 27 5.964 32.004 9.415 1.00 0.72 O1-

ATOM 55 N CYS A 28 6.572 32.216 13.687 1.00 0.76 N

ATOM 56 CA CYS A 28 7.587 31.169 13.798 1.00 0.76 C

ATOM 57 C CYS A 28 7.032 29.969 14.580 1.00 0.76 C

ATOM 58 O CYS A 28 7.089 28.827 14.134 1.00 0.76 O

ATOM 59 CB CYS A 28 8.758 31.830 14.517 1.00 0.76 C

ATOM 60 SG CYS A 28 10.191 30.727 14.693 1.00 0.76 S

ATOM 61 N LEU A 29 6.368 30.269 15.685 1.00 0.77 N

ATOM 62 CA LEU A 29 5.784 29.228 16.521 1.00 0.77 C

ATOM 63 C LEU A 29 4.705 28.465 15.748 1.00 0.77 C

ATOM 64 O LEU A 29 4.632 27.253 15.853 1.00 0.77 O

ATOM 65 CB LEU A 29 5.257 29.893 17.775 1.00 0.77 C

ATOM 66 CG LEU A 29 4.687 28.820 18.652 1.00 0.77 C

ATOM 67 CD1 LEU A 29 4.903 29.350 20.020 1.00 0.77 C

ATOM 68 CD2 LEU A 29 3.199 28.755 18.338 1.00 0.77 C

ATOM 69 N ARG A 30 3.890 29.170 14.979 1.00 0.71 N

ATOM 70 CA ARG A 30 2.831 28.540 14.172 1.00 0.71 C

ATOM 71 C ARG A 30 3.391 27.579 13.113 1.00 0.71 C

ATOM 72 O ARG A 30 2.722 26.629 12.711 1.00 0.71 O

ATOM 73 CB ARG A 30 1.919 29.592 13.535 1.00 0.71 C

ATOM 74 CG ARG A 30 2.636 30.463 12.515 1.00 0.71 C

ATOM 75 CD ARG A 30 1.762 31.545 11.909 1.00 0.71 C

ATOM 76 NE ARG A 30 2.511 32.393 10.960 1.00 0.71 N

ATOM 77 CZ ARG A 30 1.968 33.413 10.275 1.00 0.71 C

ATOM 78 NH1 ARG A 30 0.692 33.696 10.419 1.00 0.71 N1+

ATOM 79 NH2 ARG A 30 2.660 34.157 9.418 1.00 0.71 N

ATOM 80 N LEU A 31 4.619 27.847 12.675 1.00 0.77 N

ATOM 81 CA LEU A 31 5.280 26.996 11.677 1.00 0.77 C

ATOM 82 C LEU A 31 5.688 25.625 12.215 1.00 0.77 C

ATOM 83 O LEU A 31 5.919 24.688 11.454 1.00 0.77 O

ATOM 84 CB LEU A 31 6.472 27.738 11.095 1.00 0.77 C

ATOM 85 CG LEU A 31 7.174 27.021 9.919 1.00 0.77 C

ATOM 86 CD1 LEU A 31 7.929 27.996 9.012 1.00 0.77 C

ATOM 87 CD2 LEU A 31 8.180 25.970 10.366 1.00 0.77 C

ATOM 88 N VAL A 32 5.687 25.477 13.532 1.00 0.80 N

ATOM 89 CA VAL A 32 6.091 24.213 14.175 1.00 0.80 C

ATOM 90 C VAL A 32 5.332 22.999 13.612 1.00 0.80 C

ATOM 91 O VAL A 32 5.936 21.958 13.352 1.00 0.80 O

ATOM 92 CB VAL A 32 5.893 24.310 15.698 1.00 0.80 C

ATOM 93 CG1 VAL A 32 4.424 24.476 16.108 1.00 0.80 C

ATOM 94 CG2 VAL A 32 6.459 23.069 16.392 1.00 0.80 C

ATOM 95 N GLU A 33 4.018 23.175 13.453 1.00 0.75 N

ATOM 96 CA GLU A 33 3.141 22.123 12.953 1.00 0.75 C

ATOM 97 C GLU A 33 3.525 21.629 11.539 1.00 0.75 C

ATOM 98 O GLU A 33 3.764 20.480 11.165 1.00 0.75 O

ATOM 99 CB GLU A 33 1.658 22.440 13.177 1.00 0.75 C

ATOM 100 CG GLU A 33 0.738 21.278 12.760 1.00 0.75 C

ATOM 101 CD GLU A 33 0.734 21.044 11.239 1.00 0.75 C

ATOM 102 OE1 GLU A 33 0.886 22.040 10.494 1.00 0.75 O

ATOM 103 OE2 GLU A 33 0.491 19.887 10.829 1.00 0.75 O1-

ATOM 104 N ARG A 34 3.616 22.566 10.647 1.00 0.73 N

ATOM 105 CA ARG A 34 3.956 22.180 9.271 1.00 0.73 C

ATOM 106 C ARG A 34 5.405 21.688 9.171 1.00 0.73 C

ATOM 107 O ARG A 34 5.712 20.770 8.415 1.00 0.73 O

ATOM 108 CB ARG A 34 3.675 23.328 8.332 1.00 0.73 C

ATOM 109 CG ARG A 34 4.492 24.572 8.622 1.00 0.73 C

ATOM 110 CD ARG A 34 4.141 25.605 7.561 1.00 0.73 C

ATOM 111 NE ARG A 34 4.897 26.848 7.812 1.00 0.73 N

ATOM 112 CZ ARG A 34 4.514 27.829 8.616 1.00 0.73 C

ATOM 113 NH1 ARG A 34 3.393 27.700 9.293 1.00 0.73 N1+

ATOM 114 NH2 ARG A 34 5.238 28.936 8.751 1.00 0.73 N

ATOM 115 N LEU A 35 6.242 22.228 10.055 1.00 0.77 N

ATOM 116 CA LEU A 35 7.656 21.850 10.115 1.00 0.77 C

ATOM 117 C LEU A 35 7.817 20.381 10.506 1.00 0.77 C

ATOM 118 O LEU A 35 8.568 19.644 9.870 1.00 0.77 O

ATOM 119 CB LEU A 35 8.400 22.719 11.129 1.00 0.77 C

ATOM 120 CG LEU A 35 9.874 22.328 11.280 1.00 0.77 C

ATOM 121 CD1 LEU A 35 10.605 22.401 9.939 1.00 0.77 C

ATOM 122 CD2 LEU A 35 10.559 23.237 12.296 1.00 0.77 C

ATOM 123 N HIS A 36 7.056 19.960 11.510 1.00 0.78 N

ATOM 124 CA HIS A 36 7.128 18.568 11.979 1.00 0.78 C

ATOM 125 C HIS A 36 6.622 17.613 10.891 1.00 0.78 C

ATOM 126 O HIS A 36 7.214 16.561 10.658 1.00 0.78 O

ATOM 127 CB HIS A 36 6.342 18.365 13.272 1.00 0.78 C

ATOM 128 CG HIS A 36 4.839 18.544 13.097 1.00 0.78 C

ATOM 129 CD2 HIS A 36 4.149 19.476 13.695 1.00 0.78 C

ATOM 130 ND1 HIS A 36 3.967 17.733 12.506 1.00 0.78 N

ATOM 131 CE1 HIS A 36 2.740 18.222 12.616 1.00 0.78 C

ATOM 132 NE2 HIS A 36 2.865 19.295 13.382 1.00 0.78 N

ATOM 133 N ARG A 37 5.552 18.034 10.209 1.00 0.70 N

ATOM 134 CA ARG A 37 5.009 17.292 9.062 1.00 0.70 C

ATOM 135 C ARG A 37 6.114 17.093 8.017 1.00 0.70 C

ATOM 136 O ARG A 37 6.375 15.975 7.576 1.00 0.70 O

ATOM 137 CB ARG A 37 3.808 18.045 8.471 1.00 0.70 C

ATOM 138 CG ARG A 37 3.653 17.794 6.960 1.00 0.70 C

ATOM 139 CD ARG A 37 2.363 18.309 6.348 1.00 0.70 C

ATOM 140 NE ARG A 37 2.174 19.741 6.623 1.00 0.70 N

ATOM 141 CZ ARG A 37 2.631 20.757 5.889 1.00 0.70 C

ATOM 142 NH1 ARG A 37 3.382 20.559 4.811 1.00 0.70 N1+

ATOM 143 NH2 ARG A 37 2.217 21.985 6.159 1.00 0.70 N

ATOM 144 N SER A 38 6.729 18.213 7.655 1.00 0.75 N

ATOM 145 CA SER A 38 7.716 18.240 6.577 1.00 0.75 C

ATOM 146 C SER A 38 8.960 17.432 6.941 1.00 0.75 C

ATOM 147 O SER A 38 9.490 16.717 6.096 1.00 0.75 O

ATOM 148 CB SER A 38 8.088 19.687 6.322 1.00 0.75 C

ATOM 149 OG SER A 38 8.957 19.683 5.215 1.00 0.75 O

ATOM 150 N LEU A 39 9.354 17.489 8.209 1.00 0.72 N

ATOM 151 CA LEU A 39 10.499 16.712 8.720 1.00 0.72 C

ATOM 152 C LEU A 39 10.229 15.231 8.536 1.00 0.72 C

ATOM 153 O LEU A 39 11.068 14.451 8.112 1.00 0.72 O

ATOM 154 CB LEU A 39 10.675 16.873 10.229 1.00 0.72 C

ATOM 155 CG LEU A 39 12.034 16.365 10.741 1.00 0.72 C

ATOM 156 CD1 LEU A 39 12.505 16.874 12.100 1.00 0.72 C

ATOM 157 CD2 LEU A 39 11.730 14.972 11.230 1.00 0.72 C

ATOM 158 N LEU A 40 9.045 14.834 8.957 1.00 0.69 N

ATOM 159 CA LEU A 40 8.669 13.422 8.896 1.00 0.69 C

ATOM 160 C LEU A 40 8.653 12.942 7.439 1.00 0.69 C

ATOM 161 O LEU A 40 9.177 11.871 7.132 1.00 0.69 O

ATOM 162 CB LEU A 40 7.323 13.257 9.599 1.00 0.69 C

ATOM 163 CG LEU A 40 6.888 11.791 9.683 1.00 0.69 C

ATOM 164 CD1 LEU A 40 5.918 11.617 10.848 1.00 0.69 C

ATOM 165 CD2 LEU A 40 6.187 11.339 8.397 1.00 0.69 C

ATOM 166 N ASP A 41 8.064 13.756 6.567 1.00 0.68 N

ATOM 167 CA ASP A 41 8.016 13.433 5.139 1.00 0.68 C

ATOM 168 C ASP A 41 9.415 13.405 4.499 1.00 0.68 C

ATOM 169 O ASP A 41 9.737 12.628 3.614 1.00 0.68 O

ATOM 170 CB ASP A 41 7.093 14.405 4.407 1.00 0.68 C

ATOM 171 CG ASP A 41 5.636 14.369 4.844 1.00 0.68 C

ATOM 172 OD1 ASP A 41 5.028 13.299 4.839 1.00 0.68 O

ATOM 173 OD2 ASP A 41 5.154 15.404 5.319 1.00 0.68 O1-

ATOM 174 N VAL A 42 10.285 14.295 4.933 1.00 0.64 N

ATOM 175 CA VAL A 42 11.636 14.387 4.352 1.00 0.64 C

ATOM 176 C VAL A 42 12.452 13.175 4.777 1.00 0.64 C

ATOM 177 O VAL A 42 13.146 12.545 3.981 1.00 0.64 O

ATOM 178 CB VAL A 42 12.264 15.728 4.766 1.00 0.64 C

ATOM 179 CG1 VAL A 42 12.480 15.922 6.243 1.00 0.64 C

ATOM 180 CG2 VAL A 42 13.726 15.778 4.461 1.00 0.64 C

ATOM 181 N THR A 43 12.331 12.889 6.060 1.00 0.70 N

ATOM 182 CA THR A 43 13.044 11.776 6.659 1.00 0.70 C

ATOM 183 C THR A 43 12.601 10.467 5.987 1.00 0.70 C

ATOM 184 O THR A 43 13.370 9.571 5.683 1.00 0.70 O

ATOM 185 CB THR A 43 12.676 11.802 8.130 1.00 0.70 C

ATOM 186 CG2 THR A 43 13.447 10.735 8.772 1.00 0.70 C

ATOM 187 OG1 THR A 43 13.229 12.910 8.819 1.00 0.70 O

ATOM 188 N ARG A 44 11.319 10.329 5.733 1.00 0.59 N

ATOM 189 CA ARG A 44 10.809 9.098 5.107 1.00 0.59 C

ATOM 190 C ARG A 44 11.416 8.867 3.711 1.00 0.59 C

ATOM 191 O ARG A 44 11.704 7.745 3.321 1.00 0.59 O

ATOM 192 CB ARG A 44 9.283 9.111 5.057 1.00 0.59 C

ATOM 193 CG ARG A 44 8.757 10.175 4.111 1.00 0.59 C

ATOM 194 CD ARG A 44 7.250 10.296 4.069 1.00 0.59 C

ATOM 195 NE ARG A 44 6.816 11.393 3.178 1.00 0.59 N

ATOM 196 CZ ARG A 44 5.533 11.637 2.897 1.00 0.59 C

ATOM 197 NH1 ARG A 44 4.578 10.890 3.459 1.00 0.59 N1+

ATOM 198 NH2 ARG A 44 5.187 12.701 2.170 1.00 0.59 N

ATOM 199 N ASP A 45 11.723 9.941 2.996 1.00 0.62 N

ATOM 200 CA ASP A 45 12.366 9.815 1.675 1.00 0.62 C

ATOM 201 C ASP A 45 13.740 9.146 1.797 1.00 0.62 C

ATOM 202 O ASP A 45 14.087 8.265 1.014 1.00 0.62 O

ATOM 203 CB ASP A 45 12.530 11.189 1.024 1.00 0.62 C

ATOM 204 CG ASP A 45 11.186 11.823 0.666 1.00 0.62 C

ATOM 205 OD1 ASP A 45 10.448 11.187 -0.118 1.00 0.62 O

ATOM 206 OD2 ASP A 45 10.912 12.896 1.239 1.00 0.62 O1-

ATOM 207 N GLU A 46 14.454 9.515 2.858 1.00 0.66 N

ATOM 208 CA GLU A 46 15.794 8.968 3.114 1.00 0.66 C

ATOM 209 C GLU A 46 15.747 7.534 3.617 1.00 0.66 C

ATOM 210 O GLU A 46 16.539 6.676 3.231 1.00 0.66 O

ATOM 211 CB GLU A 46 16.581 9.856 4.079 1.00 0.66 C

ATOM 212 CG GLU A 46 15.996 9.902 5.483 1.00 0.66 C

ATOM 213 CD GLU A 46 16.693 10.796 6.474 1.00 0.66 C

ATOM 214 OE1 GLU A 46 17.834 10.413 6.779 1.00 0.66 O

ATOM 215 OE2 GLU A 46 16.096 11.821 6.866 1.00 0.66 O1-

ATOM 216 N PHE A 47 14.746 7.255 4.414 1.00 0.59 N

ATOM 217 CA PHE A 47 14.808 5.938 4.983 1.00 0.59 C

ATOM 218 C PHE A 47 14.193 4.830 4.128 1.00 0.59 C

ATOM 219 O PHE A 47 14.704 3.716 4.162 1.00 0.59 O

ATOM 220 CB PHE A 47 14.191 6.188 6.280 1.00 0.59 C

ATOM 221 CG PHE A 47 14.167 6.927 7.524 1.00 0.59 C

ATOM 222 CD1 PHE A 47 15.457 7.052 7.767 1.00 0.59 C

ATOM 223 CD2 PHE A 47 13.131 7.379 8.321 1.00 0.59 C

ATOM 224 CE1 PHE A 47 15.741 7.772 8.875 1.00 0.59 C

ATOM 225 CE2 PHE A 47 13.508 8.024 9.479 1.00 0.59 C

ATOM 226 CZ PHE A 47 14.839 8.265 9.719 1.00 0.59 C

ATOM 227 N GLU A 48 13.296 5.203 3.213 1.00 0.58 N

ATOM 228 CA GLU A 48 12.782 4.265 2.195 1.00 0.58 C

ATOM 229 C GLU A 48 13.896 3.819 1.253 1.00 0.58 C

ATOM 230 O GLU A 48 13.835 2.701 0.751 1.00 0.58 O

ATOM 231 CB GLU A 48 11.622 4.887 1.410 1.00 0.58 C

ATOM 232 CG GLU A 48 10.892 3.905 0.495 1.00 0.58 C

ATOM 233 CD GLU A 48 11.725 3.690 -0.772 1.00 0.58 C

ATOM 234 OE1 GLU A 48 12.437 4.646 -1.163 1.00 0.58 O

ATOM 235 OE2 GLU A 48 11.639 2.570 -1.307 1.00 0.58 O1-

ATOM 236 N ARG A 49 14.873 4.692 1.022 1.00 0.55 N

ATOM 237 CA ARG A 49 16.034 4.313 0.198 1.00 0.55 C

ATOM 238 C ARG A 49 16.820 3.151 0.816 1.00 0.55 C

ATOM 239 O ARG A 49 17.390 2.336 0.098 1.00 0.55 O

ATOM 240 CB ARG A 49 16.973 5.489 -0.081 1.00 0.55 C

ATOM 241 CG ARG A 49 16.438 6.358 -1.222 1.00 0.55 C

ATOM 242 CD ARG A 49 17.358 7.540 -1.553 1.00 0.55 C

ATOM 243 NE ARG A 49 18.601 7.143 -2.249 1.00 0.55 N

ATOM 244 CZ ARG A 49 18.794 7.194 -3.574 1.00 0.55 C

ATOM 245 NH1 ARG A 49 17.801 7.545 -4.386 1.00 0.55 N1+

ATOM 246 NH2 ARG A 49 20.008 7.023 -4.091 1.00 0.55 N

ATOM 247 N GLN A 50 16.788 3.063 2.144 1.00 0.64 N

ATOM 248 CA GLN A 50 17.412 1.945 2.864 1.00 0.64 C

ATOM 249 C GLN A 50 16.385 0.885 3.319 1.00 0.64 C

ATOM 250 O GLN A 50 16.639 0.012 4.154 1.00 0.64 O

ATOM 251 CB GLN A 50 18.144 2.550 4.053 1.00 0.64 C

ATOM 252 CG GLN A 50 18.936 1.477 4.767 1.00 0.64 C

ATOM 253 CD GLN A 50 20.024 0.774 3.981 1.00 0.64 C

ATOM 254 NE2 GLN A 50 19.909 -0.530 3.785 1.00 0.64 N

ATOM 255 OE1 GLN A 50 20.980 1.429 3.610 1.00 0.64 O

ATOM 256 N GLY A 51 15.226 0.918 2.671 1.00 0.59 N

ATOM 257 CA GLY A 51 14.147 -0.034 2.965 1.00 0.59 C

ATOM 258 C GLY A 51 13.929 -0.953 1.767 1.00 0.59 C

ATOM 259 O GLY A 51 14.252 -0.610 0.633 1.00 0.59 O

ATOM 260 N ARG A 52 13.402 -2.137 2.055 1.00 0.48 N

ATOM 261 CA ARG A 52 13.114 -3.134 1.006 1.00 0.48 C

ATOM 262 C ARG A 52 11.758 -2.866 0.335 1.00 0.48 C

ATOM 263 O ARG A 52 11.610 -2.988 -0.874 1.00 0.48 O

ATOM 264 CB ARG A 52 13.166 -4.553 1.584 1.00 0.48 C

ATOM 265 CG ARG A 52 12.118 -4.786 2.677 1.00 0.48 C

ATOM 266 CD ARG A 52 12.177 -6.216 3.176 1.00 0.48 C

ATOM 267 NE ARG A 52 11.148 -6.435 4.207 1.00 0.48 N

ATOM 268 CZ ARG A 52 10.968 -7.590 4.849 1.00 0.48 C

ATOM 269 NH1 ARG A 52 11.737 -8.641 4.577 1.00 0.48 N1+

ATOM 270 NH2 ARG A 52 10.017 -7.705 5.770 1.00 0.48 N

ATOM 271 N SER A 53 10.768 -2.556 1.168 1.00 0.50 N

ATOM 272 CA SER A 53 9.399 -2.269 0.710 1.00 0.50 C

ATOM 273 C SER A 53 8.890 -0.944 1.283 1.00 0.50 C

ATOM 274 O SER A 53 7.915 -0.366 0.813 1.00 0.50 O

ATOM 275 CB SER A 53 8.464 -3.418 1.095 1.00 0.50 C

ATOM 276 OG SER A 53 7.158 -3.156 0.581 1.00 0.50 O

ATOM 277 N ASP A 54 9.522 -0.510 2.366 1.00 0.52 N

ATOM 278 CA ASP A 54 9.153 0.728 3.061 1.00 0.52 C

ATOM 279 C ASP A 54 10.398 1.265 3.756 1.00 0.52 C

ATOM 280 O ASP A 54 11.353 0.527 3.978 1.00 0.52 O

ATOM 281 CB ASP A 54 8.045 0.418 4.079 1.00 0.52 C

ATOM 282 CG ASP A 54 8.476 -0.596 5.151 1.00 0.52 C

ATOM 283 OD1 ASP A 54 9.669 -0.634 5.506 1.00 0.52 O

ATOM 284 OD2 ASP A 54 7.592 -1.322 5.637 1.00 0.52 O1-

ATOM 285 N VAL A 55 10.301 2.497 4.194 1.00 0.57 N

ATOM 286 CA VAL A 55 11.391 3.103 4.946 1.00 0.57 C

ATOM 287 C VAL A 55 11.675 2.239 6.209 1.00 0.57 C

ATOM 288 O VAL A 55 12.616 1.449 6.172 1.00 0.57 O

ATOM 289 CB VAL A 55 10.769 4.454 5.220 1.00 0.57 C

ATOM 290 CG1 VAL A 55 11.559 5.208 6.153 1.00 0.57 C

ATOM 291 CG2 VAL A 55 10.415 5.496 4.230 1.00 0.57 C

ATOM 292 N ASN A 56 10.923 2.377 7.321 1.00 0.64 N

ATOM 293 CA ASN A 56 9.884 3.369 7.686 1.00 0.64 C

ATOM 294 C ASN A 56 10.431 4.390 8.705 1.00 0.64 C

ATOM 295 O ASN A 56 11.508 4.155 9.246 1.00 0.64 O

ATOM 296 CB ASN A 56 8.498 2.837 7.948 1.00 0.64 C

ATOM 297 CG ASN A 56 7.524 4.009 8.047 1.00 0.64 C

ATOM 298 ND2 ASN A 56 6.758 4.205 7.003 1.00 0.64 N

ATOM 299 OD1 ASN A 56 7.442 4.705 9.050 1.00 0.64 O

ATOM 300 N ALA A 57 9.891 5.614 8.704 1.00 0.73 N

ATOM 301 CA ALA A 57 10.267 6.614 9.721 1.00 0.73 C

ATOM 302 C ALA A 57 10.181 6.040 11.140 1.00 0.73 C

ATOM 303 O ALA A 57 11.091 6.227 11.945 1.00 0.73 O

ATOM 304 CB ALA A 57 9.369 7.845 9.607 1.00 0.73 C

ATOM 305 N VAL A 58 9.126 5.266 11.395 1.00 0.75 N

ATOM 306 CA VAL A 58 8.933 4.607 12.700 1.00 0.75 C

ATOM 307 C VAL A 58 10.067 3.614 12.989 1.00 0.75 C

ATOM 308 O VAL A 58 10.654 3.634 14.066 1.00 0.75 O

ATOM 309 CB VAL A 58 7.562 3.912 12.757 1.00 0.75 C

ATOM 310 CG1 VAL A 58 7.396 2.805 11.703 1.00 0.75 C

ATOM 311 CG2 VAL A 58 7.297 3.363 14.163 1.00 0.75 C

ATOM 312 N GLN A 59 10.381 2.801 11.985 1.00 0.75 N

ATOM 313 CA GLN A 59 11.435 1.785 12.088 1.00 0.75 C

ATOM 314 C GLN A 59 12.795 2.434 12.320 1.00 0.75 C

ATOM 315 O GLN A 59 13.551 1.992 13.181 1.00 0.75 O

ATOM 316 CB GLN A 59 11.470 0.947 10.818 1.00 0.75 C

ATOM 317 CG GLN A 59 10.171 0.162 10.649 1.00 0.75 C

ATOM 318 CD GLN A 59 10.103 -0.533 9.295 1.00 0.75 C

ATOM 319 NE2 GLN A 59 8.987 -1.166 9.067 1.00 0.75 N

ATOM 320 OE1 GLN A 59 10.942 -0.396 8.420 1.00 0.75 O

ATOM 321 N ALA A 60 13.008 3.566 11.659 1.00 0.79 N

ATOM 322 CA ALA A 60 14.244 4.331 11.851 1.00 0.79 C

ATOM 323 C ALA A 60 14.383 4.869 13.273 1.00 0.79 C

ATOM 324 O ALA A 60 15.465 4.798 13.851 1.00 0.79 O

ATOM 325 CB ALA A 60 14.231 5.499 10.912 1.00 0.79 C

ATOM 326 N LEU A 61 13.257 5.273 13.858 1.00 0.77 N

ATOM 327 CA LEU A 61 13.243 5.799 15.229 1.00 0.77 C

ATOM 328 C LEU A 61 13.687 4.714 16.213 1.00 0.77 C

ATOM 329 O LEU A 61 14.530 4.980 17.061 1.00 0.77 O

ATOM 330 CB LEU A 61 11.848 6.343 15.554 1.00 0.77 C

ATOM 331 CG LEU A 61 11.781 7.028 16.924 1.00 0.77 C

ATOM 332 CD1 LEU A 61 10.629 8.029 16.947 1.00 0.77 C

ATOM 333 CD2 LEU A 61 11.486 6.015 18.027 1.00 0.77 C

ATOM 334 N LEU A 62 13.249 3.486 15.972 1.00 0.79 N

ATOM 335 CA LEU A 62 13.644 2.371 16.844 1.00 0.79 C

ATOM 336 C LEU A 62 15.094 1.956 16.621 1.00 0.79 C

ATOM 337 O LEU A 62 15.786 1.634 17.584 1.00 0.79 O

ATOM 338 CB LEU A 62 12.746 1.197 16.580 1.00 0.79 C

ATOM 339 CG LEU A 62 12.915 0.017 17.502 1.00 0.79 C

ATOM 340 CD1 LEU A 62 11.557 -0.648 17.442 1.00 0.79 C

ATOM 341 CD2 LEU A 62 14.002 -0.926 16.992 1.00 0.79 C

ATOM 342 N LEU A 63 15.525 1.968 15.364 1.00 0.80 N

ATOM 343 CA LEU A 63 16.924 1.662 15.034 1.00 0.80 C

ATOM 344 C LEU A 63 17.878 2.670 15.671 1.00 0.80 C

ATOM 345 O LEU A 63 18.942 2.334 16.182 1.00 0.80 O

ATOM 346 CB LEU A 63 17.135 1.707 13.519 1.00 0.80 C

ATOM 347 CG LEU A 63 16.379 0.605 12.795 1.00 0.80 C

ATOM 348 CD1 LEU A 63 16.597 0.643 11.287 1.00 0.80 C

ATOM 349 CD2 LEU A 63 17.102 -0.647 13.225 1.00 0.80 C

ATOM 350 N PHE A 64 17.442 3.919 15.673 1.00 0.74 N

ATOM 351 CA PHE A 64 18.233 5.014 16.248 1.00 0.74 C

ATOM 352 C PHE A 64 18.320 4.867 17.768 1.00 0.74 C

ATOM 353 O PHE A 64 19.394 4.982 18.355 1.00 0.74 O

ATOM 354 CB PHE A 64 17.610 6.355 15.865 1.00 0.74 C

ATOM 355 CG PHE A 64 18.440 7.517 16.414 1.00 0.74 C

ATOM 356 CD1 PHE A 64 19.588 7.925 15.744 1.00 0.74 C

ATOM 357 CD2 PHE A 64 18.039 8.168 17.574 1.00 0.74 C

ATOM 358 CE1 PHE A 64 20.335 8.987 16.236 1.00 0.74 C

ATOM 359 CE2 PHE A 64 18.785 9.232 18.063 1.00 0.74 C

ATOM 360 CZ PHE A 64 19.933 9.641 17.396 1.00 0.74 C

ATOM 361 N ASN A 65 17.188 4.522 18.370 1.00 0.73 N

ATOM 362 CA ASN A 65 17.139 4.300 19.817 1.00 0.73 C

ATOM 363 C ASN A 65 18.045 3.155 20.277 1.00 0.73 C

ATOM 364 O ASN A 65 18.791 3.309 21.241 1.00 0.73 O

ATOM 365 CB ASN A 65 15.713 3.975 20.181 1.00 0.73 C

ATOM 366 CG ASN A 65 14.748 5.129 20.071 1.00 0.73 C

ATOM 367 ND2 ASN A 65 15.015 6.199 20.778 1.00 0.73 N

ATOM 368 OD1 ASN A 65 13.741 5.008 19.414 1.00 0.73 O

ATOM 369 N ILE A 66 18.053 2.071 19.514 1.00 0.72 N

ATOM 370 CA ILE A 66 18.920 0.928 19.845 1.00 0.72 C

ATOM 371 C ILE A 66 20.406 1.225 19.599 1.00 0.72 C

ATOM 372 O ILE A 66 21.285 0.644 20.234 1.00 0.72 O

ATOM 373 CB ILE A 66 18.467 -0.339 19.114 1.00 0.72 C

ATOM 374 CG1 ILE A 66 19.325 -1.558 19.442 1.00 0.72 C

ATOM 375 CG2 ILE A 66 18.440 -0.077 17.634 1.00 0.72 C

ATOM 376 CD1 ILE A 66 19.209 -1.813 20.937 1.00 0.72 C

ATOM 377 N GLY A 67 20.646 2.083 18.604 1.00 0.79 N

ATOM 378 CA GLY A 67 22.009 2.538 18.288 1.00 0.79 C

ATOM 379 C GLY A 67 22.579 3.300 19.486 1.00 0.79 C

ATOM 380 O GLY A 67 23.737 3.126 19.856 1.00 0.79 O

ATOM 381 N ASP A 68 21.701 4.081 20.111 1.00 0.67 N

ATOM 382 CA ASP A 68 22.064 4.901 21.274 1.00 0.67 C

ATOM 383 C ASP A 68 22.410 4.026 22.487 1.00 0.67 C

ATOM 384 O ASP A 68 23.272 4.371 23.291 1.00 0.67 O

ATOM 385 CB ASP A 68 20.923 5.867 21.601 1.00 0.67 C

ATOM 386 CG ASP A 68 21.320 6.891 22.675 1.00 0.67 C

ATOM 387 OD1 ASP A 68 22.154 6.580 23.545 1.00 0.67 O

ATOM 388 OD2 ASP A 68 20.781 8.012 22.608 1.00 0.67 O1-

ATOM 389 N LEU A 69 21.716 2.904 22.615 1.00 0.68 N

ATOM 390 CA LEU A 69 21.931 1.993 23.743 1.00 0.68 C

ATOM 391 C LEU A 69 21.163 0.690 23.456 1.00 0.68 C

ATOM 392 O LEU A 69 20.201 0.671 22.694 1.00 0.68 O

ATOM 393 CB LEU A 69 21.504 2.739 25.032 1.00 0.68 C

ATOM 394 CG LEU A 69 21.598 1.889 26.299 1.00 0.68 C

ATOM 395 CD1 LEU A 69 23.068 1.771 26.634 1.00 0.68 C

ATOM 396 CD2 LEU A 69 20.910 2.467 27.531 1.00 0.68 C

ATOM 397 N GLU A 70 21.571 -0.399 24.084 1.00 0.71 N

ATOM 398 CA GLU A 70 20.792 -1.661 24.193 1.00 0.71 C

ATOM 399 C GLU A 70 19.629 -1.358 25.131 1.00 0.71 C

ATOM 400 O GLU A 70 19.882 -0.700 26.139 1.00 0.71 O

ATOM 401 CB GLU A 70 21.565 -2.790 24.897 1.00 0.71 C

ATOM 402 CG GLU A 70 21.863 -2.614 26.406 1.00 0.71 C

ATOM 403 CD GLU A 70 22.803 -1.442 26.712 1.00 0.71 C

ATOM 404 OE1 GLU A 70 23.422 -0.916 25.762 1.00 0.71 O

ATOM 405 OE2 GLU A 70 23.055 -1.200 27.908 1.00 0.71 O1-

ATOM 406 N LEU A 71 18.519 -2.068 25.075 1.00 0.79 N

ATOM 407 CA LEU A 71 17.318 -1.604 25.798 1.00 0.79 C

ATOM 408 C LEU A 71 16.163 -2.594 25.721 1.00 0.79 C

ATOM 409 O LEU A 71 16.084 -3.426 24.820 1.00 0.79 O

ATOM 410 CB LEU A 71 16.817 -0.270 25.227 1.00 0.79 C

ATOM 411 CG LEU A 71 17.544 0.979 25.727 1.00 0.79 C

ATOM 412 CD1 LEU A 71 18.642 1.400 24.849 1.00 0.79 C

ATOM 413 CD2 LEU A 71 16.907 2.159 25.079 1.00 0.79 C

ATOM 414 N THR A 72 15.312 -2.494 26.728 1.00 0.82 N

ATOM 415 CA THR A 72 14.016 -3.188 26.760 1.00 0.82 C

ATOM 416 C THR A 72 13.022 -2.327 25.971 1.00 0.82 C

ATOM 417 O THR A 72 13.257 -1.138 25.731 1.00 0.82 O

ATOM 418 CB THR A 72 13.535 -3.364 28.207 1.00 0.82 C

ATOM 419 CG2 THR A 72 14.560 -4.128 29.053 1.00 0.82 C

ATOM 420 OG1 THR A 72 13.236 -2.082 28.768 1.00 0.82 O

ATOM 421 N ALA A 73 11.883 -2.920 25.627 1.00 0.83 N

ATOM 422 CA ALA A 73 10.827 -2.200 24.888 1.00 0.83 C

ATOM 423 C ALA A 73 10.301 -1.014 25.704 1.00 0.83 C

ATOM 424 O ALA A 73 10.092 0.071 25.171 1.00 0.83 O

ATOM 425 CB ALA A 73 9.671 -3.148 24.581 1.00 0.83 C

ATOM 426 N GLY A 74 10.212 -1.243 27.020 1.00 0.85 N

ATOM 427 CA GLY A 74 9.808 -0.213 27.980 1.00 0.85 C

ATOM 428 C GLY A 74 10.726 0.996 27.988 1.00 0.85 C

ATOM 429 O GLY A 74 10.298 2.146 27.925 1.00 0.85 O

ATOM 430 N GLU A 75 12.007 0.665 28.019 1.00 0.73 N

ATOM 431 CA GLU A 75 13.073 1.676 28.006 1.00 0.73 C

ATOM 432 C GLU A 75 13.080 2.479 26.711 1.00 0.73 C

ATOM 433 O GLU A 75 13.181 3.704 26.738 1.00 0.73 O

ATOM 434 CB GLU A 75 14.441 1.030 28.190 1.00 0.73 C

ATOM 435 CG GLU A 75 14.577 0.408 29.579 1.00 0.73 C

ATOM 436 CD GLU A 75 15.957 -0.216 29.762 1.00 0.73 C

ATOM 437 OE1 GLU A 75 16.260 -1.186 29.033 1.00 0.73 O

ATOM 438 OE2 GLU A 75 16.684 0.305 30.628 1.00 0.73 O1-

ATOM 439 N LEU A 76 12.897 1.780 25.601 1.00 0.76 N

ATOM 440 CA LEU A 76 12.832 2.465 24.323 1.00 0.76 C

ATOM 441 C LEU A 76 11.619 3.404 24.316 1.00 0.76 C

ATOM 442 O LEU A 76 11.807 4.592 24.069 1.00 0.76 O

ATOM 443 CB LEU A 76 12.823 1.378 23.265 1.00 0.76 C

ATOM 444 CG LEU A 76 14.085 0.735 22.800 1.00 0.76 C

ATOM 445 CD1 LEU A 76 13.691 -0.170 21.653 1.00 0.76 C

ATOM 446 CD2 LEU A 76 14.985 1.820 22.282 1.00 0.76 C

ATOM 447 N ARG A 77 10.489 2.942 24.869 1.00 0.69 N

ATOM 448 CA ARG A 77 9.241 3.729 24.909 1.00 0.69 C

ATOM 449 C ARG A 77 9.435 5.026 25.656 1.00 0.69 C

ATOM 450 O ARG A 77 9.076 6.087 25.159 1.00 0.69 O

ATOM 451 CB ARG A 77 8.110 2.984 25.585 1.00 0.69 C

ATOM 452 CG ARG A 77 7.721 1.800 24.761 1.00 0.69 C

ATOM 453 CD ARG A 77 6.604 1.047 25.389 1.00 0.69 C

ATOM 454 NE ARG A 77 7.001 0.306 26.555 1.00 0.69 N

ATOM 455 CZ ARG A 77 6.672 0.722 27.766 1.00 0.69 C

ATOM 456 NH1 ARG A 77 5.948 1.824 27.951 1.00 0.69 N1+

ATOM 457 NH2 ARG A 77 7.097 0.037 28.804 1.00 0.69 N

ATOM 458 N SER A 78 10.108 4.897 26.790 1.00 0.76 N

ATOM 459 CA SER A 78 10.439 6.056 27.627 1.00 0.76 C

ATOM 460 C SER A 78 11.411 7.009 26.928 1.00 0.76 C

ATOM 461 O SER A 78 11.187 8.216 26.914 1.00 0.76 O

ATOM 462 CB SER A 78 11.055 5.603 28.949 1.00 0.76 C

ATOM 463 OG SER A 78 10.102 4.833 29.684 1.00 0.76 O

ATOM 464 N ARG A 79 12.431 6.445 26.283 1.00 0.69 N

ATOM 465 CA ARG A 79 13.479 7.243 25.622 1.00 0.69 C

ATOM 466 C ARG A 79 12.943 8.016 24.409 1.00 0.69 C

ATOM 467 O ARG A 79 13.317 9.163 24.177 1.00 0.69 O

ATOM 468 CB ARG A 79 14.632 6.334 25.200 1.00 0.69 C

ATOM 469 CG ARG A 79 15.772 7.149 24.582 1.00 0.69 C

ATOM 470 CD ARG A 79 16.915 6.255 24.133 1.00 0.69 C

ATOM 471 NE ARG A 79 17.541 5.632 25.305 1.00 0.69 N

ATOM 472 CZ ARG A 79 18.526 4.753 25.239 1.00 0.69 C

ATOM 473 NH1 ARG A 79 18.996 4.333 24.065 1.00 0.69 N1+

ATOM 474 NH2 ARG A 79 18.969 4.204 26.355 1.00 0.69 N

ATOM 475 N GLY A 80 12.060 7.351 23.663 1.00 0.81 N

ATOM 476 CA GLY A 80 11.510 7.927 22.431 1.00 0.81 C

ATOM 477 C GLY A 80 10.162 8.596 22.677 1.00 0.81 C

ATOM 478 O GLY A 80 9.582 9.073 21.708 1.00 0.81 O

ATOM 479 N TYR A 81 9.658 8.519 23.914 1.00 0.70 N

ATOM 480 CA TYR A 81 8.355 9.084 24.313 1.00 0.70 C

ATOM 481 C TYR A 81 7.226 8.561 23.403 1.00 0.70 C

ATOM 482 O TYR A 81 6.397 9.286 22.866 1.00 0.70 O

ATOM 483 CB TYR A 81 8.412 10.623 24.342 1.00 0.70 C

ATOM 484 CG TYR A 81 8.692 11.270 22.981 1.00 0.70 C

ATOM 485 CD1 TYR A 81 7.672 11.557 22.078 1.00 0.70 C

ATOM 486 CD2 TYR A 81 9.998 11.534 22.625 1.00 0.70 C

ATOM 487 CE1 TYR A 81 7.927 12.092 20.829 1.00 0.70 C

ATOM 488 CE2 TYR A 81 10.233 12.067 21.385 1.00 0.70 C

ATOM 489 CZ TYR A 81 9.229 12.340 20.471 1.00 0.70 C

ATOM 490 OH TYR A 81 9.487 12.866 19.253 1.00 0.70 O

ATOM 491 N TYR A 82 7.229 7.260 23.177 1.00 0.70 N

ATOM 492 CA TYR A 82 6.208 6.617 22.337 1.00 0.70 C

ATOM 493 C TYR A 82 5.459 5.569 23.160 1.00 0.70 C

ATOM 494 O TYR A 82 5.996 4.985 24.099 1.00 0.70 O

ATOM 495 CB TYR A 82 6.860 6.011 21.096 1.00 0.70 C

ATOM 496 CG TYR A 82 7.866 4.958 21.489 1.00 0.70 C

ATOM 497 CD1 TYR A 82 9.192 5.279 21.708 1.00 0.70 C

ATOM 498 CD2 TYR A 82 7.439 3.670 21.590 1.00 0.70 C

ATOM 499 CE1 TYR A 82 10.125 4.311 21.960 1.00 0.70 C

ATOM 500 CE2 TYR A 82 8.403 2.795 21.910 1.00 0.70 C

ATOM 501 CZ TYR A 82 9.700 3.031 22.040 1.00 0.70 C

ATOM 502 OH TYR A 82 10.476 1.987 22.293 1.00 0.70 O

ATOM 503 N LEU A 83 4.209 5.351 22.775 1.00 0.68 N

ATOM 504 CA LEU A 83 3.339 4.394 23.467 1.00 0.68 C

ATOM 505 C LEU A 83 3.859 2.965 23.237 1.00 0.68 C

ATOM 506 O LEU A 83 4.383 2.635 22.171 1.00 0.68 O

ATOM 507 CB LEU A 83 1.902 4.611 22.973 1.00 0.68 C

ATOM 508 CG LEU A 83 0.869 3.832 23.792 1.00 0.68 C

ATOM 509 CD1 LEU A 83 -0.522 4.430 23.655 1.00 0.68 C

ATOM 510 CD2 LEU A 83 0.680 2.460 23.222 1.00 0.68 C

ATOM 511 N GLY A 84 3.571 2.114 24.228 1.00 0.81 N

ATOM 512 CA GLY A 84 3.861 0.669 24.201 1.00 0.81 C

ATOM 513 C GLY A 84 3.483 -0.073 22.934 1.00 0.81 C

ATOM 514 O GLY A 84 4.155 -0.893 22.337 1.00 0.81 O

ATOM 515 N SER A 85 2.281 0.210 22.537 1.00 0.75 N

ATOM 516 CA SER A 85 1.675 -0.430 21.366 1.00 0.75 C

ATOM 517 C SER A 85 2.447 -0.084 20.092 1.00 0.75 C

ATOM 518 O SER A 85 2.707 -0.965 19.278 1.00 0.75 O

ATOM 519 CB SER A 85 0.225 0.007 21.220 1.00 0.75 C

ATOM 520 OG SER A 85 -0.386 -0.642 20.122 1.00 0.75 O

ATOM 521 N ASN A 86 2.890 1.170 19.995 1.00 0.74 N

ATOM 522 CA ASN A 86 3.650 1.636 18.827 1.00 0.74 C

ATOM 523 C ASN A 86 5.038 0.995 18.777 1.00 0.74 C

ATOM 524 O ASN A 86 5.482 0.495 17.743 1.00 0.74 O

ATOM 525 CB ASN A 86 3.779 3.159 18.861 1.00 0.74 C

ATOM 526 CG ASN A 86 4.487 3.686 17.610 1.00 0.74 C

ATOM 527 ND2 ASN A 86 5.148 4.812 17.771 1.00 0.74 N

ATOM 528 OD1 ASN A 86 4.453 3.094 16.540 1.00 0.74 O

ATOM 529 N VAL A 87 5.695 0.963 19.929 1.00 0.79 N

ATOM 530 CA VAL A 87 6.998 0.277 19.982 1.00 0.79 C

ATOM 531 C VAL A 87 6.932 -1.187 19.641 1.00 0.79 C

ATOM 532 O VAL A 87 7.875 -1.691 19.041 1.00 0.79 O

ATOM 533 CB VAL A 87 7.587 0.209 21.358 1.00 0.79 C

ATOM 534 CG1 VAL A 87 6.755 -0.281 22.502 1.00 0.79 C

ATOM 535 CG2 VAL A 87 9.016 -0.292 21.483 1.00 0.79 C

ATOM 536 N SER A 88 5.925 -1.833 20.215 1.00 0.82 N

ATOM 537 CA SER A 88 5.777 -3.277 20.054 1.00 0.82 C

ATOM 538 C SER A 88 5.571 -3.598 18.579 1.00 0.82 C

ATOM 539 O SER A 88 6.148 -4.557 18.072 1.00 0.82 O

ATOM 540 CB SER A 88 4.587 -3.800 20.851 1.00 0.82 C

ATOM 541 OG SER A 88 4.871 -3.735 22.249 1.00 0.82 O

ATOM 542 N TYR A 89 4.827 -2.728 17.895 1.00 0.78 N

ATOM 543 CA TYR A 89 4.546 -2.910 16.464 1.00 0.78 C

ATOM 544 C TYR A 89 5.826 -2.736 15.638 1.00 0.78 C

ATOM 545 O TYR A 89 6.124 -3.459 14.698 1.00 0.78 O

ATOM 546 CB TYR A 89 3.479 -1.912 16.005 1.00 0.78 C

ATOM 547 CG TYR A 89 3.081 -2.098 14.534 1.00 0.78 C

ATOM 548 CD1 TYR A 89 2.095 -3.012 14.193 1.00 0.78 C

ATOM 549 CD2 TYR A 89 3.672 -1.331 13.541 1.00 0.78 C

ATOM 550 CE1 TYR A 89 1.747 -3.222 12.864 1.00 0.78 C

ATOM 551 CE2 TYR A 89 3.297 -1.503 12.213 1.00 0.78 C

ATOM 552 CZ TYR A 89 2.355 -2.465 11.874 1.00 0.78 C

ATOM 553 OH TYR A 89 2.019 -2.668 10.576 1.00 0.78 O

ATOM 554 N ASN A 90 6.611 -1.737 15.992 1.00 0.81 N

ATOM 555 CA ASN A 90 7.843 -1.438 15.254 1.00 0.81 C

ATOM 556 C ASN A 90 8.948 -2.467 15.517 1.00 0.81 C

ATOM 557 O ASN A 90 9.609 -2.926 14.584 1.00 0.81 O

ATOM 558 CB ASN A 90 8.308 -0.039 15.623 1.00 0.81 C

ATOM 559 CG ASN A 90 9.503 0.356 14.768 1.00 0.81 C

ATOM 560 ND2 ASN A 90 10.281 1.238 15.316 1.00 0.81 N

ATOM 561 OD1 ASN A 90 9.720 -0.102 13.658 1.00 0.81 O

ATOM 562 N LEU A 91 9.094 -2.857 16.782 1.00 0.82 N

ATOM 563 CA LEU A 91 10.071 -3.889 17.162 1.00 0.82 C

ATOM 564 C LEU A 91 9.861 -5.174 16.373 1.00 0.82 C

ATOM 565 O LEU A 91 10.817 -5.714 15.839 1.00 0.82 O

ATOM 566 CB LEU A 91 9.986 -4.229 18.651 1.00 0.82 C

ATOM 567 CG LEU A 91 10.596 -3.154 19.548 1.00 0.82 C

ATOM 568 CD1 LEU A 91 10.416 -3.518 21.011 1.00 0.82 C

ATOM 569 CD2 LEU A 91 12.098 -3.126 19.349 1.00 0.82 C

ATOM 570 N LYS A 92 8.600 -5.531 16.158 1.00 0.79 N

ATOM 571 CA LYS A 92 8.269 -6.745 15.400 1.00 0.79 C

ATOM 572 C LYS A 92 8.629 -6.603 13.918 1.00 0.79 C

ATOM 573 O LYS A 92 9.285 -7.450 13.332 1.00 0.79 O

ATOM 574 CB LYS A 92 6.811 -7.154 15.584 1.00 0.79 C

ATOM 575 CG LYS A 92 5.864 -6.152 14.961 1.00 0.79 C

ATOM 576 CD LYS A 92 4.422 -6.540 15.178 1.00 0.79 C

ATOM 577 CE LYS A 92 3.504 -5.511 14.566 1.00 0.79 C

ATOM 578 NZ LYS A 92 3.668 -5.239 13.128 1.00 0.79 N1+

ATOM 579 N LYS A 93 8.416 -5.419 13.379 1.00 0.78 N

ATOM 580 CA LYS A 93 8.714 -5.164 11.971 1.00 0.78 C

ATOM 581 C LYS A 93 10.218 -5.257 11.687 1.00 0.78 C

ATOM 582 O LYS A 93 10.645 -5.893 10.724 1.00 0.78 O

ATOM 583 CB LYS A 93 8.188 -3.784 11.632 1.00 0.78 C

ATOM 584 CG LYS A 93 8.418 -3.592 10.149 1.00 0.78 C

ATOM 585 CD LYS A 93 7.580 -4.429 9.189 1.00 0.78 C

ATOM 586 CE LYS A 93 7.850 -4.056 7.727 1.00 0.78 C

ATOM 587 NZ LYS A 93 9.242 -4.307 7.324 1.00 0.78 N1+

ATOM 588 N LEU A 94 10.990 -4.657 12.577 1.00 0.81 N

ATOM 589 CA LEU A 94 12.436 -4.620 12.400 1.00 0.81 C

ATOM 590 C LEU A 94 13.101 -5.967 12.678 1.00 0.81 C

ATOM 591 O LEU A 94 14.113 -6.310 12.068 1.00 0.81 O

ATOM 592 CB LEU A 94 12.971 -3.601 13.368 1.00 0.81 C

ATOM 593 CG LEU A 94 14.435 -3.428 13.136 1.00 0.81 C

ATOM 594 CD1 LEU A 94 14.550 -2.570 11.910 1.00 0.81 C

ATOM 595 CD2 LEU A 94 14.731 -2.426 14.176 1.00 0.81 C

ATOM 596 N ILE A 95 12.513 -6.710 13.605 1.00 0.79 N

ATOM 597 CA ILE A 95 13.026 -8.043 13.960 1.00 0.79 C

ATOM 598 C ILE A 95 12.765 -9.024 12.815 1.00 0.79 C

ATOM 599 O ILE A 95 13.645 -9.783 12.425 1.00 0.79 O

ATOM 600 CB ILE A 95 12.393 -8.625 15.236 1.00 0.79 C

ATOM 601 CG1 ILE A 95 10.889 -8.825 15.125 1.00 0.79 C

ATOM 602 CG2 ILE A 95 12.654 -7.790 16.468 1.00 0.79 C

ATOM 603 CD1 ILE A 95 10.194 -9.456 16.329 1.00 0.79 C

ATOM 604 N ASP A 96 11.561 -8.923 12.249 1.00 0.83 N

ATOM 605 CA ASP A 96 11.150 -9.746 11.105 1.00 0.83 C

ATOM 606 C ASP A 96 12.022 -9.436 9.890 1.00 0.83 C

ATOM 607 O ASP A 96 12.305 -10.312 9.081 1.00 0.83 O

ATOM 608 CB ASP A 96 9.680 -9.489 10.768 1.00 0.83 C

ATOM 609 CG ASP A 96 8.747 -9.984 11.879 1.00 0.83 C

ATOM 610 OD1 ASP A 96 8.797 -11.192 12.181 1.00 0.83 O

ATOM 611 OD2 ASP A 96 7.923 -9.179 12.352 1.00 0.83 O1-

ATOM 612 N LEU A 97 12.466 -8.188 9.820 1.00 0.79 N

ATOM 613 CA LEU A 97 13.346 -7.773 8.734 1.00 0.79 C

ATOM 614 C LEU A 97 14.828 -8.197 9.018 1.00 0.79 C

ATOM 615 O LEU A 97 15.751 -8.241 8.200 1.00 0.79 O

ATOM 616 CB LEU A 97 13.020 -6.280 8.518 1.00 0.79 C

ATOM 617 CG LEU A 97 13.482 -5.878 7.136 1.00 0.79 C

ATOM 618 CD1 LEU A 97 13.018 -4.533 6.596 1.00 0.79 C

ATOM 619 CD2 LEU A 97 14.934 -5.915 7.284 1.00 0.79 C

ATOM 620 N GLY A 98 15.073 -8.635 10.233 1.00 0.87 N

ATOM 621 CA GLY A 98 16.417 -9.087 10.632 1.00 0.87 C

ATOM 622 C GLY A 98 17.356 -7.900 10.836 1.00 0.87 C

ATOM 623 O GLY A 98 18.560 -7.990 10.594 1.00 0.87 O

ATOM 624 N PHE A 99 16.806 -6.847 11.422 1.00 0.84 N

ATOM 625 CA PHE A 99 17.630 -5.676 11.733 1.00 0.84 C

ATOM 626 C PHE A 99 17.948 -5.525 13.213 1.00 0.84 C

ATOM 627 O PHE A 99 18.885 -4.819 13.578 1.00 0.84 O

ATOM 628 CB PHE A 99 16.977 -4.410 11.220 1.00 0.84 C

ATOM 629 CG PHE A 99 16.663 -4.323 9.728 1.00 0.84 C

ATOM 630 CD1 PHE A 99 17.626 -4.858 8.903 1.00 0.84 C

ATOM 631 CD2 PHE A 99 15.676 -3.504 9.165 1.00 0.84 C

ATOM 632 CE1 PHE A 99 17.596 -4.610 7.544 1.00 0.84 C

ATOM 633 CE2 PHE A 99 15.662 -3.183 7.823 1.00 0.84 C

ATOM 634 CZ PHE A 99 16.612 -3.780 6.984 1.00 0.84 C

ATOM 635 N ILE A 100 17.124 -6.152 14.039 1.00 0.80 N

ATOM 636 CA ILE A 100 17.439 -6.295 15.467 1.00 0.80 C

ATOM 637 C ILE A 100 17.279 -7.754 15.874 1.00 0.80 C

ATOM 638 O ILE A 100 16.603 -8.524 15.195 1.00 0.80 O

ATOM 639 CB ILE A 100 16.568 -5.429 16.376 1.00 0.80 C

ATOM 640 CG1 ILE A 100 15.109 -5.844 16.348 1.00 0.80 C

ATOM 641 CG2 ILE A 100 16.755 -3.972 15.999 1.00 0.80 C

ATOM 642 CD1 ILE A 100 14.253 -5.115 17.387 1.00 0.80 C

ATOM 643 N LYS A 101 17.909 -8.087 16.984 1.00 0.79 N

ATOM 644 CA LYS A 101 17.756 -9.410 17.598 1.00 0.79 C

ATOM 645 C LYS A 101 17.467 -9.237 19.090 1.00 0.79 C

ATOM 646 O LYS A 101 17.819 -8.217 19.688 1.00 0.79 O

ATOM 647 CB LYS A 101 19.016 -10.251 17.361 1.00 0.79 C

ATOM 648 CG LYS A 101 20.262 -9.640 18.004 1.00 0.79 C

ATOM 649 CD LYS A 101 21.488 -10.500 17.713 1.00 0.79 C

ATOM 650 CE LYS A 101 22.742 -9.922 18.370 1.00 0.79 C

ATOM 651 NZ LYS A 101 22.638 -9.929 19.838 1.00 0.79 N1+

ATOM 652 N HIS A 102 16.820 -10.244 19.653 1.00 0.74 N

ATOM 653 CA HIS A 102 16.539 -10.260 21.096 1.00 0.74 C

ATOM 654 C HIS A 102 17.486 -11.267 21.755 1.00 0.74 C

ATOM 655 O HIS A 102 17.702 -12.366 21.245 1.00 0.74 O

ATOM 656 CB HIS A 102 15.079 -10.659 21.336 1.00 0.74 C

ATOM 657 CG HIS A 102 14.791 -12.093 20.875 1.00 0.74 C

ATOM 658 CD2 HIS A 102 14.212 -12.453 19.734 1.00 0.74 C

ATOM 659 ND1 HIS A 102 15.056 -13.194 21.572 1.00 0.74 N

ATOM 660 CE1 HIS A 102 14.643 -14.238 20.864 1.00 0.74 C

ATOM 661 NE2 HIS A 102 14.115 -13.779 19.733 1.00 0.74 N

ATOM 662 N GLN A 103 18.034 -10.861 22.885 1.00 0.70 N

ATOM 663 CA GLN A 103 18.917 -11.728 23.679 1.00 0.70 C

ATOM 664 C GLN A 103 18.257 -11.978 25.034 1.00 0.70 C

ATOM 665 O GLN A 103 17.468 -11.158 25.507 1.00 0.70 O

ATOM 666 CB GLN A 103 20.275 -11.043 23.853 1.00 0.70 C

ATOM 667 CG GLN A 103 21.291 -11.926 24.589 1.00 0.70 C

ATOM 668 CD GLN A 103 21.569 -13.225 23.831 1.00 0.70 C

ATOM 669 NE2 GLN A 103 22.495 -13.132 22.898 1.00 0.70 N

ATOM 670 OE1 GLN A 103 20.968 -14.265 24.075 1.00 0.70 O

ATOM 671 N ARG A 104 18.554 -13.140 25.606 1.00 0.57 N

ATOM 672 CA ARG A 104 17.997 -13.504 26.918 1.00 0.57 C

ATOM 673 C ARG A 104 18.471 -12.527 28.005 1.00 0.57 C

ATOM 674 O ARG A 104 19.584 -12.002 27.959 1.00 0.57 O

ATOM 675 CB ARG A 104 18.368 -14.946 27.276 1.00 0.57 C

ATOM 676 CG ARG A 104 19.877 -15.161 27.405 1.00 0.57 C

ATOM 677 CD ARG A 104 20.163 -16.591 27.853 1.00 0.57 C

ATOM 678 NE ARG A 104 21.616 -16.791 27.980 1.00 0.57 N

ATOM 679 CZ ARG A 104 22.458 -17.063 26.977 1.00 0.57 C

ATOM 680 NH1 ARG A 104 22.015 -17.174 25.727 1.00 0.57 N1+

ATOM 681 NH2 ARG A 104 23.750 -17.253 27.226 1.00 0.57 N

ATOM 682 N SER A 105 17.586 -12.316 28.966 1.00 0.59 N

ATOM 683 CA SER A 105 17.863 -11.465 30.134 1.00 0.59 C

ATOM 684 C SER A 105 17.768 -12.358 31.366 1.00 0.59 C

ATOM 685 O SER A 105 16.681 -12.628 31.875 1.00 0.59 O

ATOM 686 CB SER A 105 16.832 -10.338 30.234 1.00 0.59 C

ATOM 687 OG SER A 105 16.903 -9.518 29.070 1.00 0.59 O

ATOM 688 N ARG A 106 18.922 -12.851 31.803 1.00 0.50 N

ATOM 689 CA ARG A 106 18.962 -13.835 32.903 1.00 0.50 C

ATOM 690 C ARG A 106 18.590 -13.244 34.271 1.00 0.50 C

ATOM 691 O ARG A 106 18.304 -13.978 35.211 1.00 0.50 O

ATOM 692 CB ARG A 106 20.300 -14.579 32.954 1.00 0.50 C

ATOM 693 CG ARG A 106 21.503 -13.674 33.213 1.00 0.50 C

ATOM 694 CD ARG A 106 22.759 -14.533 33.342 1.00 0.50 C

ATOM 695 NE ARG A 106 23.932 -13.679 33.592 1.00 0.50 N

ATOM 696 CZ ARG A 106 24.632 -13.025 32.660 1.00 0.50 C

ATOM 697 NH1 ARG A 106 24.298 -13.108 31.375 1.00 0.50 N1+

ATOM 698 NH2 ARG A 106 25.682 -12.291 33.013 1.00 0.50 N

ATOM 699 N ILE A 107 18.657 -11.919 34.375 1.00 0.50 N

ATOM 700 CA ILE A 107 18.275 -11.215 35.614 1.00 0.50 C

ATOM 701 C ILE A 107 16.746 -11.164 35.777 1.00 0.50 C

ATOM 702 O ILE A 107 16.215 -11.360 36.864 1.00 0.50 O

ATOM 703 CB ILE A 107 18.882 -9.801 35.654 1.00 0.50 C

ATOM 704 CG1 ILE A 107 18.337 -8.912 34.524 1.00 0.50 C

ATOM 705 CG2 ILE A 107 20.417 -9.894 35.609 1.00 0.50 C

ATOM 706 CD1 ILE A 107 18.766 -7.449 34.632 1.00 0.50 C

ATOM 707 N ASP A 108 16.067 -10.853 34.673 1.00 0.56 N

ATOM 708 CA ASP A 108 14.608 -10.713 34.629 1.00 0.56 C

ATOM 709 C ASP A 108 14.171 -11.145 33.233 1.00 0.56 C

ATOM 710 O ASP A 108 14.141 -10.335 32.308 1.00 0.56 O

ATOM 711 CB ASP A 108 14.238 -9.253 34.926 1.00 0.56 C

ATOM 712 CG ASP A 108 12.724 -9.073 35.051 1.00 0.56 C

ATOM 713 OD1 ASP A 108 12.145 -9.610 36.010 1.00 0.56 O

ATOM 714 OD2 ASP A 108 12.152 -8.417 34.174 1.00 0.56 O1-

ATOM 715 N LYS A 109 13.788 -12.414 33.135 1.00 0.56 N

ATOM 716 CA LYS A 109 13.517 -13.055 31.831 1.00 0.56 C

ATOM 717 C LYS A 109 12.448 -12.301 31.027 1.00 0.56 C

ATOM 718 O LYS A 109 12.505 -12.217 29.804 1.00 0.56 O

ATOM 719 CB LYS A 109 13.079 -14.510 32.041 1.00 0.56 C

ATOM 720 CG LYS A 109 11.760 -14.634 32.815 1.00 0.56 C

ATOM 721 CD LYS A 109 11.367 -16.094 33.002 1.00 0.56 C

ATOM 722 CE LYS A 109 10.028 -16.225 33.733 1.00 0.56 C

ATOM 723 NZ LYS A 109 8.917 -15.677 32.940 1.00 0.56 N1+

ATOM 724 N ARG A 110 11.511 -11.734 31.783 1.00 0.56 N

ATOM 725 CA ARG A 110 10.365 -10.979 31.264 1.00 0.56 C

ATOM 726 C ARG A 110 10.808 -9.709 30.520 1.00 0.56 C

ATOM 727 O ARG A 110 10.156 -9.282 29.569 1.00 0.56 O

ATOM 728 CB ARG A 110 9.492 -10.660 32.475 1.00 0.56 C

ATOM 729 CG ARG A 110 8.031 -10.420 32.090 1.00 0.56 C

ATOM 730 CD ARG A 110 7.101 -10.830 33.235 1.00 0.56 C

ATOM 731 NE ARG A 110 7.274 -9.977 34.426 1.00 0.56 N

ATOM 732 CZ ARG A 110 7.776 -10.365 35.604 1.00 0.56 C

ATOM 733 NH1 ARG A 110 8.255 -11.594 35.783 1.00 0.56 N1+

ATOM 734 NH2 ARG A 110 7.707 -9.547 36.649 1.00 0.56 N

ATOM 735 N SER A 111 11.959 -9.168 30.913 1.00 0.66 N

ATOM 736 CA SER A 111 12.500 -7.933 30.324 1.00 0.66 C

ATOM 737 C SER A 111 13.400 -8.253 29.131 1.00 0.66 C

ATOM 738 O SER A 111 14.627 -8.242 29.227 1.00 0.66 O

ATOM 739 CB SER A 111 13.315 -7.152 31.357 1.00 0.66 C

ATOM 740 OG SER A 111 12.450 -6.649 32.363 1.00 0.66 O

ATOM 741 N ILE A 112 12.765 -8.451 27.984 1.00 0.65 N

ATOM 742 CA ILE A 112 13.489 -8.743 26.730 1.00 0.65 C

ATOM 743 C ILE A 112 14.384 -7.543 26.374 1.00 0.65 C

ATOM 744 O ILE A 112 13.950 -6.391 26.447 1.00 0.65 O

ATOM 745 CB ILE A 112 12.494 -9.028 25.591 1.00 0.65 C

ATOM 746 CG1 ILE A 112 11.648 -7.785 25.256 1.00 0.65 C

ATOM 747 CG2 ILE A 112 11.606 -10.230 25.960 1.00 0.65 C

ATOM 748 CD1 ILE A 112 10.744 -7.959 24.037 1.00 0.65 C

ATOM 749 N ARG A 113 15.616 -7.843 25.994 1.00 0.70 N

ATOM 750 CA ARG A 113 16.561 -6.795 25.574 1.00 0.70 C

ATOM 751 C ARG A 113 16.948 -7.001 24.112 1.00 0.70 C

ATOM 752 O ARG A 113 17.154 -8.129 23.659 1.00 0.70 O

ATOM 753 CB ARG A 113 17.804 -6.786 26.464 1.00 0.70 C

ATOM 754 CG ARG A 113 17.456 -6.346 27.886 1.00 0.70 C

ATOM 755 CD ARG A 113 18.718 -6.165 28.728 1.00 0.70 C

ATOM 756 NE ARG A 113 18.351 -5.678 30.071 1.00 0.70 N

ATOM 757 CZ ARG A 113 18.113 -4.407 30.410 1.00 0.70 C

ATOM 758 NH1 ARG A 113 18.202 -3.430 29.512 1.00 0.70 N1+

ATOM 759 NH2 ARG A 113 17.777 -4.106 31.660 1.00 0.70 N

ATOM 760 N ILE A 114 17.087 -5.883 23.418 1.00 0.77 N

ATOM 761 CA ILE A 114 17.271 -5.887 21.955 1.00 0.77 C

ATOM 762 C ILE A 114 18.586 -5.208 21.570 1.00 0.77 C

ATOM 763 O ILE A 114 19.037 -4.302 22.266 1.00 0.77 O

ATOM 764 CB ILE A 114 16.096 -5.173 21.277 1.00 0.77 C

ATOM 765 CG1 ILE A 114 16.064 -3.702 21.704 1.00 0.77 C

ATOM 766 CG2 ILE A 114 14.775 -5.899 21.593 1.00 0.77 C

ATOM 767 CD1 ILE A 114 15.049 -2.942 20.887 1.00 0.77 C

ATOM 768 N SER A 115 19.156 -5.687 20.476 1.00 0.82 N

ATOM 769 CA SER A 115 20.381 -5.122 19.888 1.00 0.82 C

ATOM 770 C SER A 115 20.253 -5.100 18.365 1.00 0.82 C

ATOM 771 O SER A 115 19.594 -5.965 17.785 1.00 0.82 O

ATOM 772 CB SER A 115 21.578 -5.985 20.290 1.00 0.82 C

ATOM 773 OG SER A 115 22.772 -5.446 19.723 1.00 0.82 O

ATOM 774 N LEU A 116 20.849 -4.084 17.752 1.00 0.83 N

ATOM 775 CA LEU A 116 20.961 -4.020 16.286 1.00 0.83 C

ATOM 776 C LEU A 116 21.779 -5.206 15.777 1.00 0.83 C

ATOM 777 O LEU A 116 22.727 -5.649 16.423 1.00 0.83 O

ATOM 778 CB LEU A 116 21.711 -2.754 15.868 1.00 0.83 C

ATOM 779 CG LEU A 116 20.910 -1.484 16.102 1.00 0.83 C

ATOM 780 CD1 LEU A 116 21.784 -0.245 15.917 1.00 0.83 C

ATOM 781 CD2 LEU A 116 19.764 -1.467 15.093 1.00 0.83 C

ATOM 782 N THR A 117 21.333 -5.765 14.665 1.00 0.91 N

ATOM 783 CA THR A 117 22.159 -6.716 13.900 1.00 0.91 C

ATOM 784 C THR A 117 23.103 -5.873 13.036 1.00 0.91 C

ATOM 785 O THR A 117 22.900 -4.670 12.861 1.00 0.91 O

ATOM 786 CB THR A 117 21.285 -7.611 13.017 1.00 0.91 C

ATOM 787 CG2 THR A 117 20.296 -8.428 13.856 1.00 0.91 C

ATOM 788 OG1 THR A 117 20.601 -6.792 12.069 1.00 0.91 O

ATOM 789 N GLN A 118 24.150 -6.484 12.504 1.00 0.79 N

ATOM 790 CA GLN A 118 25.077 -5.787 11.587 1.00 0.79 C

ATOM 791 C GLN A 118 24.323 -5.080 10.449 1.00 0.79 C

ATOM 792 O GLN A 118 24.667 -3.960 10.082 1.00 0.79 O

ATOM 793 CB GLN A 118 26.129 -6.727 10.981 1.00 0.79 C

ATOM 794 CG GLN A 118 25.565 -7.786 10.020 1.00 0.79 C

ATOM 795 CD GLN A 118 24.775 -8.896 10.721 1.00 0.79 C

ATOM 796 NE2 GLN A 118 24.275 -9.811 9.926 1.00 0.79 N

ATOM 797 OE1 GLN A 118 24.651 -8.991 11.935 1.00 0.79 O

ATOM 798 N SER A 119 23.264 -5.720 9.948 1.00 0.86 N

ATOM 799 CA SER A 119 22.439 -5.136 8.874 1.00 0.86 C

ATOM 800 C SER A 119 21.674 -3.900 9.360 1.00 0.86 C

ATOM 801 O SER A 119 21.677 -2.866 8.692 1.00 0.86 O

ATOM 802 CB SER A 119 21.448 -6.159 8.315 1.00 0.86 C

ATOM 803 OG SER A 119 20.564 -6.586 9.349 1.00 0.86 O

ATOM 804 N GLY A 120 21.121 -4.005 10.577 1.00 0.93 N

ATOM 805 CA GLY A 120 20.404 -2.884 11.214 1.00 0.93 C

ATOM 806 C GLY A 120 21.366 -1.718 11.471 1.00 0.93 C

ATOM 807 O GLY A 120 21.041 -0.554 11.230 1.00 0.93 O

ATOM 808 N LYS A 121 22.584 -2.087 11.858 1.00 0.81 N

ATOM 809 CA LYS A 121 23.676 -1.135 12.106 1.00 0.81 C

ATOM 810 C LYS A 121 24.056 -0.382 10.826 1.00 0.81 C

ATOM 811 O LYS A 121 24.218 0.838 10.846 1.00 0.81 O

ATOM 812 CB LYS A 121 24.894 -1.896 12.640 1.00 0.81 C

ATOM 813 CG LYS A 121 26.055 -0.967 12.998 1.00 0.81 C

ATOM 814 CD LYS A 121 25.687 -0.035 14.156 1.00 0.81 C

ATOM 815 CE LYS A 121 26.845 0.897 14.506 1.00 0.81 C

ATOM 816 NZ LYS A 121 27.167 1.798 13.390 1.00 0.81 N1+

ATOM 817 N GLU A 122 24.147 -1.129 9.728 1.00 0.83 N

ATOM 818 CA GLU A 122 24.502 -0.568 8.418 1.00 0.83 C

ATOM 819 C GLU A 122 23.419 0.411 7.938 1.00 0.83 C

ATOM 820 O GLU A 122 23.691 1.455 7.362 1.00 0.83 O

ATOM 821 CB GLU A 122 24.742 -1.709 7.424 1.00 0.83 C

ATOM 822 CG GLU A 122 25.142 -1.218 6.022 1.00 0.83 C

ATOM 823 CD GLU A 122 23.977 -0.583 5.244 1.00 0.83 C

ATOM 824 OE1 GLU A 122 22.827 -1.016 5.474 1.00 0.83 O

ATOM 825 OE2 GLU A 122 24.262 0.315 4.423 1.00 0.83 O1-

ATOM 826 N ILE A 123 22.170 0.049 8.138 1.00 0.82 N

ATOM 827 CA ILE A 123 21.048 0.884 7.680 1.00 0.82 C

ATOM 828 C ILE A 123 20.996 2.219 8.424 1.00 0.82 C

ATOM 829 O ILE A 123 20.942 3.276 7.798 1.00 0.82 O

ATOM 830 CB ILE A 123 19.748 0.092 7.822 1.00 0.82 C

ATOM 831 CG1 ILE A 123 19.803 -1.021 6.779 1.00 0.82 C

ATOM 832 CG2 ILE A 123 18.481 0.979 7.849 1.00 0.82 C

ATOM 833 CD1 ILE A 123 18.514 -1.767 6.709 1.00 0.82 C

ATOM 834 N ALA A 124 20.951 2.143 9.752 1.00 0.89 N

ATOM 835 CA ALA A 124 20.852 3.356 10.583 1.00 0.89 C

ATOM 836 C ALA A 124 22.057 4.277 10.370 1.00 0.89 C

ATOM 837 O ALA A 124 21.910 5.496 10.309 1.00 0.89 O

ATOM 838 CB ALA A 124 20.753 2.976 12.060 1.00 0.89 C

ATOM 839 N GLU A 125 23.230 3.673 10.177 1.00 0.85 N

ATOM 840 CA GLU A 125 24.455 4.447 9.910 1.00 0.85 C

ATOM 841 C GLU A 125 24.345 5.203 8.580 1.00 0.85 C

ATOM 842 O GLU A 125 24.680 6.380 8.512 1.00 0.85 O

ATOM 843 CB GLU A 125 25.701 3.554 9.936 1.00 0.85 C

ATOM 844 CG GLU A 125 25.725 2.581 8.769 1.00 0.85 C

ATOM 845 CD GLU A 125 26.875 1.604 8.715 1.00 0.85 C

ATOM 846 OE1 GLU A 125 27.104 0.943 9.748 1.00 0.85 O

ATOM 847 OE2 GLU A 125 27.490 1.564 7.631 1.00 0.85 O1-

ATOM 848 N THR A 126 23.804 4.528 7.567 1.00 0.93 N

ATOM 849 CA THR A 126 23.627 5.112 6.227 1.00 0.93 C

ATOM 850 C THR A 126 22.606 6.243 6.276 1.00 0.93 C

ATOM 851 O THR A 126 22.803 7.311 5.697 1.00 0.93 O

ATOM 852 CB THR A 126 23.174 4.045 5.224 1.00 0.93 C

ATOM 853 CG2 THR A 126 22.837 4.636 3.850 1.00 0.93 C

ATOM 854 OG1 THR A 126 24.219 3.081 5.090 1.00 0.93 O

ATOM 855 N ILE A 127 21.540 5.991 7.010 1.00 0.83 N

ATOM 856 CA ILE A 127 20.491 6.981 7.121 1.00 0.83 C

ATOM 857 C ILE A 127 21.043 8.277 7.773 1.00 0.83 C

ATOM 858 O ILE A 127 20.721 9.388 7.343 1.00 0.83 O

ATOM 859 CB ILE A 127 19.375 6.293 7.883 1.00 0.83 C

ATOM 860 CG1 ILE A 127 18.594 5.236 7.081 1.00 0.83 C

ATOM 861 CG2 ILE A 127 18.510 7.477 8.064 1.00 0.83 C

ATOM 862 CD1 ILE A 127 17.484 4.530 7.879 1.00 0.83 C

ATOM 863 N SER A 128 21.710 8.074 8.899 1.00 0.91 N

ATOM 864 CA SER A 128 22.243 9.193 9.689 1.00 0.91 C

ATOM 865 C SER A 128 23.257 10.010 8.887 1.00 0.91 C

ATOM 866 O SER A 128 23.226 11.239 8.926 1.00 0.91 O

ATOM 867 CB SER A 128 22.908 8.680 10.961 1.00 0.91 C

ATOM 868 OG SER A 128 21.921 8.064 11.790 1.00 0.91 O

ATOM 869 N GLN A 129 24.072 9.320 8.089 1.00 0.87 N

ATOM 870 CA GLN A 129 25.086 9.993 7.255 1.00 0.87 C

ATOM 871 C GLN A 129 24.425 10.877 6.193 1.00 0.87 C

ATOM 872 O GLN A 129 24.850 12.004 5.945 1.00 0.87 O

ATOM 873 CB GLN A 129 25.994 8.982 6.560 1.00 0.87 C

ATOM 874 CG GLN A 129 26.829 8.191 7.566 1.00 0.87 C

ATOM 875 CD GLN A 129 27.757 7.210 6.853 1.00 0.87 C

ATOM 876 NE2 GLN A 129 27.655 5.946 7.218 1.00 0.87 N

ATOM 877 OE1 GLN A 129 28.580 7.593 6.033 1.00 0.87 O

ATOM 878 N LEU A 130 23.327 10.370 5.643 1.00 0.86 N

ATOM 879 CA LEU A 130 22.571 11.092 4.614 1.00 0.86 C

ATOM 880 C LEU A 130 21.837 12.300 5.222 1.00 0.86 C

ATOM 881 O LEU A 130 21.776 13.393 4.668 1.00 0.86 O

ATOM 882 CB LEU A 130 21.643 10.099 3.907 1.00 0.86 C

ATOM 883 CG LEU A 130 20.885 10.737 2.746 1.00 0.86 C

ATOM 884 CD1 LEU A 130 20.420 9.672 1.756 1.00 0.86 C

ATOM 885 CD2 LEU A 130 19.624 11.349 3.335 1.00 0.86 C

ATOM 886 N TYR A 131 21.246 12.099 6.388 1.00 0.83 N

ATOM 887 CA TYR A 131 20.517 13.177 7.076 1.00 0.83 C

ATOM 888 C TYR A 131 21.458 14.319 7.475 1.00 0.83 C

ATOM 889 O TYR A 131 21.143 15.490 7.271 1.00 0.83 O

ATOM 890 CB TYR A 131 19.819 12.615 8.313 1.00 0.83 C

ATOM 891 CG TYR A 131 19.043 13.704 9.060 1.00 0.83 C

ATOM 892 CD1 TYR A 131 17.774 14.065 8.630 1.00 0.83 C

ATOM 893 CD2 TYR A 131 19.588 14.291 10.195 1.00 0.83 C

ATOM 894 CE1 TYR A 131 17.041 15.004 9.337 1.00 0.83 C

ATOM 895 CE2 TYR A 131 18.857 15.231 10.906 1.00 0.83 C

ATOM 896 CZ TYR A 131 17.588 15.583 10.471 1.00 0.83 C

ATOM 897 OH TYR A 131 16.867 16.490 11.161 1.00 0.83 O

ATOM 898 N GLN A 132 22.624 13.953 8.001 1.00 0.80 N

ATOM 899 CA GLN A 132 23.632 14.939 8.410 1.00 0.80 C

ATOM 900 C GLN A 132 24.237 15.698 7.220 1.00 0.80 C

ATOM 901 O GLN A 132 24.610 16.866 7.279 1.00 0.80 O

ATOM 902 CB GLN A 132 24.726 14.312 9.273 1.00 0.80 C

ATOM 903 CG GLN A 132 25.562 13.308 8.489 1.00 0.80 C

ATOM 904 CD GLN A 132 26.642 12.683 9.349 1.00 0.80 C

ATOM 905 NE2 GLN A 132 27.873 12.862 8.916 1.00 0.80 N

ATOM 906 OE1 GLN A 132 26.378 12.091 10.384 1.00 0.80 O

ATOM 907 N ARG A 133 24.381 15.008 6.105 1.00 0.78 N

ATOM 908 CA ARG A 133 24.917 15.659 4.900 1.00 0.78 C

ATOM 909 C ARG A 133 23.910 16.677 4.348 1.00 0.78 C

ATOM 910 O ARG A 133 24.292 17.765 3.924 1.00 0.78 O

ATOM 911 CB ARG A 133 25.313 14.634 3.841 1.00 0.78 C

ATOM 912 CG ARG A 133 24.117 13.862 3.305 1.00 0.78 C

ATOM 913 CD ARG A 133 24.450 12.838 2.243 1.00 0.78 C

ATOM 914 NE ARG A 133 25.382 11.840 2.787 1.00 0.78 N

ATOM 915 CZ ARG A 133 25.969 10.900 2.049 1.00 0.78 C

ATOM 916 NH1 ARG A 133 25.734 10.825 0.742 1.00 0.78 N1+

ATOM 917 NH2 ARG A 133 26.789 10.026 2.616 1.00 0.78 N

ATOM 918 N HIS A 134 22.627 16.351 4.498 1.00 0.79 N

ATOM 919 CA HIS A 134 21.542 17.260 4.095 1.00 0.79 C

ATOM 920 C HIS A 134 21.456 18.462 5.025 1.00 0.79 C

ATOM 921 O HIS A 134 21.243 19.586 4.576 1.00 0.79 O

ATOM 922 CB HIS A 134 20.191 16.545 4.069 1.00 0.79 C

ATOM 923 CG HIS A 134 20.109 15.483 2.970 1.00 0.79 C

ATOM 924 CD2 HIS A 134 20.917 15.353 1.917 1.00 0.79 C

ATOM 925 ND1 HIS A 134 19.151 14.572 2.864 1.00 0.79 N

ATOM 926 CE1 HIS A 134 19.350 13.940 1.709 1.00 0.79 C

ATOM 927 NE2 HIS A 134 20.470 14.373 1.151 1.00 0.79 N

ATOM 928 N ILE A 135 21.694 18.209 6.308 1.00 0.75 N

ATOM 929 CA ILE A 135 21.673 19.282 7.304 1.00 0.75 C

ATOM 930 C ILE A 135 22.844 20.217 7.053 1.00 0.75 C

ATOM 931 O ILE A 135 22.709 21.383 7.290 1.00 0.75 O

ATOM 932 CB ILE A 135 21.684 18.796 8.752 1.00 0.75 C

ATOM 933 CG1 ILE A 135 22.991 18.105 9.091 1.00 0.75 C

ATOM 934 CG2 ILE A 135 20.457 17.920 9.029 1.00 0.75 C

ATOM 935 CD1 ILE A 135 22.979 17.419 10.450 1.00 0.75 C

ATOM 936 N GLU A 136 23.973 19.712 6.603 1.00 0.73 N

ATOM 937 CA GLU A 136 25.149 20.536 6.308 1.00 0.73 C

ATOM 938 C GLU A 136 24.869 21.428 5.091 1.00 0.73 C

ATOM 939 O GLU A 136 25.299 22.578 5.060 1.00 0.73 O

ATOM 940 CB GLU A 136 26.342 19.600 6.107 1.00 0.73 C

ATOM 941 CG GLU A 136 27.642 20.348 5.792 1.00 0.73 C

ATOM 942 CD GLU A 136 27.647 20.911 4.367 1.00 0.73 C

ATOM 943 OE1 GLU A 136 27.030 20.306 3.469 1.00 0.73 O

ATOM 944 OE2 GLU A 136 28.334 21.916 4.180 1.00 0.73 O1-

ATOM 945 N SER A 137 24.168 20.865 4.110 1.00 0.80 N

ATOM 946 CA SER A 137 23.786 21.614 2.899 1.00 0.80 C

ATOM 947 C SER A 137 22.879 22.790 3.266 1.00 0.80 C

ATOM 948 O SER A 137 23.048 23.899 2.766 1.00 0.80 O

ATOM 949 CB SER A 137 23.047 20.710 1.912 1.00 0.80 C

ATOM 950 OG SER A 137 23.913 19.659 1.481 1.00 0.80 O

ATOM 951 N ILE A 138 21.964 22.532 4.198 1.00 0.73 N

ATOM 952 CA ILE A 138 21.042 23.564 4.692 1.00 0.73 C

ATOM 953 C ILE A 138 21.681 24.435 5.784 1.00 0.73 C

ATOM 954 O ILE A 138 21.562 25.654 5.816 1.00 0.73 O

ATOM 955 CB ILE A 138 19.728 22.929 5.183 1.00 0.73 C

ATOM 956 CG1 ILE A 138 18.723 24.002 5.613 1.00 0.73 C

ATOM 957 CG2 ILE A 138 19.914 21.944 6.338 1.00 0.73 C

ATOM 958 CD1 ILE A 138 18.390 24.970 4.473 1.00 0.73 C

ATOM 959 N ASP A 139 22.347 23.764 6.695 1.00 0.68 N

ATOM 960 CA ASP A 139 23.021 24.342 7.849 1.00 0.68 C

ATOM 961 C ASP A 139 24.521 24.268 7.562 1.00 0.68 C

ATOM 962 O ASP A 139 25.248 23.417 8.078 1.00 0.68 O

ATOM 963 CB ASP A 139 22.639 23.583 9.132 1.00 0.68 C

ATOM 964 CG ASP A 139 23.271 24.220 10.365 1.00 0.68 C

ATOM 965 OD1 ASP A 139 24.384 24.765 10.238 1.00 0.68 O

ATOM 966 OD2 ASP A 139 22.676 24.102 11.449 1.00 0.68 O1-

ATOM 967 N LYS A 140 24.955 25.264 6.806 1.00 0.60 N

ATOM 968 CA LYS A 140 26.368 25.398 6.440 1.00 0.60 C

ATOM 969 C LYS A 140 26.987 26.483 7.314 1.00 0.60 C

ATOM 970 O LYS A 140 26.379 27.511 7.542 1.00 0.60 O

ATOM 971 CB LYS A 140 26.453 25.784 4.960 1.00 0.60 C

ATOM 972 CG LYS A 140 27.891 25.913 4.453 1.00 0.60 C

ATOM 973 CD LYS A 140 28.620 24.585 4.578 1.00 0.60 C

ATOM 974 CE LYS A 140 30.051 24.599 4.055 1.00 0.60 C

ATOM 975 NZ LYS A 140 30.851 25.479 4.904 1.00 0.60 N1+

ATOM 976 N VAL A 141 28.232 26.274 7.713 1.00 0.58 N

ATOM 977 CA VAL A 141 28.977 27.262 8.521 1.00 0.58 C

ATOM 978 C VAL A 141 29.121 28.596 7.767 1.00 0.58 C

ATOM 979 O VAL A 141 29.128 29.665 8.376 1.00 0.58 O

ATOM 980 CB VAL A 141 30.351 26.690 8.907 1.00 0.58 C

ATOM 981 CG1 VAL A 141 31.240 26.399 7.698 1.00 0.58 C

ATOM 982 CG2 VAL A 141 31.090 27.629 9.865 1.00 0.58 C

ATOM 983 N GLY A 142 29.308 28.461 6.446 1.00 0.59 N

ATOM 984 CA GLY A 142 29.471 29.624 5.562 1.00 0.59 C

ATOM 985 C GLY A 142 28.229 30.515 5.639 1.00 0.59 C

ATOM 986 O GLY A 142 28.322 31.740 5.662 1.00 0.59 O

ATOM 987 N GLY A 143 27.076 29.851 5.679 1.00 0.61 N

ATOM 988 CA GLY A 143 25.814 30.581 5.493 1.00 0.61 C

ATOM 989 C GLY A 143 24.648 29.592 5.355 1.00 0.61 C

ATOM 990 O GLY A 143 24.792 28.692 4.546 1.00 0.61 O

ATOM 991 N LEU A 144 23.552 29.685 6.110 1.00 0.58 N

ATOM 992 CA LEU A 144 23.443 30.590 7.259 1.00 0.58 C

ATOM 993 C LEU A 144 24.674 30.592 8.171 1.00 0.58 C

ATOM 994 O LEU A 144 25.483 29.681 8.159 1.00 0.58 O

ATOM 995 CB LEU A 144 22.267 30.134 8.089 1.00 0.58 C

ATOM 996 CG LEU A 144 22.108 30.911 9.356 1.00 0.58 C

ATOM 997 CD1 LEU A 144 21.639 32.212 8.743 1.00 0.58 C

ATOM 998 CD2 LEU A 144 20.991 30.371 10.208 1.00 0.58 C

ATOM 999 N SER A 145 24.946 31.737 8.763 1.00 0.63 N

ATOM 1000 CA SER A 145 26.124 31.841 9.635 1.00 0.63 C

ATOM 1001 C SER A 145 25.920 30.890 10.822 1.00 0.63 C

ATOM 1002 O SER A 145 24.788 30.569 11.199 1.00 0.63 O

ATOM 1003 CB SER A 145 26.281 33.281 10.114 1.00 0.63 C

ATOM 1004 OG SER A 145 25.185 33.625 10.970 1.00 0.63 O

ATOM 1005 N VAL A 146 27.025 30.509 11.450 1.00 0.70 N

ATOM 1006 CA VAL A 146 26.952 29.653 12.647 1.00 0.70 C

ATOM 1007 C VAL A 146 26.205 30.380 13.774 1.00 0.70 C

ATOM 1008 O VAL A 146 25.487 29.756 14.541 1.00 0.70 O

ATOM 1009 CB VAL A 146 28.353 29.217 13.104 1.00 0.70 C

ATOM 1010 CG1 VAL A 146 29.237 30.410 13.493 1.00 0.70 C

ATOM 1011 CG2 VAL A 146 28.261 28.203 14.250 1.00 0.70 C

ATOM 1012 N ASP A 147 26.366 31.700 13.828 1.00 0.70 N

ATOM 1013 CA ASP A 147 25.690 32.531 14.831 1.00 0.70 C

ATOM 1014 C ASP A 147 24.169 32.451 14.680 1.00 0.70 C

ATOM 1015 O ASP A 147 23.405 32.407 15.637 1.00 0.70 O

ATOM 1016 CB ASP A 147 26.159 33.982 14.748 1.00 0.70 C

ATOM 1017 CG ASP A 147 25.505 34.831 15.843 1.00 0.70 C

ATOM 1018 OD1 ASP A 147 25.828 34.585 17.022 1.00 0.70 O

ATOM 1019 OD2 ASP A 147 24.694 35.699 15.460 1.00 0.70 O1-

ATOM 1020 N ASP A 148 23.711 32.469 13.448 1.00 0.73 N

ATOM 1021 CA ASP A 148 22.265 32.385 13.270 1.00 0.73 C

ATOM 1022 C ASP A 148 21.711 30.995 13.611 1.00 0.73 C

ATOM 1023 O ASP A 148 20.654 30.896 14.231 1.00 0.73 O

ATOM 1024 CB ASP A 148 21.923 32.897 11.894 1.00 0.73 C

ATOM 1025 CG ASP A 148 20.404 32.838 11.584 1.00 0.73 C

ATOM 1026 OD1 ASP A 148 19.683 32.003 12.167 1.00 0.73 O

ATOM 1027 OD2 ASP A 148 19.992 33.458 10.587 1.00 0.73 O1-

ATOM 1028 N PHE A 149 22.486 29.960 13.311 1.00 0.69 N

ATOM 1029 CA PHE A 149 22.096 28.584 13.658 1.00 0.69 C

ATOM 1030 C PHE A 149 22.110 28.341 15.162 1.00 0.69 C

ATOM 1031 O PHE A 149 21.244 27.659 15.706 1.00 0.69 O

ATOM 1032 CB PHE A 149 23.017 27.570 12.978 1.00 0.69 C

ATOM 1033 CG PHE A 149 22.858 27.522 11.456 1.00 0.69 C

ATOM 1034 CD1 PHE A 149 21.623 27.648 10.881 1.00 0.69 C

ATOM 1035 CD2 PHE A 149 23.929 27.305 10.629 1.00 0.69 C

ATOM 1036 CE1 PHE A 149 21.385 27.568 9.509 1.00 0.69 C

ATOM 1037 CE2 PHE A 149 23.728 27.208 9.257 1.00 0.69 C

ATOM 1038 CZ PHE A 149 22.472 27.327 8.691 1.00 0.69 C

ATOM 1039 N ILE A 150 23.082 28.960 15.821 1.00 0.72 N

ATOM 1040 CA ILE A 150 23.222 28.837 17.281 1.00 0.72 C

ATOM 1041 C ILE A 150 22.039 29.525 17.973 1.00 0.72 C

ATOM 1042 O ILE A 150 21.471 28.993 18.922 1.00 0.72 O

ATOM 1043 CB ILE A 150 24.553 29.413 17.793 1.00 0.72 C

ATOM 1044 CG1 ILE A 150 24.635 30.921 17.578 1.00 0.72 C

ATOM 1045 CG2 ILE A 150 25.744 28.667 17.184 1.00 0.72 C

ATOM 1046 CD1 ILE A 150 25.867 31.643 18.103 1.00 0.72 C

ATOM 1047 N ALA A 151 21.652 30.676 17.418 1.00 0.79 N

ATOM 1048 CA ALA A 151 20.502 31.446 17.908 1.00 0.79 C

ATOM 1049 C ALA A 151 19.220 30.636 17.741 1.00 0.79 C

ATOM 1050 O ALA A 151 18.405 30.555 18.658 1.00 0.79 O

ATOM 1051 CB ALA A 151 20.388 32.747 17.117 1.00 0.79 C

ATOM 1052 N MET A 152 19.139 29.925 16.620 1.00 0.75 N

ATOM 1053 CA MET A 152 17.976 29.081 16.370 1.00 0.75 C

ATOM 1054 C MET A 152 17.884 27.955 17.400 1.00 0.75 C

ATOM 1055 O MET A 152 16.809 27.707 17.932 1.00 0.75 O

ATOM 1056 CB MET A 152 18.098 28.458 15.013 1.00 0.75 C

ATOM 1057 CG MET A 152 16.884 27.553 14.822 1.00 0.75 C

ATOM 1058 SD MET A 152 15.366 28.468 14.386 1.00 0.75 S

ATOM 1059 CE MET A 152 14.203 27.138 14.169 1.00 0.75 C

ATOM 1060 N ASN A 153 19.022 27.334 17.677 1.00 0.75 N

ATOM 1061 CA ASN A 153 19.066 26.240 18.652 1.00 0.75 C

ATOM 1062 C ASN A 153 18.730 26.746 20.059 1.00 0.75 C

ATOM 1063 O ASN A 153 17.911 26.155 20.756 1.00 0.75 O

ATOM 1064 CB ASN A 153 20.455 25.622 18.680 1.00 0.75 C

ATOM 1065 CG ASN A 153 20.484 24.383 19.587 1.00 0.75 C

ATOM 1066 ND2 ASN A 153 21.412 23.502 19.365 1.00 0.75 N

ATOM 1067 OD1 ASN A 153 19.705 24.194 20.496 1.00 0.75 O

ATOM 1068 N LYS A 154 19.337 27.872 20.426 1.00 0.74 N

ATOM 1069 CA LYS A 154 19.151 28.480 21.754 1.00 0.74 C

ATOM 1070 C LYS A 154 17.673 28.808 21.979 1.00 0.74 C

ATOM 1071 O LYS A 154 17.082 28.388 22.962 1.00 0.74 O

ATOM 1072 CB LYS A 154 19.990 29.757 21.846 1.00 0.74 C

ATOM 1073 CG LYS A 154 19.899 30.420 23.224 1.00 0.74 C

ATOM 1074 CD LYS A 154 20.651 31.750 23.227 1.00 0.74 C

ATOM 1075 CE LYS A 154 20.523 32.466 24.574 1.00 0.74 C

ATOM 1076 NZ LYS A 154 21.155 33.794 24.526 1.00 0.74 N1+

ATOM 1077 N LEU A 155 17.074 29.466 20.998 1.00 0.78 N

ATOM 1078 CA LEU A 155 15.671 29.869 21.092 1.00 0.78 C

ATOM 1079 C LEU A 155 14.732 28.658 21.012 1.00 0.78 C

ATOM 1080 O LEU A 155 13.732 28.605 21.727 1.00 0.78 O

ATOM 1081 CB LEU A 155 15.402 30.897 20.001 1.00 0.78 C

ATOM 1082 CG LEU A 155 14.022 31.491 20.220 1.00 0.78 C

ATOM 1083 CD1 LEU A 155 13.903 32.737 19.424 1.00 0.78 C

ATOM 1084 CD2 LEU A 155 12.946 30.646 19.617 1.00 0.78 C

ATOM 1085 N LEU A 156 15.071 27.706 20.150 1.00 0.79 N

ATOM 1086 CA LEU A 156 14.259 26.498 19.986 1.00 0.79 C

ATOM 1087 C LEU A 156 14.141 25.754 21.319 1.00 0.79 C

ATOM 1088 O LEU A 156 13.028 25.440 21.725 1.00 0.79 O

ATOM 1089 CB LEU A 156 14.863 25.634 18.876 1.00 0.79 C

ATOM 1090 CG LEU A 156 14.020 24.407 18.596 1.00 0.79 C

ATOM 1091 CD1 LEU A 156 14.295 23.885 17.199 1.00 0.79 C

ATOM 1092 CD2 LEU A 156 14.485 23.361 19.555 1.00 0.79 C

ATOM 1093 N GLN A 157 15.270 25.570 21.995 1.00 0.77 N

ATOM 1094 CA GLN A 157 15.292 24.850 23.281 1.00 0.77 C

ATOM 1095 C GLN A 157 14.646 25.668 24.405 1.00 0.77 C

ATOM 1096 O GLN A 157 13.922 25.134 25.243 1.00 0.77 O

ATOM 1097 CB GLN A 157 16.718 24.440 23.654 1.00 0.77 C

ATOM 1098 CG GLN A 157 17.649 25.636 23.845 1.00 0.77 C

ATOM 1099 CD GLN A 157 19.044 25.200 24.251 1.00 0.77 C

ATOM 1100 NE2 GLN A 157 20.013 25.571 23.440 1.00 0.77 N

ATOM 1101 OE1 GLN A 157 19.242 24.561 25.273 1.00 0.77 O

ATOM 1102 N ARG A 158 14.861 26.982 24.357 1.00 0.74 N

ATOM 1103 CA ARG A 158 14.274 27.905 25.344 1.00 0.74 C

ATOM 1104 C ARG A 158 12.756 27.927 25.233 1.00 0.74 C

ATOM 1105 O ARG A 158 12.038 27.860 26.228 1.00 0.74 O

ATOM 1106 CB ARG A 158 14.811 29.318 25.159 1.00 0.74 C

ATOM 1107 CG ARG A 158 16.287 29.387 25.551 1.00 0.74 C

ATOM 1108 CD ARG A 158 16.840 30.791 25.341 1.00 0.74 C

ATOM 1109 NE ARG A 158 16.187 31.723 26.276 1.00 0.74 N

ATOM 1110 CZ ARG A 158 16.377 33.042 26.295 1.00 0.74 C

ATOM 1111 NH1 ARG A 158 17.202 33.617 25.424 1.00 0.74 N1+

ATOM 1112 NH2 ARG A 158 15.740 33.797 27.184 1.00 0.74 N

ATOM 1113 N LEU A 159 12.297 27.900 23.995 1.00 0.79 N

ATOM 1114 CA LEU A 159 10.893 27.688 23.739 1.00 0.79 C

ATOM 1115 C LEU A 159 10.200 26.491 24.266 1.00 0.79 C

ATOM 1116 O LEU A 159 9.220 26.581 24.996 1.00 0.79 O

ATOM 1117 CB LEU A 159 10.591 27.786 22.257 1.00 0.79 C

ATOM 1118 CG LEU A 159 10.489 29.210 21.868 1.00 0.79 C

ATOM 1119 CD1 LEU A 159 10.317 29.075 20.312 1.00 0.79 C

ATOM 1120 CD2 LEU A 159 9.593 29.824 23.025 1.00 0.79 C

ATOM 1121 N ASN A 160 10.901 25.412 24.039 1.00 0.77 N

ATOM 1122 CA ASN A 160 10.414 24.118 24.512 1.00 0.77 C

ATOM 1123 C ASN A 160 10.250 24.141 26.033 1.00 0.77 C

ATOM 1124 O ASN A 160 9.211 23.741 26.554 1.00 0.77 O

ATOM 1125 CB ASN A 160 11.416 23.031 24.148 1.00 0.77 C

ATOM 1126 CG ASN A 160 11.558 22.895 22.644 1.00 0.77 C

ATOM 1127 ND2 ASN A 160 10.509 22.453 22.050 1.00 0.77 N

ATOM 1128 OD1 ASN A 160 12.594 23.030 22.034 1.00 0.77 O

ATOM 1129 N ARG A 161 11.244 24.740 26.689 1.00 0.71 N

ATOM 1130 CA ARG A 161 11.255 24.926 28.150 1.00 0.71 C

ATOM 1131 C ARG A 161 10.079 25.800 28.591 1.00 0.71 C

ATOM 1132 O ARG A 161 9.321 25.459 29.500 1.00 0.71 O

ATOM 1133 CB ARG A 161 12.509 25.697 28.563 1.00 0.71 C

ATOM 1134 CG ARG A 161 13.213 25.009 29.731 1.00 0.71 C

ATOM 1135 CD ARG A 161 14.511 24.370 29.242 1.00 0.71 C

ATOM 1136 NE ARG A 161 14.226 23.582 28.029 1.00 0.71 N

ATOM 1137 CZ ARG A 161 15.123 22.984 27.252 1.00 0.71 C

ATOM 1138 NH1 ARG A 161 16.416 23.040 27.546 1.00 0.71 N1+

ATOM 1139 NH2 ARG A 161 14.717 22.351 26.160 1.00 0.71 N

ATOM 1140 N PHE A 162 9.946 26.918 27.884 1.00 0.71 N

ATOM 1141 CA PHE A 162 8.984 27.975 28.217 1.00 0.71 C

ATOM 1142 C PHE A 162 7.536 27.488 28.156 1.00 0.71 C

ATOM 1143 O PHE A 162 6.761 27.732 29.082 1.00 0.71 O

ATOM 1144 CB PHE A 162 9.205 29.154 27.269 1.00 0.71 C

ATOM 1145 CG PHE A 162 8.177 30.261 27.505 1.00 0.71 C

ATOM 1146 CD1 PHE A 162 8.319 31.131 28.578 1.00 0.71 C

ATOM 1147 CD2 PHE A 162 7.114 30.400 26.622 1.00 0.71 C

ATOM 1148 CE1 PHE A 162 7.395 32.150 28.767 1.00 0.71 C

ATOM 1149 CE2 PHE A 162 6.191 31.420 26.810 1.00 0.71 C

ATOM 1150 CZ PHE A 162 6.332 32.295 27.881 1.00 0.71 C

ATOM 1151 N TRP A 163 7.212 26.746 27.103 1.00 0.59 N

ATOM 1152 CA TRP A 163 5.851 26.220 26.957 1.00 0.59 C

ATOM 1153 C TRP A 163 5.622 25.076 27.944 1.00 0.59 C

ATOM 1154 O TRP A 163 4.529 24.935 28.471 1.00 0.59 O

ATOM 1155 CB TRP A 163 5.587 25.775 25.522 1.00 0.59 C

ATOM 1156 CG TRP A 163 5.644 26.921 24.528 1.00 0.59 C

ATOM 1157 CD1 TRP A 163 6.661 27.298 23.788 1.00 0.59 C

ATOM 1158 CD2 TRP A 163 4.668 27.796 24.163 1.00 0.59 C

ATOM 1159 CE2 TRP A 163 5.122 28.652 23.262 1.00 0.59 C

ATOM 1160 CE3 TRP A 163 3.438 27.991 24.569 1.00 0.59 C

ATOM 1161 NE1 TRP A 163 6.351 28.333 23.029 1.00 0.59 N

ATOM 1162 CZ2 TRP A 163 4.280 29.620 22.850 1.00 0.59 C

ATOM 1163 CZ3 TRP A 163 2.446 28.846 24.163 1.00 0.59 C

ATOM 1164 CH2 TRP A 163 3.006 29.685 23.262 1.00 0.59 C

ATOM 1165 N GLY A 164 6.698 24.336 28.246 1.00 0.77 N

ATOM 1166 CA GLY A 164 6.661 23.211 29.196 1.00 0.77 C

ATOM 1167 C GLY A 164 6.290 23.743 30.585 1.00 0.77 C

ATOM 1168 O GLY A 164 5.493 23.216 31.345 1.00 0.77 O

ATOM 1169 N ASP A 165 6.870 24.873 30.926 1.00 0.62 N

ATOM 1170 CA ASP A 165 6.588 25.508 32.221 1.00 0.62 C

ATOM 1171 C ASP A 165 5.178 26.080 32.286 1.00 0.62 C

ATOM 1172 O ASP A 165 4.498 25.918 33.290 1.00 0.62 O

ATOM 1173 CB ASP A 165 7.606 26.602 32.514 1.00 0.62 C

ATOM 1174 CG ASP A 165 9.009 26.026 32.721 1.00 0.62 C

ATOM 1175 OD1 ASP A 165 9.111 24.811 33.007 1.00 0.62 O

ATOM 1176 OD2 ASP A 165 9.953 26.838 32.651 1.00 0.62 O1-

ATOM 1177 N GLN A 166 4.745 26.701 31.196 1.00 0.60 N

ATOM 1178 CA GLN A 166 3.384 27.257 31.162 1.00 0.60 C

ATOM 1179 C GLN A 166 2.333 26.149 31.272 1.00 0.60 C

ATOM 1180 O GLN A 166 1.359 26.295 32.000 1.00 0.60 O

ATOM 1181 CB GLN A 166 3.169 28.112 29.921 1.00 0.60 C

ATOM 1182 CG GLN A 166 1.753 28.765 29.858 1.00 0.60 C

ATOM 1183 CD GLN A 166 1.435 29.559 28.559 1.00 0.60 C

ATOM 1184 NE2 GLN A 166 0.406 29.151 27.804 1.00 0.60 N

ATOM 1185 OE1 GLN A 166 2.279 30.286 28.052 1.00 0.60 O

ATOM 1186 N ILE A 167 2.609 25.015 30.642 1.00 0.52 N

ATOM 1187 CA ILE A 167 1.699 23.856 30.702 1.00 0.52 C

ATOM 1188 C ILE A 167 1.601 23.295 32.125 1.00 0.52 C

ATOM 1189 O ILE A 167 0.540 22.847 32.544 1.00 0.52 O

ATOM 1190 CB ILE A 167 2.097 22.732 29.735 1.00 0.52 C

ATOM 1191 CG1 ILE A 167 3.421 22.082 30.082 1.00 0.52 C

ATOM 1192 CG2 ILE A 167 2.307 23.283 28.360 1.00 0.52 C

ATOM 1193 CD1 ILE A 167 3.985 20.973 29.194 1.00 0.52 C

ATOM 1194 N ALA A 168 2.742 23.294 32.811 1.00 0.55 N

ATOM 1195 CA ALA A 168 2.844 22.749 34.171 1.00 0.55 C

ATOM 1196 C ALA A 168 2.380 23.744 35.243 1.00 0.55 C

ATOM 1197 O ALA A 168 1.844 23.343 36.272 1.00 0.55 O

ATOM 1198 CB ALA A 168 4.288 22.325 34.443 1.00 0.55 C

ATOM 1199 N TYR A 169 2.560 25.031 34.974 1.00 0.36 N

ATOM 1200 CA TYR A 169 2.347 26.088 35.977 1.00 0.36 C

ATOM 1201 C TYR A 169 1.201 27.040 35.608 1.00 0.36 C

ATOM 1202 O TYR A 169 0.712 27.720 36.535 1.00 0.36 O

ATOM 1203 CB TYR A 169 3.657 26.857 36.186 1.00 0.36 C

ATOM 1204 CG TYR A 169 3.507 27.935 37.261 1.00 0.36 C

ATOM 1205 CD1 TYR A 169 3.586 27.586 38.602 1.00 0.36 C

ATOM 1206 CD2 TYR A 169 3.286 29.257 36.893 1.00 0.36 C

ATOM 1207 CE1 TYR A 169 3.459 28.563 39.579 1.00 0.36 C

ATOM 1208 CE2 TYR A 169 3.158 30.235 37.871 1.00 0.36 C

ATOM 1209 CZ TYR A 169 3.252 29.888 39.213 1.00 0.36 C

ATOM 1210 OH TYR A 169 3.181 30.848 40.170 1.00 0.36 O

ATOM 1211 OXT TYR A 169 0.906 27.150 34.402 1.00 0.36 O1-

TER 1212 TYR A 169

ATOM 1213 N ILE B 21 28.205 21.602 14.061 1.00 0.59 N

ATOM 1214 CA ILE B 21 27.046 22.460 13.719 1.00 0.59 C

ATOM 1215 C ILE B 21 25.787 21.644 13.379 1.00 0.59 C

ATOM 1216 O ILE B 21 24.673 21.973 13.773 1.00 0.59 O

ATOM 1217 CB ILE B 21 27.382 23.371 12.525 1.00 0.59 C

ATOM 1218 CG1 ILE B 21 27.716 22.550 11.266 1.00 0.59 C

ATOM 1219 CG2 ILE B 21 28.526 24.325 12.905 1.00 0.59 C

ATOM 1220 CD1 ILE B 21 27.909 23.393 10.007 1.00 0.59 C

ATOM 1221 N SER B 22 26.017 20.520 12.717 1.00 0.72 N

ATOM 1222 CA SER B 22 24.952 19.639 12.256 1.00 0.72 C

ATOM 1223 C SER B 22 24.374 18.831 13.423 1.00 0.72 C

ATOM 1224 O SER B 22 23.173 18.599 13.506 1.00 0.72 O

ATOM 1225 CB SER B 22 25.667 18.767 11.233 1.00 0.72 C

ATOM 1226 OG SER B 22 25.722 19.457 9.982 1.00 0.72 O

ATOM 1227 N GLY B 23 25.267 18.492 14.364 1.00 0.78 N

ATOM 1228 CA GLY B 23 24.893 17.807 15.614 1.00 0.78 C

ATOM 1229 C GLY B 23 23.996 18.726 16.463 1.00 0.78 C

ATOM 1230 O GLY B 23 23.000 18.366 17.079 1.00 0.78 O

ATOM 1231 N LEU B 24 24.368 19.989 16.501 1.00 0.69 N

ATOM 1232 CA LEU B 24 23.593 20.991 17.241 1.00 0.69 C

ATOM 1233 C LEU B 24 22.245 21.256 16.552 1.00 0.69 C

ATOM 1234 O LEU B 24 21.205 21.291 17.208 1.00 0.69 O

ATOM 1235 CB LEU B 24 24.492 22.222 17.368 1.00 0.69 C

ATOM 1236 CG LEU B 24 23.836 23.365 18.125 1.00 0.69 C

ATOM 1237 CD1 LEU B 24 24.851 24.285 18.792 1.00 0.69 C

ATOM 1238 CD2 LEU B 24 23.028 24.197 17.124 1.00 0.69 C

ATOM 1239 N TYR B 25 22.289 21.426 15.234 1.00 0.68 N

ATOM 1240 CA TYR B 25 21.070 21.661 14.448 1.00 0.68 C

ATOM 1241 C TYR B 25 20.162 20.425 14.504 1.00 0.68 C

ATOM 1242 O TYR B 25 18.962 20.461 14.654 1.00 0.68 O

ATOM 1243 CB TYR B 25 21.428 21.963 13.000 1.00 0.68 C

ATOM 1244 CG TYR B 25 20.192 22.319 12.194 1.00 0.68 C

ATOM 1245 CD1 TYR B 25 19.681 23.611 12.310 1.00 0.68 C

ATOM 1246 CD2 TYR B 25 19.702 21.344 11.328 1.00 0.68 C

ATOM 1247 CE1 TYR B 25 18.642 23.950 11.530 1.00 0.68 C

ATOM 1248 CE2 TYR B 25 18.701 21.663 10.488 1.00 0.68 C

ATOM 1249 CZ TYR B 25 18.325 22.918 10.718 1.00 0.68 C

ATOM 1250 OH TYR B 25 17.228 23.011 10.357 1.00 0.68 O

ATOM 1251 N VAL B 26 20.710 19.239 14.516 1.00 0.73 N

ATOM 1252 CA VAL B 26 19.868 18.032 14.628 1.00 0.73 C

ATOM 1253 C VAL B 26 19.121 18.008 15.972 1.00 0.73 C

ATOM 1254 O VAL B 26 17.956 17.625 16.033 1.00 0.73 O

ATOM 1255 CB VAL B 26 20.702 16.767 14.410 1.00 0.73 C

ATOM 1256 CG1 VAL B 26 21.750 16.531 15.473 1.00 0.73 C

ATOM 1257 CG2 VAL B 26 19.851 15.508 14.481 1.00 0.73 C

ATOM 1258 N GLU B 27 19.829 18.441 17.013 1.00 0.70 N

ATOM 1259 CA GLU B 27 19.279 18.478 18.365 1.00 0.70 C

ATOM 1260 C GLU B 27 18.113 19.456 18.365 1.00 0.70 C

ATOM 1261 O GLU B 27 17.058 19.071 18.814 1.00 0.70 O

ATOM 1262 CB GLU B 27 20.370 18.869 19.361 1.00 0.70 C

ATOM 1263 CG GLU B 27 19.867 18.942 20.809 1.00 0.70 C

ATOM 1264 CD GLU B 27 19.000 20.178 21.081 1.00 0.70 C

ATOM 1265 OE1 GLU B 27 19.223 21.210 20.410 1.00 0.70 O

ATOM 1266 OE2 GLU B 27 18.185 20.085 22.012 1.00 0.70 O1-

ATOM 1267 N CYS B 28 18.242 20.597 17.711 1.00 0.75 N

ATOM 1268 CA CYS B 28 17.141 21.570 17.632 1.00 0.75 C

ATOM 1269 C CYS B 28 15.961 21.014 16.819 1.00 0.75 C

ATOM 1270 O CYS B 28 14.815 21.058 17.256 1.00 0.75 O

ATOM 1271 CB CYS B 28 17.653 22.899 17.067 1.00 0.75 C

ATOM 1272 SG CYS B 28 18.159 22.815 15.326 1.00 0.75 S

ATOM 1273 N LEU B 29 16.266 20.324 15.728 1.00 0.76 N

ATOM 1274 CA LEU B 29 15.224 19.714 14.884 1.00 0.76 C

ATOM 1275 C LEU B 29 14.476 18.635 15.646 1.00 0.76 C

ATOM 1276 O LEU B 29 13.269 18.536 15.525 1.00 0.76 O

ATOM 1277 CB LEU B 29 15.826 19.069 13.642 1.00 0.76 C

ATOM 1278 CG LEU B 29 16.120 20.043 12.517 1.00 0.76 C

ATOM 1279 CD1 LEU B 29 16.909 21.235 12.953 1.00 0.76 C

ATOM 1280 CD2 LEU B 29 17.042 19.315 11.558 1.00 0.76 C

ATOM 1281 N ARG B 30 15.187 17.902 16.488 1.00 0.70 N

ATOM 1282 CA ARG B 30 14.589 16.839 17.308 1.00 0.70 C

ATOM 1283 C ARG B 30 13.596 17.402 18.331 1.00 0.70 C

ATOM 1284 O ARG B 30 12.629 16.750 18.715 1.00 0.70 O

ATOM 1285 CB ARG B 30 15.693 16.060 18.026 1.00 0.70 C

ATOM 1286 CG ARG B 30 15.141 14.862 18.803 1.00 0.70 C

ATOM 1287 CD ARG B 30 14.502 13.847 17.854 1.00 0.70 C

ATOM 1288 NE ARG B 30 13.958 12.731 18.633 1.00 0.70 N

ATOM 1289 CZ ARG B 30 12.789 12.715 19.243 1.00 0.70 C

ATOM 1290 NH1 ARG B 30 11.986 13.783 19.215 1.00 0.70 N1+

ATOM 1291 NH2 ARG B 30 12.431 11.581 19.831 1.00 0.70 N

ATOM 1292 N LEU B 31 13.861 18.615 18.787 1.00 0.76 N

ATOM 1293 CA LEU B 31 12.974 19.263 19.763 1.00 0.76 C

ATOM 1294 C LEU B 31 11.629 19.696 19.195 1.00 0.76 C

ATOM 1295 O LEU B 31 10.722 20.024 19.960 1.00 0.76 O

ATOM 1296 CB LEU B 31 13.628 20.492 20.343 1.00 0.76 C

ATOM 1297 CG LEU B 31 14.595 20.255 21.485 1.00 0.76 C

ATOM 1298 CD1 LEU B 31 15.675 19.273 21.118 1.00 0.76 C

ATOM 1299 CD2 LEU B 31 15.208 21.526 22.074 1.00 0.76 C

ATOM 1300 N VAL B 32 11.494 19.651 17.873 1.00 0.79 N

ATOM 1301 CA VAL B 32 10.245 20.072 17.215 1.00 0.79 C

ATOM 1302 C VAL B 32 9.027 19.303 17.759 1.00 0.79 C

ATOM 1303 O VAL B 32 7.974 19.888 18.002 1.00 0.79 O

ATOM 1304 CB VAL B 32 10.346 19.921 15.687 1.00 0.79 C

ATOM 1305 CG1 VAL B 32 10.495 18.472 15.226 1.00 0.79 C

ATOM 1306 CG2 VAL B 32 9.108 20.501 15.001 1.00 0.79 C

ATOM 1307 N GLU B 33 9.202 18.000 17.959 1.00 0.73 N

ATOM 1308 CA GLU B 33 8.099 17.130 18.396 1.00 0.73 C

ATOM 1309 C GLU B 33 7.612 17.525 19.789 1.00 0.73 C

ATOM 1310 O GLU B 33 6.443 17.876 19.948 1.00 0.73 O

ATOM 1311 CB GLU B 33 8.463 15.645 18.380 1.00 0.73 C

ATOM 1312 CG GLU B 33 8.677 15.080 16.970 1.00 0.73 C

ATOM 1313 CD GLU B 33 10.030 15.441 16.344 1.00 0.73 C

ATOM 1314 OE1 GLU B 33 10.854 16.064 17.043 1.00 0.73 O

ATOM 1315 OE2 GLU B 33 10.197 15.137 15.147 1.00 0.73 O1-

ATOM 1316 N ARG B 34 8.518 17.505 20.767 1.00 0.70 N

ATOM 1317 CA ARG B 34 8.161 17.923 22.137 1.00 0.70 C

ATOM 1318 C ARG B 34 7.703 19.388 22.207 1.00 0.70 C

ATOM 1319 O ARG B 34 6.779 19.716 22.948 1.00 0.70 O

ATOM 1320 CB ARG B 34 9.306 17.671 23.121 1.00 0.70 C

ATOM 1321 CG ARG B 34 10.570 18.479 22.816 1.00 0.70 C

ATOM 1322 CD ARG B 34 11.610 18.203 23.891 1.00 0.70 C

ATOM 1323 NE ARG B 34 12.843 18.963 23.639 1.00 0.70 N

ATOM 1324 CZ ARG B 34 13.926 18.910 24.425 1.00 0.70 C

ATOM 1325 NH1 ARG B 34 13.961 18.159 25.508 1.00 0.70 N1+

ATOM 1326 NH2 ARG B 34 15.011 19.609 24.155 1.00 0.70 N

ATOM 1327 N LEU B 35 8.294 20.239 21.364 1.00 0.77 N

ATOM 1328 CA LEU B 35 7.883 21.649 21.245 1.00 0.77 C

ATOM 1329 C LEU B 35 6.408 21.798 20.926 1.00 0.77 C

ATOM 1330 O LEU B 35 5.701 22.570 21.569 1.00 0.77 O

ATOM 1331 CB LEU B 35 8.604 22.371 20.098 1.00 0.77 C

ATOM 1332 CG LEU B 35 8.297 23.880 20.058 1.00 0.77 C

ATOM 1333 CD1 LEU B 35 8.350 24.623 21.405 1.00 0.77 C

ATOM 1334 CD2 LEU B 35 9.232 24.542 19.051 1.00 0.77 C

ATOM 1335 N HIS B 36 5.992 21.040 19.923 1.00 0.78 N

ATOM 1336 CA HIS B 36 4.611 21.097 19.442 1.00 0.78 C

ATOM 1337 C HIS B 36 3.640 20.621 20.530 1.00 0.78 C

ATOM 1338 O HIS B 36 2.609 21.246 20.778 1.00 0.78 O

ATOM 1339 CB HIS B 36 4.488 20.240 18.180 1.00 0.78 C

ATOM 1340 CG HIS B 36 3.086 20.284 17.561 1.00 0.78 C

ATOM 1341 CD2 HIS B 36 2.131 21.190 17.780 1.00 0.78 C

ATOM 1342 ND1 HIS B 36 2.598 19.388 16.712 1.00 0.78 N

ATOM 1343 CE1 HIS B 36 1.351 19.739 16.416 1.00 0.78 C

ATOM 1344 NE2 HIS B 36 1.056 20.849 17.085 1.00 0.78 N

ATOM 1345 N ARG B 37 4.041 19.550 21.208 1.00 0.69 N

ATOM 1346 CA ARG B 37 3.279 18.994 22.336 1.00 0.69 C

ATOM 1347 C ARG B 37 3.132 20.032 23.458 1.00 0.69 C

ATOM 1348 O ARG B 37 2.042 20.250 23.978 1.00 0.69 O

ATOM 1349 CB ARG B 37 4.033 17.777 22.867 1.00 0.69 C

ATOM 1350 CG ARG B 37 3.245 17.061 23.968 1.00 0.69 C

ATOM 1351 CD ARG B 37 4.125 16.067 24.719 1.00 0.69 C

ATOM 1352 NE ARG B 37 5.214 16.793 25.396 1.00 0.69 N

ATOM 1353 CZ ARG B 37 6.495 16.422 25.437 1.00 0.69 C

ATOM 1354 NH1 ARG B 37 6.906 15.295 24.860 1.00 0.69 N1+

ATOM 1355 NH2 ARG B 37 7.381 17.192 26.060 1.00 0.69 N

ATOM 1356 N SER B 38 4.229 20.724 23.739 1.00 0.74 N

ATOM 1357 CA SER B 38 4.277 21.725 24.806 1.00 0.74 C

ATOM 1358 C SER B 38 3.478 22.971 24.446 1.00 0.74 C

ATOM 1359 O SER B 38 2.802 23.506 25.312 1.00 0.74 O

ATOM 1360 CB SER B 38 5.726 22.103 25.047 1.00 0.74 C

ATOM 1361 OG SER B 38 5.745 22.993 26.139 1.00 0.74 O

ATOM 1362 N LEU B 39 3.538 23.369 23.182 1.00 0.72 N

ATOM 1363 CA LEU B 39 2.775 24.513 22.684 1.00 0.72 C

ATOM 1364 C LEU B 39 1.267 24.243 22.798 1.00 0.72 C

ATOM 1365 O LEU B 39 0.428 25.054 23.151 1.00 0.72 O

ATOM 1366 CB LEU B 39 3.130 24.730 21.227 1.00 0.72 C

ATOM 1367 CG LEU B 39 2.341 25.890 20.661 1.00 0.72 C

ATOM 1368 CD1 LEU B 39 2.748 27.185 21.336 1.00 0.72 C

ATOM 1369 CD2 LEU B 39 2.523 25.791 19.159 1.00 0.72 C

ATOM 1370 N LEU B 40 0.873 23.038 22.482 1.00 0.69 N

ATOM 1371 CA LEU B 40 -0.545 22.672 22.559 1.00 0.69 C

ATOM 1372 C LEU B 40 -1.023 22.676 24.017 1.00 0.69 C

ATOM 1373 O LEU B 40 -2.087 23.215 24.329 1.00 0.69 O

ATOM 1374 CB LEU B 40 -0.713 21.321 21.871 1.00 0.69 C

ATOM 1375 CG LEU B 40 -2.176 20.874 21.818 1.00 0.69 C

ATOM 1376 CD1 LEU B 40 -2.354 19.872 20.680 1.00 0.69 C

ATOM 1377 CD2 LEU B 40 -2.608 20.199 23.126 1.00 0.69 C

ATOM 1378 N ASP B 41 -0.214 22.078 24.887 1.00 0.66 N

ATOM 1379 CA ASP B 41 -0.552 22.000 26.312 1.00 0.66 C

ATOM 1380 C ASP B 41 -0.597 23.376 26.979 1.00 0.66 C

ATOM 1381 O ASP B 41 -1.376 23.734 27.841 1.00 0.66 O

ATOM 1382 CB ASP B 41 0.419 21.079 27.040 1.00 0.66 C

ATOM 1383 CG ASP B 41 0.359 19.632 26.578 1.00 0.66 C

ATOM 1384 OD1 ASP B 41 -0.724 19.045 26.592 1.00 0.66 O

ATOM 1385 OD2 ASP B 41 1.400 19.131 26.130 1.00 0.66 O1-

ATOM 1386 N VAL B 42 0.290 24.219 26.588 1.00 0.62 N

ATOM 1387 CA VAL B 42 0.334 25.558 27.180 1.00 0.62 C

ATOM 1388 C VAL B 42 -0.821 26.429 26.680 1.00 0.62 C

ATOM 1389 O VAL B 42 -1.433 27.154 27.454 1.00 0.62 O

ATOM 1390 CB VAL B 42 1.694 25.891 26.700 1.00 0.62 C

ATOM 1391 CG1 VAL B 42 1.625 26.060 25.245 1.00 0.62 C

ATOM 1392 CG2 VAL B 42 2.160 27.126 27.219 1.00 0.62 C

ATOM 1393 N THR B 43 -1.124 26.279 25.398 1.00 0.69 N

ATOM 1394 CA THR B 43 -2.180 27.029 24.760 1.00 0.69 C

ATOM 1395 C THR B 43 -3.497 26.630 25.430 1.00 0.69 C

ATOM 1396 O THR B 43 -4.315 27.490 25.750 1.00 0.69 O

ATOM 1397 CB THR B 43 -2.120 26.622 23.301 1.00 0.69 C

ATOM 1398 CG2 THR B 43 -3.190 27.352 22.635 1.00 0.69 C

ATOM 1399 OG1 THR B 43 -1.032 27.202 22.600 1.00 0.69 O

ATOM 1400 N ARG B 44 -3.630 25.332 25.700 1.00 0.58 N

ATOM 1401 CA ARG B 44 -4.853 24.803 26.319 1.00 0.58 C

ATOM 1402 C ARG B 44 -5.077 25.417 27.711 1.00 0.58 C

ATOM 1403 O ARG B 44 -6.189 25.742 28.094 1.00 0.58 O

ATOM 1404 CB ARG B 44 -4.833 23.273 26.383 1.00 0.58 C

ATOM 1405 CG ARG B 44 -3.795 22.751 27.359 1.00 0.58 C

ATOM 1406 CD ARG B 44 -3.656 21.246 27.424 1.00 0.58 C

ATOM 1407 NE ARG B 44 -2.559 20.835 28.327 1.00 0.58 N

ATOM 1408 CZ ARG B 44 -2.312 19.556 28.627 1.00 0.58 C

ATOM 1409 NH1 ARG B 44 -3.062 18.591 28.090 1.00 0.58 N1+

ATOM 1410 NH2 ARG B 44 -1.250 19.223 29.362 1.00 0.58 N

ATOM 1411 N ASP B 45 -4.002 25.713 28.426 1.00 0.61 N

ATOM 1412 CA ASP B 45 -4.119 26.359 29.745 1.00 0.61 C

ATOM 1413 C ASP B 45 -4.813 27.723 29.632 1.00 0.61 C

ATOM 1414 O ASP B 45 -5.717 28.035 30.401 1.00 0.61 O

ATOM 1415 CB ASP B 45 -2.739 26.546 30.373 1.00 0.61 C

ATOM 1416 CG ASP B 45 -2.064 25.213 30.696 1.00 0.61 C

ATOM 1417 OD1 ASP B 45 -2.644 24.463 31.508 1.00 0.61 O

ATOM 1418 OD2 ASP B 45 -1.005 24.973 30.083 1.00 0.61 O1-

ATOM 1419 N GLU B 46 -4.447 28.460 28.587 1.00 0.66 N

ATOM 1420 CA GLU B 46 -5.008 29.799 28.345 1.00 0.66 C

ATOM 1421 C GLU B 46 -6.435 29.748 27.832 1.00 0.66 C

ATOM 1422 O GLU B 46 -7.297 30.537 28.221 1.00 0.66 O

ATOM 1423 CB GLU B 46 -4.162 30.566 27.337 1.00 0.66 C

ATOM 1424 CG GLU B 46 -2.770 30.850 27.903 1.00 0.66 C

ATOM 1425 CD GLU B 46 -1.974 31.741 26.950 1.00 0.66 C

ATOM 1426 OE1 GLU B 46 -2.486 32.800 26.545 1.00 0.66 O

ATOM 1427 OE2 GLU B 46 -0.835 31.352 26.628 1.00 0.66 O1-

ATOM 1428 N PHE B 47 -6.700 28.761 27.012 1.00 0.58 N

ATOM 1429 CA PHE B 47 -8.023 28.822 26.445 1.00 0.58 C

ATOM 1430 C PHE B 47 -9.128 28.191 27.297 1.00 0.58 C

ATOM 1431 O PHE B 47 -10.260 28.661 27.240 1.00 0.58 O

ATOM 1432 CB PHE B 47 -7.878 28.241 25.104 1.00 0.58 C

ATOM 1433 CG PHE B 47 -7.018 28.227 23.926 1.00 0.58 C

ATOM 1434 CD1 PHE B 47 -6.864 29.518 23.703 1.00 0.58 C

ATOM 1435 CD2 PHE B 47 -6.557 27.207 23.108 1.00 0.58 C

ATOM 1436 CE1 PHE B 47 -6.110 29.803 22.616 1.00 0.58 C

ATOM 1437 CE2 PHE B 47 -5.886 27.600 21.971 1.00 0.58 C

ATOM 1438 CZ PHE B 47 -5.609 28.923 21.764 1.00 0.58 C

ATOM 1439 N GLU B 48 -8.734 27.340 28.246 1.00 0.57 N

ATOM 1440 CA GLU B 48 -9.672 26.791 29.246 1.00 0.57 C

ATOM 1441 C GLU B 48 -10.148 27.891 30.185 1.00 0.57 C

ATOM 1442 O GLU B 48 -11.277 27.824 30.665 1.00 0.57 O

ATOM 1443 CB GLU B 48 -9.033 25.642 30.033 1.00 0.57 C

ATOM 1444 CG GLU B 48 -10.000 24.909 30.959 1.00 0.57 C

ATOM 1445 CD GLU B 48 -10.202 25.749 32.224 1.00 0.57 C

ATOM 1446 OE1 GLU B 48 -9.245 26.470 32.595 1.00 0.57 O

ATOM 1447 OE2 GLU B 48 -11.314 25.661 32.775 1.00 0.57 O1-

ATOM 1448 N ARG B 49 -9.274 28.857 30.445 1.00 0.54 N

ATOM 1449 CA ARG B 49 -9.656 30.023 31.257 1.00 0.54 C

ATOM 1450 C ARG B 49 -10.808 30.809 30.617 1.00 0.54 C

ATOM 1451 O ARG B 49 -11.602 31.426 31.319 1.00 0.54 O

ATOM 1452 CB ARG B 49 -8.469 30.949 31.518 1.00 0.54 C

ATOM 1453 CG ARG B 49 -7.536 30.357 32.578 1.00 0.54 C

ATOM 1454 CD ARG B 49 -6.347 31.275 32.885 1.00 0.54 C

ATOM 1455 NE ARG B 49 -6.755 32.521 33.567 1.00 0.54 N

ATOM 1456 CZ ARG B 49 -6.764 32.715 34.891 1.00 0.54 C

ATOM 1457 NH1 ARG B 49 -6.450 31.727 35.723 1.00 0.54 N1+

ATOM 1458 NH2 ARG B 49 -6.981 33.928 35.393 1.00 0.54 N

ATOM 1459 N GLN B 50 -10.914 30.716 29.292 1.00 0.63 N

ATOM 1460 CA GLN B 50 -12.016 31.349 28.552 1.00 0.63 C

ATOM 1461 C GLN B 50 -13.072 30.323 28.087 1.00 0.63 C

ATOM 1462 O GLN B 50 -13.934 30.577 27.241 1.00 0.63 O

ATOM 1463 CB GLN B 50 -11.386 32.067 27.368 1.00 0.63 C

ATOM 1464 CG GLN B 50 -12.441 32.858 26.622 1.00 0.63 C

ATOM 1465 CD GLN B 50 -13.145 33.963 27.381 1.00 0.63 C

ATOM 1466 NE2 GLN B 50 -14.451 33.859 27.570 1.00 0.63 N

ATOM 1467 OE1 GLN B 50 -12.489 34.922 27.746 1.00 0.63 O

ATOM 1468 N GLY B 51 -13.053 29.171 28.746 1.00 0.56 N

ATOM 1469 CA GLY B 51 -13.998 28.093 28.438 1.00 0.56 C

ATOM 1470 C GLY B 51 -14.902 27.840 29.640 1.00 0.56 C

ATOM 1471 O GLY B 51 -14.525 28.080 30.783 1.00 0.56 O

ATOM 1472 N ARG B 52 -16.105 27.363 29.344 1.00 0.44 N

ATOM 1473 CA ARG B 52 -17.100 27.063 30.393 1.00 0.44 C

ATOM 1474 C ARG B 52 -16.785 25.737 31.102 1.00 0.44 C

ATOM 1475 O ARG B 52 -16.861 25.629 32.319 1.00 0.44 O

ATOM 1476 CB ARG B 52 -18.514 27.050 29.802 1.00 0.44 C

ATOM 1477 CG ARG B 52 -18.700 25.983 28.719 1.00 0.44 C

ATOM 1478 CD ARG B 52 -20.124 26.002 28.195 1.00 0.44 C

ATOM 1479 NE ARG B 52 -20.307 24.961 27.171 1.00 0.44 N

ATOM 1480 CZ ARG B 52 -21.446 24.757 26.508 1.00 0.44 C

ATOM 1481 NH1 ARG B 52 -22.512 25.517 26.746 1.00 0.44 N1+

ATOM 1482 NH2 ARG B 52 -21.532 23.790 25.600 1.00 0.44 N

ATOM 1483 N SER B 53 -16.463 24.732 30.294 1.00 0.44 N

ATOM 1484 CA SER B 53 -16.138 23.384 30.784 1.00 0.44 C

ATOM 1485 C SER B 53 -14.782 22.920 30.246 1.00 0.44 C

ATOM 1486 O SER B 53 -14.160 21.999 30.767 1.00 0.44 O

ATOM 1487 CB SER B 53 -17.245 22.407 30.377 1.00 0.44 C

ATOM 1488 OG SER B 53 -16.974 21.120 30.932 1.00 0.44 O

ATOM 1489 N ASP B 54 -14.362 23.544 29.151 1.00 0.48 N

ATOM 1490 CA ASP B 54 -13.105 23.207 28.482 1.00 0.48 C

ATOM 1491 C ASP B 54 -12.614 24.424 27.711 1.00 0.48 C

ATOM 1492 O ASP B 54 -13.349 25.383 27.516 1.00 0.48 O

ATOM 1493 CB ASP B 54 -13.387 22.015 27.571 1.00 0.48 C

ATOM 1494 CG ASP B 54 -12.126 21.547 26.874 1.00 0.48 C

ATOM 1495 OD1 ASP B 54 -11.221 20.953 27.489 1.00 0.48 O

ATOM 1496 OD2 ASP B 54 -12.076 21.853 25.690 1.00 0.48 O1-

ATOM 1497 N VAL B 55 -11.376 24.343 27.275 1.00 0.56 N

ATOM 1498 CA VAL B 55 -10.776 25.412 26.488 1.00 0.56 C

ATOM 1499 C VAL B 55 -11.652 25.661 25.230 1.00 0.56 C

ATOM 1500 O VAL B 55 -12.335 26.682 25.196 1.00 0.56 O

ATOM 1501 CB VAL B 55 -9.430 24.779 26.216 1.00 0.56 C

ATOM 1502 CG1 VAL B 55 -8.627 25.522 25.275 1.00 0.56 C

ATOM 1503 CG2 VAL B 55 -8.418 24.419 27.230 1.00 0.56 C

ATOM 1504 N ASN B 56 -11.577 24.829 24.170 1.00 0.64 N

ATOM 1505 CA ASN B 56 -10.551 23.826 23.793 1.00 0.64 C

ATOM 1506 C ASN B 56 -9.568 24.421 22.775 1.00 0.64 C

ATOM 1507 O ASN B 56 -9.845 25.484 22.231 1.00 0.64 O

ATOM 1508 CB ASN B 56 -11.024 22.485 23.313 1.00 0.64 C

ATOM 1509 CG ASN B 56 -9.863 21.492 23.322 1.00 0.64 C

ATOM 1510 ND2 ASN B 56 -9.659 20.715 24.366 1.00 0.64 N

ATOM 1511 OD1 ASN B 56 -9.160 21.420 22.329 1.00 0.64 O

ATOM 1512 N ALA B 57 -8.339 23.893 22.745 1.00 0.73 N

ATOM 1513 CA ALA B 57 -7.353 24.296 21.729 1.00 0.73 C

ATOM 1514 C ALA B 57 -7.917 24.203 20.306 1.00 0.73 C

ATOM 1515 O ALA B 57 -7.720 25.107 19.498 1.00 0.73 O

ATOM 1516 CB ALA B 57 -6.104 23.424 21.844 1.00 0.73 C

ATOM 1517 N VAL B 58 -8.676 23.140 20.041 1.00 0.77 N

ATOM 1518 CA VAL B 58 -9.311 22.935 18.725 1.00 0.77 C

ATOM 1519 C VAL B 58 -10.329 24.044 18.429 1.00 0.77 C

ATOM 1520 O VAL B 58 -10.345 24.606 17.338 1.00 0.77 O

ATOM 1521 CB VAL B 58 -9.974 21.549 18.654 1.00 0.77 C

ATOM 1522 CG1 VAL B 58 -11.077 21.352 19.703 1.00 0.77 C

ATOM 1523 CG2 VAL B 58 -10.518 21.284 17.245 1.00 0.77 C

ATOM 1524 N GLN B 59 -11.127 24.359 19.442 1.00 0.76 N

ATOM 1525 CA GLN B 59 -12.162 25.393 19.345 1.00 0.76 C

ATOM 1526 C GLN B 59 -11.531 26.760 19.098 1.00 0.76 C

ATOM 1527 O GLN B 59 -11.964 27.500 18.216 1.00 0.76 O

ATOM 1528 CB GLN B 59 -12.948 25.398 20.648 1.00 0.76 C

ATOM 1529 CG GLN B 59 -13.675 24.063 20.837 1.00 0.76 C

ATOM 1530 CD GLN B 59 -14.466 24.025 22.133 1.00 0.76 C

ATOM 1531 NE2 GLN B 59 -15.103 22.907 22.343 1.00 0.76 N

ATOM 1532 OE1 GLN B 59 -14.353 24.863 23.011 1.00 0.76 O

ATOM 1533 N ALA B 60 -10.409 26.987 19.771 1.00 0.80 N

ATOM 1534 CA ALA B 60 -9.636 28.219 19.574 1.00 0.80 C

ATOM 1535 C ALA B 60 -9.104 28.348 18.151 1.00 0.80 C

ATOM 1536 O ALA B 60 -9.158 29.427 17.566 1.00 0.80 O

ATOM 1537 CB ALA B 60 -8.461 28.213 20.517 1.00 0.80 C

ATOM 1538 N LEU B 61 -8.721 27.218 17.572 1.00 0.78 N

ATOM 1539 CA LEU B 61 -8.198 27.228 16.214 1.00 0.78 C

ATOM 1540 C LEU B 61 -9.279 27.683 15.236 1.00 0.78 C

ATOM 1541 O LEU B 61 -9.006 28.547 14.413 1.00 0.78 O

ATOM 1542 CB LEU B 61 -7.727 25.832 15.916 1.00 0.78 C

ATOM 1543 CG LEU B 61 -7.042 25.853 14.596 1.00 0.78 C

ATOM 1544 CD1 LEU B 61 -5.859 24.994 15.003 1.00 0.78 C

ATOM 1545 CD2 LEU B 61 -8.085 25.614 13.484 1.00 0.78 C

ATOM 1546 N LEU B 62 -10.494 27.192 15.432 1.00 0.80 N

ATOM 1547 CA LEU B 62 -11.607 27.584 14.560 1.00 0.80 C

ATOM 1548 C LEU B 62 -12.007 29.040 14.776 1.00 0.80 C

ATOM 1549 O LEU B 62 -12.280 29.741 13.805 1.00 0.80 O

ATOM 1550 CB LEU B 62 -12.783 26.695 14.846 1.00 0.80 C

ATOM 1551 CG LEU B 62 -13.976 26.860 13.936 1.00 0.80 C

ATOM 1552 CD1 LEU B 62 -14.641 25.501 14.009 1.00 0.80 C

ATOM 1553 CD2 LEU B 62 -14.917 27.955 14.448 1.00 0.80 C

ATOM 1554 N LEU B 63 -12.044 29.465 16.037 1.00 0.80 N

ATOM 1555 CA LEU B 63 -12.322 30.873 16.361 1.00 0.80 C

ATOM 1556 C LEU B 63 -11.295 31.816 15.733 1.00 0.80 C

ATOM 1557 O LEU B 63 -11.629 32.868 15.194 1.00 0.80 O

ATOM 1558 CB LEU B 63 -12.318 31.100 17.873 1.00 0.80 C

ATOM 1559 CG LEU B 63 -13.436 30.341 18.579 1.00 0.80 C

ATOM 1560 CD1 LEU B 63 -13.377 30.564 20.086 1.00 0.80 C

ATOM 1561 CD2 LEU B 63 -14.798 30.793 18.046 1.00 0.80 C

ATOM 1562 N PHE B 64 -10.040 31.393 15.754 1.00 0.74 N

ATOM 1563 CA PHE B 64 -8.951 32.192 15.175 1.00 0.74 C

ATOM 1564 C PHE B 64 -9.087 32.256 13.652 1.00 0.74 C

ATOM 1565 O PHE B 64 -8.931 33.313 13.043 1.00 0.74 O

ATOM 1566 CB PHE B 64 -7.597 31.598 15.564 1.00 0.74 C

ATOM 1567 CG PHE B 64 -6.444 32.439 15.007 1.00 0.74 C

ATOM 1568 CD1 PHE B 64 -6.035 33.585 15.679 1.00 0.74 C

ATOM 1569 CD2 PHE B 64 -5.789 32.037 13.850 1.00 0.74 C

ATOM 1570 CE1 PHE B 64 -4.963 34.326 15.195 1.00 0.74 C

ATOM 1571 CE2 PHE B 64 -4.716 32.778 13.368 1.00 0.74 C

ATOM 1572 CZ PHE B 64 -4.301 33.920 14.042 1.00 0.74 C

ATOM 1573 N ASN B 65 -9.468 31.127 13.071 1.00 0.74 N

ATOM 1574 CA ASN B 65 -9.677 31.055 11.623 1.00 0.74 C

ATOM 1575 C ASN B 65 -10.817 31.951 11.139 1.00 0.74 C

ATOM 1576 O ASN B 65 -10.669 32.658 10.145 1.00 0.74 O

ATOM 1577 CB ASN B 65 -10.014 29.633 11.279 1.00 0.74 C

ATOM 1578 CG ASN B 65 -8.874 28.670 11.425 1.00 0.74 C

ATOM 1579 ND2 ASN B 65 -7.796 28.867 10.713 1.00 0.74 N

ATOM 1580 OD1 ASN B 65 -9.052 27.694 12.096 1.00 0.74 O

ATOM 1581 N ILE B 66 -11.895 31.985 11.911 1.00 0.72 N

ATOM 1582 CA ILE B 66 -13.040 32.842 11.567 1.00 0.72 C

ATOM 1583 C ILE B 66 -12.753 34.329 11.819 1.00 0.72 C

ATOM 1584 O ILE B 66 -13.350 35.208 11.203 1.00 0.72 O

ATOM 1585 CB ILE B 66 -14.306 32.373 12.287 1.00 0.72 C

ATOM 1586 CG1 ILE B 66 -15.535 33.211 11.943 1.00 0.72 C

ATOM 1587 CG2 ILE B 66 -14.063 32.356 13.770 1.00 0.72 C

ATOM 1588 CD1 ILE B 66 -15.766 33.078 10.447 1.00 0.72 C

ATOM 1589 N GLY B 67 -11.878 34.565 12.802 1.00 0.78 N

ATOM 1590 CA GLY B 67 -11.431 35.926 13.129 1.00 0.78 C

ATOM 1591 C GLY B 67 -10.667 36.505 11.936 1.00 0.78 C

ATOM 1592 O GLY B 67 -10.832 37.668 11.580 1.00 0.78 O

ATOM 1593 N ASP B 68 -9.890 35.627 11.302 1.00 0.66 N

ATOM 1594 CA ASP B 68 -9.073 35.997 10.142 1.00 0.66 C

ATOM 1595 C ASP B 68 -9.953 36.362 8.939 1.00 0.66 C

ATOM 1596 O ASP B 68 -9.627 37.259 8.165 1.00 0.66 O

ATOM 1597 CB ASP B 68 -8.120 34.848 9.797 1.00 0.66 C

ATOM 1598 CG ASP B 68 -7.109 35.242 8.710 1.00 0.66 C

ATOM 1599 OD1 ASP B 68 -7.434 36.078 7.845 1.00 0.66 O

ATOM 1600 OD2 ASP B 68 -5.989 34.700 8.762 1.00 0.66 O1-

ATOM 1601 N LEU B 69 -11.060 35.649 8.792 1.00 0.68 N

ATOM 1602 CA LEU B 69 -11.968 35.859 7.661 1.00 0.68 C

ATOM 1603 C LEU B 69 -13.268 35.080 7.939 1.00 0.68 C

ATOM 1604 O LEU B 69 -13.286 34.121 8.704 1.00 0.68 O

ATOM 1605 CB LEU B 69 -11.210 35.434 6.378 1.00 0.68 C

ATOM 1606 CG LEU B 69 -12.064 35.491 5.110 1.00 0.68 C

ATOM 1607 CD1 LEU B 69 -12.165 36.950 4.734 1.00 0.68 C

ATOM 1608 CD2 LEU B 69 -11.516 34.769 3.885 1.00 0.68 C

ATOM 1609 N GLU B 70 -14.364 35.493 7.323 1.00 0.71 N

ATOM 1610 CA GLU B 70 -15.625 34.707 7.233 1.00 0.71 C

ATOM 1611 C GLU B 70 -15.286 33.509 6.352 1.00 0.71 C

ATOM 1612 O GLU B 70 -14.618 33.771 5.359 1.00 0.71 O

ATOM 1613 CB GLU B 70 -16.739 35.439 6.456 1.00 0.71 C

ATOM 1614 CG GLU B 70 -16.534 35.683 4.937 1.00 0.71 C

ATOM 1615 CD GLU B 70 -15.381 36.643 4.619 1.00 0.71 C

ATOM 1616 OE1 GLU B 70 -14.851 37.268 5.563 1.00 0.71 O

ATOM 1617 OE2 GLU B 70 -15.183 36.931 3.425 1.00 0.71 O1-

ATOM 1618 N LEU B 71 -16.022 32.416 6.331 1.00 0.79 N

ATOM 1619 CA LEU B 71 -15.540 31.225 5.593 1.00 0.79 C

ATOM 1620 C LEU B 71 -16.530 30.070 5.680 1.00 0.79 C

ATOM 1621 O LEU B 71 -17.362 30.005 6.583 1.00 0.79 O

ATOM 1622 CB LEU B 71 -14.191 30.735 6.153 1.00 0.79 C

ATOM 1623 CG LEU B 71 -12.941 31.457 5.632 1.00 0.79 C

ATOM 1624 CD1 LEU B 71 -12.477 32.553 6.505 1.00 0.79 C

ATOM 1625 CD2 LEU B 71 -11.741 30.831 6.260 1.00 0.79 C

ATOM 1626 N THR B 72 -16.436 29.206 4.683 1.00 0.82 N

ATOM 1627 CA THR B 72 -17.137 27.912 4.672 1.00 0.82 C

ATOM 1628 C THR B 72 -16.272 26.915 5.455 1.00 0.82 C

ATOM 1629 O THR B 72 -15.077 27.143 5.669 1.00 0.82 O

ATOM 1630 CB THR B 72 -17.334 27.422 3.231 1.00 0.82 C

ATOM 1631 CG2 THR B 72 -18.090 28.452 2.383 1.00 0.82 C

ATOM 1632 OG1 THR B 72 -16.060 27.109 2.657 1.00 0.82 O

ATOM 1633 N ALA B 73 -16.877 25.796 5.841 1.00 0.83 N

ATOM 1634 CA ALA B 73 -16.153 24.724 6.554 1.00 0.83 C

ATOM 1635 C ALA B 73 -14.962 24.221 5.730 1.00 0.83 C

ATOM 1636 O ALA B 73 -13.848 24.106 6.233 1.00 0.83 O

ATOM 1637 CB ALA B 73 -17.098 23.556 6.829 1.00 0.83 C

ATOM 1638 N GLY B 74 -15.224 24.018 4.432 1.00 0.85 N

ATOM 1639 CA GLY B 74 -14.184 23.672 3.461 1.00 0.85 C

ATOM 1640 C GLY B 74 -12.988 24.613 3.450 1.00 0.85 C

ATOM 1641 O GLY B 74 -11.833 24.196 3.516 1.00 0.85 O

ATOM 1642 N GLU B 75 -13.329 25.893 3.415 1.00 0.72 N

ATOM 1643 CA GLU B 75 -12.311 26.955 3.416 1.00 0.72 C

ATOM 1644 C GLU B 75 -11.501 26.965 4.716 1.00 0.72 C

ATOM 1645 O GLU B 75 -10.282 27.072 4.705 1.00 0.72 O

ATOM 1646 CB GLU B 75 -12.947 28.327 3.209 1.00 0.72 C

ATOM 1647 CG GLU B 75 -13.553 28.465 1.813 1.00 0.72 C

ATOM 1648 CD GLU B 75 -14.154 29.854 1.620 1.00 0.72 C

ATOM 1649 OE1 GLU B 75 -15.115 30.175 2.355 1.00 0.72 O

ATOM 1650 OE2 GLU B 75 -13.633 30.566 0.742 1.00 0.72 O1-

ATOM 1651 N LEU B 76 -12.186 26.819 5.843 1.00 0.77 N

ATOM 1652 CA LEU B 76 -11.505 26.699 7.143 1.00 0.77 C

ATOM 1653 C LEU B 76 -10.555 25.508 7.189 1.00 0.77 C

ATOM 1654 O LEU B 76 -9.406 25.634 7.605 1.00 0.77 O

ATOM 1655 CB LEU B 76 -12.511 26.536 8.274 1.00 0.77 C

ATOM 1656 CG LEU B 76 -13.165 27.855 8.645 1.00 0.77 C

ATOM 1657 CD1 LEU B 76 -14.138 27.581 9.767 1.00 0.77 C

ATOM 1658 CD2 LEU B 76 -12.136 28.834 9.162 1.00 0.77 C

ATOM 1659 N ARG B 77 -11.036 24.384 6.672 1.00 0.70 N

ATOM 1660 CA ARG B 77 -10.225 23.158 6.604 1.00 0.70 C

ATOM 1661 C ARG B 77 -8.940 23.372 5.816 1.00 0.70 C

ATOM 1662 O ARG B 77 -7.874 22.990 6.283 1.00 0.70 O

ATOM 1663 CB ARG B 77 -10.969 22.050 5.897 1.00 0.70 C

ATOM 1664 CG ARG B 77 -12.121 21.623 6.750 1.00 0.70 C

ATOM 1665 CD ARG B 77 -12.865 20.512 6.056 1.00 0.70 C

ATOM 1666 NE ARG B 77 -13.601 20.893 4.872 1.00 0.70 N

ATOM 1667 CZ ARG B 77 -13.180 20.569 3.657 1.00 0.70 C

ATOM 1668 NH1 ARG B 77 -12.071 19.860 3.473 1.00 0.70 N1+

ATOM 1669 NH2 ARG B 77 -13.866 20.985 2.611 1.00 0.70 N

ATOM 1670 N SER B 78 -9.059 24.039 4.673 1.00 0.76 N

ATOM 1671 CA SER B 78 -7.893 24.334 3.822 1.00 0.76 C

ATOM 1672 C SER B 78 -6.929 25.325 4.488 1.00 0.76 C

ATOM 1673 O SER B 78 -5.720 25.107 4.481 1.00 0.76 O

ATOM 1674 CB SER B 78 -8.331 24.880 2.462 1.00 0.76 C

ATOM 1675 OG SER B 78 -9.040 26.106 2.633 1.00 0.76 O

ATOM 1676 N ARG B 79 -7.490 26.336 5.150 1.00 0.69 N

ATOM 1677 CA ARG B 79 -6.690 27.395 5.786 1.00 0.69 C

ATOM 1678 C ARG B 79 -5.935 26.871 7.015 1.00 0.69 C

ATOM 1679 O ARG B 79 -4.793 27.256 7.261 1.00 0.69 O

ATOM 1680 CB ARG B 79 -7.594 28.565 6.173 1.00 0.69 C

ATOM 1681 CG ARG B 79 -6.771 29.704 6.781 1.00 0.69 C

ATOM 1682 CD ARG B 79 -7.651 30.871 7.192 1.00 0.69 C

ATOM 1683 NE ARG B 79 -8.242 31.484 5.996 1.00 0.69 N

ATOM 1684 CZ ARG B 79 -9.107 32.483 6.030 1.00 0.69 C

ATOM 1685 NH1 ARG B 79 -9.534 32.985 7.186 1.00 0.69 N1+

ATOM 1686 NH2 ARG B 79 -9.607 32.929 4.893 1.00 0.69 N

ATOM 1687 N GLY B 80 -6.615 26.003 7.767 1.00 0.81 N

ATOM 1688 CA GLY B 80 -6.057 25.443 9.003 1.00 0.81 C

ATOM 1689 C GLY B 80 -5.364 24.108 8.760 1.00 0.81 C

ATOM 1690 O GLY B 80 -4.868 23.539 9.725 1.00 0.81 O

ATOM 1691 N TYR B 81 -5.408 23.612 7.518 1.00 0.70 N

ATOM 1692 CA TYR B 81 -4.845 22.303 7.134 1.00 0.70 C

ATOM 1693 C TYR B 81 -5.377 21.193 8.060 1.00 0.70 C

ATOM 1694 O TYR B 81 -4.657 20.401 8.654 1.00 0.70 O

ATOM 1695 CB TYR B 81 -3.305 22.357 7.107 1.00 0.70 C

ATOM 1696 CG TYR B 81 -2.650 22.658 8.465 1.00 0.70 C

ATOM 1697 CD1 TYR B 81 -2.396 21.635 9.371 1.00 0.70 C

ATOM 1698 CD2 TYR B 81 -2.386 23.974 8.833 1.00 0.70 C

ATOM 1699 CE1 TYR B 81 -1.916 21.919 10.632 1.00 0.70 C

ATOM 1700 CE2 TYR B 81 -1.891 24.261 10.097 1.00 0.70 C

ATOM 1701 CZ TYR B 81 -1.650 23.232 10.991 1.00 0.70 C

ATOM 1702 OH TYR B 81 -1.088 23.505 12.194 1.00 0.70 O

ATOM 1703 N TYR B 82 -6.688 21.168 8.229 1.00 0.71 N

ATOM 1704 CA TYR B 82 -7.361 20.166 9.068 1.00 0.71 C

ATOM 1705 C TYR B 82 -8.390 19.404 8.258 1.00 0.71 C

ATOM 1706 O TYR B 82 -8.956 19.929 7.303 1.00 0.71 O

ATOM 1707 CB TYR B 82 -8.034 20.839 10.256 1.00 0.71 C

ATOM 1708 CG TYR B 82 -6.995 21.470 11.160 1.00 0.71 C

ATOM 1709 CD1 TYR B 82 -5.712 20.959 11.346 1.00 0.71 C

ATOM 1710 CD2 TYR B 82 -7.367 22.582 11.839 1.00 0.71 C

ATOM 1711 CE1 TYR B 82 -4.774 21.525 12.188 1.00 0.71 C

ATOM 1712 CE2 TYR B 82 -6.398 23.080 12.637 1.00 0.71 C

ATOM 1713 CZ TYR B 82 -5.113 22.643 12.868 1.00 0.71 C

ATOM 1714 OH TYR B 82 -4.187 23.232 13.673 1.00 0.71 O

ATOM 1715 N LEU B 83 -8.595 18.155 8.654 1.00 0.69 N

ATOM 1716 CA LEU B 83 -9.547 17.275 7.966 1.00 0.69 C

ATOM 1717 C LEU B 83 -10.982 17.770 8.216 1.00 0.69 C

ATOM 1718 O LEU B 83 -11.306 18.277 9.292 1.00 0.69 O

ATOM 1719 CB LEU B 83 -9.299 15.837 8.440 1.00 0.69 C

ATOM 1720 CG LEU B 83 -10.082 14.803 7.626 1.00 0.69 C

ATOM 1721 CD1 LEU B 83 -9.463 13.420 7.737 1.00 0.69 C

ATOM 1722 CD2 LEU B 83 -11.445 14.593 8.219 1.00 0.69 C

ATOM 1723 N GLY B 84 -11.837 17.480 7.227 1.00 0.80 N

ATOM 1724 CA GLY B 84 -13.286 17.757 7.269 1.00 0.80 C

ATOM 1725 C GLY B 84 -14.004 17.369 8.548 1.00 0.80 C

ATOM 1726 O GLY B 84 -14.775 18.077 9.172 1.00 0.80 O

ATOM 1727 N SER B 85 -13.725 16.156 8.935 1.00 0.75 N

ATOM 1728 CA SER B 85 -14.335 15.549 10.124 1.00 0.75 C

ATOM 1729 C SER B 85 -14.027 16.358 11.386 1.00 0.75 C

ATOM 1730 O SER B 85 -14.928 16.627 12.174 1.00 0.75 O

ATOM 1731 CB SER B 85 -13.834 14.122 10.309 1.00 0.75 C

ATOM 1732 OG SER B 85 -14.426 13.528 11.449 1.00 0.75 O

ATOM 1733 N ASN B 86 -12.780 16.819 11.497 1.00 0.75 N

ATOM 1734 CA ASN B 86 -12.348 17.598 12.667 1.00 0.75 C

ATOM 1735 C ASN B 86 -12.982 18.994 12.683 1.00 0.75 C

ATOM 1736 O ASN B 86 -13.502 19.444 13.705 1.00 0.75 O

ATOM 1737 CB ASN B 86 -10.823 17.708 12.684 1.00 0.75 C

ATOM 1738 CG ASN B 86 -10.331 18.413 13.951 1.00 0.75 C

ATOM 1739 ND2 ASN B 86 -9.190 19.057 13.828 1.00 0.75 N

ATOM 1740 OD1 ASN B 86 -10.960 18.391 15.000 1.00 0.75 O

ATOM 1741 N VAL B 87 -12.976 19.641 11.521 1.00 0.81 N

ATOM 1742 CA VAL B 87 -13.589 20.976 11.387 1.00 0.81 C

ATOM 1743 C VAL B 87 -15.097 20.916 11.701 1.00 0.81 C

ATOM 1744 O VAL B 87 -15.615 21.784 12.395 1.00 0.81 O

ATOM 1745 CB VAL B 87 -13.351 21.541 9.984 1.00 0.81 C

ATOM 1746 CG1 VAL B 87 -14.133 20.735 8.972 1.00 0.81 C

ATOM 1747 CG2 VAL B 87 -13.849 22.977 9.837 1.00 0.81 C

ATOM 1748 N SER B 88 -15.752 19.865 11.206 1.00 0.83 N

ATOM 1749 CA SER B 88 -17.204 19.699 11.359 1.00 0.83 C

ATOM 1750 C SER B 88 -17.559 19.536 12.834 1.00 0.83 C

ATOM 1751 O SER B 88 -18.484 20.181 13.325 1.00 0.83 O

ATOM 1752 CB SER B 88 -17.710 18.475 10.598 1.00 0.83 C

ATOM 1753 OG SER B 88 -17.617 18.699 9.191 1.00 0.83 O

ATOM 1754 N TYR B 89 -16.741 18.753 13.538 1.00 0.80 N

ATOM 1755 CA TYR B 89 -16.954 18.501 14.971 1.00 0.80 C

ATOM 1756 C TYR B 89 -16.739 19.782 15.788 1.00 0.80 C

ATOM 1757 O TYR B 89 -17.450 20.124 16.721 1.00 0.80 O

ATOM 1758 CB TYR B 89 -16.012 17.393 15.461 1.00 0.80 C

ATOM 1759 CG TYR B 89 -16.214 16.986 16.932 1.00 0.80 C

ATOM 1760 CD1 TYR B 89 -17.125 15.990 17.266 1.00 0.80 C

ATOM 1761 CD2 TYR B 89 -15.497 17.612 17.941 1.00 0.80 C

ATOM 1762 CE1 TYR B 89 -17.417 15.692 18.600 1.00 0.80 C

ATOM 1763 CE2 TYR B 89 -15.739 17.284 19.270 1.00 0.80 C

ATOM 1764 CZ TYR B 89 -16.718 16.355 19.598 1.00 0.80 C

ATOM 1765 OH TYR B 89 -16.972 16.050 20.894 1.00 0.80 O

ATOM 1766 N ASN B 90 -15.710 20.530 15.450 1.00 0.82 N

ATOM 1767 CA ASN B 90 -15.404 21.758 16.191 1.00 0.82 C

ATOM 1768 C ASN B 90 -16.424 22.871 15.925 1.00 0.82 C

ATOM 1769 O ASN B 90 -16.839 23.576 16.842 1.00 0.82 O

ATOM 1770 CB ASN B 90 -14.000 22.216 15.832 1.00 0.82 C

ATOM 1771 CG ASN B 90 -13.633 23.430 16.668 1.00 0.82 C

ATOM 1772 ND2 ASN B 90 -12.771 24.234 16.130 1.00 0.82 N

ATOM 1773 OD1 ASN B 90 -14.076 23.611 17.781 1.00 0.82 O

ATOM 1774 N LEU B 91 -16.863 22.968 14.673 1.00 0.83 N

ATOM 1775 CA LEU B 91 -17.872 23.967 14.290 1.00 0.83 C

ATOM 1776 C LEU B 91 -19.153 23.764 15.082 1.00 0.83 C

ATOM 1777 O LEU B 91 -19.665 24.713 15.659 1.00 0.83 O

ATOM 1778 CB LEU B 91 -18.203 23.890 12.799 1.00 0.83 C

ATOM 1779 CG LEU B 91 -17.117 24.504 11.919 1.00 0.83 C

ATOM 1780 CD1 LEU B 91 -17.430 24.284 10.448 1.00 0.83 C

ATOM 1781 CD2 LEU B 91 -17.126 26.011 12.096 1.00 0.83 C

ATOM 1782 N LYS B 92 -19.515 22.501 15.274 1.00 0.79 N

ATOM 1783 CA LYS B 92 -20.712 22.155 16.048 1.00 0.79 C

ATOM 1784 C LYS B 92 -20.554 22.514 17.530 1.00 0.79 C

ATOM 1785 O LYS B 92 -21.397 23.162 18.129 1.00 0.79 O

ATOM 1786 CB LYS B 92 -21.114 20.702 15.858 1.00 0.79 C

ATOM 1787 CG LYS B 92 -20.110 19.780 16.492 1.00 0.79 C

ATOM 1788 CD LYS B 92 -20.510 18.362 16.246 1.00 0.79 C

ATOM 1789 CE LYS B 92 -19.476 17.508 16.902 1.00 0.79 C

ATOM 1790 NZ LYS B 92 -19.133 17.668 18.326 1.00 0.79 N1+

ATOM 1791 N LYS B 93 -19.362 22.315 18.054 1.00 0.78 N

ATOM 1792 CA LYS B 93 -19.100 22.610 19.459 1.00 0.78 C

ATOM 1793 C LYS B 93 -19.187 24.117 19.747 1.00 0.78 C

ATOM 1794 O LYS B 93 -19.841 24.538 20.698 1.00 0.78 O

ATOM 1795 CB LYS B 93 -17.727 22.067 19.804 1.00 0.78 C

ATOM 1796 CG LYS B 93 -17.539 22.268 21.293 1.00 0.78 C

ATOM 1797 CD LYS B 93 -18.403 21.430 22.228 1.00 0.78 C

ATOM 1798 CE LYS B 93 -18.042 21.670 23.698 1.00 0.78 C

ATOM 1799 NZ LYS B 93 -18.285 23.057 24.123 1.00 0.78 N1+

ATOM 1800 N LEU B 94 -18.583 24.903 18.866 1.00 0.81 N

ATOM 1801 CA LEU B 94 -18.555 26.368 19.031 1.00 0.81 C

ATOM 1802 C LEU B 94 -19.906 27.001 18.755 1.00 0.81 C

ATOM 1803 O LEU B 94 -20.273 27.987 19.388 1.00 0.81 O

ATOM 1804 CB LEU B 94 -17.506 27.033 18.141 1.00 0.81 C

ATOM 1805 CG LEU B 94 -16.143 26.992 18.810 1.00 0.81 C

ATOM 1806 CD1 LEU B 94 -15.728 25.558 18.900 1.00 0.81 C

ATOM 1807 CD2 LEU B 94 -15.101 27.600 17.912 1.00 0.81 C

ATOM 1808 N ILE B 95 -20.642 26.399 17.827 1.00 0.80 N

ATOM 1809 CA ILE B 95 -21.987 26.889 17.482 1.00 0.80 C

ATOM 1810 C ILE B 95 -22.961 26.633 18.637 1.00 0.80 C

ATOM 1811 O ILE B 95 -23.713 27.516 19.030 1.00 0.80 O

ATOM 1812 CB ILE B 95 -22.571 26.244 16.215 1.00 0.80 C

ATOM 1813 CG1 ILE B 95 -22.742 24.739 16.335 1.00 0.80 C

ATOM 1814 CG2 ILE B 95 -21.763 26.519 14.968 1.00 0.80 C

ATOM 1815 CD1 ILE B 95 -23.356 24.008 15.143 1.00 0.80 C

ATOM 1816 N ASP B 96 -22.858 25.432 19.209 1.00 0.83 N

ATOM 1817 CA ASP B 96 -23.685 25.029 20.356 1.00 0.83 C

ATOM 1818 C ASP B 96 -23.391 25.918 21.562 1.00 0.83 C

ATOM 1819 O ASP B 96 -24.271 26.198 22.368 1.00 0.83 O

ATOM 1820 CB ASP B 96 -23.418 23.567 20.719 1.00 0.83 C

ATOM 1821 CG ASP B 96 -23.896 22.609 19.622 1.00 0.83 C

ATOM 1822 OD1 ASP B 96 -25.105 22.633 19.319 1.00 0.83 O

ATOM 1823 OD2 ASP B 96 -23.076 21.788 19.168 1.00 0.83 O1-

ATOM 1824 N LEU B 97 -22.148 26.377 21.630 1.00 0.80 N

ATOM 1825 CA LEU B 97 -21.744 27.270 22.709 1.00 0.80 C

ATOM 1826 C LEU B 97 -22.137 28.758 22.400 1.00 0.80 C

ATOM 1827 O LEU B 97 -22.143 29.702 23.196 1.00 0.80 O

ATOM 1828 CB LEU B 97 -20.260 26.910 22.949 1.00 0.80 C

ATOM 1829 CG LEU B 97 -19.841 27.405 24.313 1.00 0.80 C

ATOM 1830 CD1 LEU B 97 -18.493 26.949 24.850 1.00 0.80 C

ATOM 1831 CD2 LEU B 97 -19.832 28.839 24.078 1.00 0.80 C

ATOM 1832 N GLY B 98 -22.634 28.986 21.205 1.00 0.89 N

ATOM 1833 CA GLY B 98 -23.040 30.341 20.787 1.00 0.89 C

ATOM 1834 C GLY B 98 -21.832 31.265 20.589 1.00 0.89 C

ATOM 1835 O GLY B 98 -21.919 32.471 20.826 1.00 0.89 O

ATOM 1836 N PHE B 99 -20.764 30.698 20.038 1.00 0.85 N

ATOM 1837 CA PHE B 99 -19.546 31.457 19.709 1.00 0.85 C

ATOM 1838 C PHE B 99 -19.468 31.847 18.238 1.00 0.85 C

ATOM 1839 O PHE B 99 -18.761 32.783 17.870 1.00 0.85 O

ATOM 1840 CB PHE B 99 -18.298 30.684 20.136 1.00 0.85 C

ATOM 1841 CG PHE B 99 -18.202 30.539 21.659 1.00 0.85 C

ATOM 1842 CD1 PHE B 99 -18.738 31.514 22.501 1.00 0.85 C

ATOM 1843 CD2 PHE B 99 -17.402 29.533 22.186 1.00 0.85 C

ATOM 1844 CE1 PHE B 99 -18.508 31.473 23.871 1.00 0.85 C

ATOM 1845 CE2 PHE B 99 -17.103 29.519 23.553 1.00 0.85 C

ATOM 1846 CZ PHE B 99 -17.686 30.471 24.419 1.00 0.85 C

ATOM 1847 N ILE B 100 -20.129 31.051 17.410 1.00 0.81 N

ATOM 1848 CA ILE B 100 -20.243 31.351 15.976 1.00 0.81 C

ATOM 1849 C ILE B 100 -21.709 31.199 15.558 1.00 0.81 C

ATOM 1850 O ILE B 100 -22.493 30.535 16.238 1.00 0.81 O

ATOM 1851 CB ILE B 100 -19.331 30.450 15.124 1.00 0.81 C

ATOM 1852 CG1 ILE B 100 -19.837 29.018 15.114 1.00 0.81 C

ATOM 1853 CG2 ILE B 100 -17.882 30.464 15.631 1.00 0.81 C

ATOM 1854 CD1 ILE B 100 -19.115 28.146 14.084 1.00 0.81 C

ATOM 1855 N LYS B 101 -22.042 31.830 14.447 1.00 0.80 N

ATOM 1856 CA LYS B 101 -23.360 31.664 13.819 1.00 0.80 C

ATOM 1857 C LYS B 101 -23.178 31.366 12.330 1.00 0.80 C

ATOM 1858 O LYS B 101 -22.159 31.720 11.732 1.00 0.80 O

ATOM 1859 CB LYS B 101 -24.212 32.919 14.039 1.00 0.80 C

ATOM 1860 CG LYS B 101 -23.602 34.161 13.387 1.00 0.80 C

ATOM 1861 CD LYS B 101 -24.473 35.385 13.659 1.00 0.80 C

ATOM 1862 CE LYS B 101 -23.897 36.632 12.987 1.00 0.80 C

ATOM 1863 NZ LYS B 101 -23.895 36.502 11.522 1.00 0.80 N1+

ATOM 1864 N HIS B 102 -24.181 30.712 11.766 1.00 0.77 N

ATOM 1865 CA HIS B 102 -24.196 30.423 10.327 1.00 0.77 C

ATOM 1866 C HIS B 102 -25.215 31.352 9.660 1.00 0.77 C

ATOM 1867 O HIS B 102 -26.341 31.511 10.129 1.00 0.77 O

ATOM 1868 CB HIS B 102 -24.583 28.957 10.096 1.00 0.77 C

ATOM 1869 CG HIS B 102 -26.011 28.651 10.565 1.00 0.77 C

ATOM 1870 CD2 HIS B 102 -26.361 28.068 11.705 1.00 0.77 C

ATOM 1871 ND1 HIS B 102 -27.120 28.900 9.872 1.00 0.77 N

ATOM 1872 CE1 HIS B 102 -28.155 28.476 10.587 1.00 0.77 C

ATOM 1873 NE2 HIS B 102 -27.685 27.953 11.714 1.00 0.77 N

ATOM 1874 N GLN B 103 -24.788 31.975 8.580 1.00 0.72 N

ATOM 1875 CA GLN B 103 -25.690 32.802 7.765 1.00 0.72 C

ATOM 1876 C GLN B 103 -25.926 32.121 6.418 1.00 0.72 C

ATOM 1877 O GLN B 103 -25.095 31.343 5.944 1.00 0.72 O

ATOM 1878 CB GLN B 103 -25.098 34.196 7.565 1.00 0.72 C

ATOM 1879 CG GLN B 103 -23.746 34.174 6.840 1.00 0.72 C

ATOM 1880 CD GLN B 103 -23.216 35.586 6.614 1.00 0.72 C

ATOM 1881 NE2 GLN B 103 -22.379 35.724 5.605 1.00 0.72 N

ATOM 1882 OE1 GLN B 103 -23.547 36.531 7.318 1.00 0.72 O

ATOM 1883 N ARG B 104 -27.103 32.382 5.867 1.00 0.59 N

ATOM 1884 CA ARG B 104 -27.484 31.841 4.552 1.00 0.59 C

ATOM 1885 C ARG B 104 -26.523 32.327 3.462 1.00 0.59 C

ATOM 1886 O ARG B 104 -26.012 33.445 3.516 1.00 0.59 O

ATOM 1887 CB ARG B 104 -28.914 32.274 4.216 1.00 0.59 C

ATOM 1888 CG ARG B 104 -29.939 31.698 5.196 1.00 0.59 C

ATOM 1889 CD ARG B 104 -30.006 30.172 5.092 1.00 0.59 C

ATOM 1890 NE ARG B 104 -30.988 29.653 6.057 1.00 0.59 N

ATOM 1891 CZ ARG B 104 -30.757 29.413 7.351 1.00 0.59 C

ATOM 1892 NH1 ARG B 104 -29.557 29.638 7.878 1.00 0.59 N1+

ATOM 1893 NH2 ARG B 104 -31.728 28.938 8.123 1.00 0.59 N

ATOM 1894 N SER B 105 -26.313 31.454 2.489 1.00 0.61 N

ATOM 1895 CA SER B 105 -25.477 31.754 1.314 1.00 0.61 C

ATOM 1896 C SER B 105 -26.373 31.666 0.082 1.00 0.61 C

ATOM 1897 O SER B 105 -26.621 30.584 -0.449 1.00 0.61 O

ATOM 1898 CB SER B 105 -24.333 30.745 1.190 1.00 0.61 C

ATOM 1899 OG SER B 105 -23.489 30.822 2.337 1.00 0.61 O

ATOM 1900 N ARG B 106 -26.900 32.818 -0.322 1.00 0.51 N

ATOM 1901 CA ARG B 106 -27.843 32.881 -1.456 1.00 0.51 C

ATOM 1902 C ARG B 106 -27.238 32.442 -2.800 1.00 0.51 C

ATOM 1903 O ARG B 106 -27.967 32.071 -3.714 1.00 0.51 O

ATOM 1904 CB ARG B 106 -28.485 34.268 -1.576 1.00 0.51 C

ATOM 1905 CG ARG B 106 -27.483 35.388 -1.860 1.00 0.51 C

ATOM 1906 CD ARG B 106 -28.236 36.702 -2.063 1.00 0.51 C

ATOM 1907 NE ARG B 106 -27.287 37.791 -2.356 1.00 0.51 N

ATOM 1908 CZ ARG B 106 -26.569 38.466 -1.454 1.00 0.51 C

ATOM 1909 NH1 ARG B 106 -26.665 38.185 -0.158 1.00 0.51 N1+

ATOM 1910 NH2 ARG B 106 -25.761 39.446 -1.847 1.00 0.51 N

ATOM 1911 N ILE B 107 -25.913 32.508 -2.911 1.00 0.52 N

ATOM 1912 CA ILE B 107 -25.221 32.129 -4.158 1.00 0.52 C

ATOM 1913 C ILE B 107 -25.177 30.601 -4.329 1.00 0.52 C

ATOM 1914 O ILE B 107 -25.407 30.074 -5.411 1.00 0.52 O

ATOM 1915 CB ILE B 107 -23.804 32.726 -4.217 1.00 0.52 C

ATOM 1916 CG1 ILE B 107 -22.905 32.179 -3.096 1.00 0.52 C

ATOM 1917 CG2 ILE B 107 -23.882 34.261 -4.179 1.00 0.52 C

ATOM 1918 CD1 ILE B 107 -21.438 32.584 -3.231 1.00 0.52 C

ATOM 1919 N ASP B 108 -24.824 29.924 -3.239 1.00 0.58 N

ATOM 1920 CA ASP B 108 -24.668 28.466 -3.201 1.00 0.58 C

ATOM 1921 C ASP B 108 -25.110 28.007 -1.814 1.00 0.58 C

ATOM 1922 O ASP B 108 -24.324 28.003 -0.869 1.00 0.58 O

ATOM 1923 CB ASP B 108 -23.203 28.110 -3.485 1.00 0.58 C

ATOM 1924 CG ASP B 108 -23.027 26.596 -3.620 1.00 0.58 C

ATOM 1925 OD1 ASP B 108 -23.578 26.020 -4.571 1.00 0.58 O

ATOM 1926 OD2 ASP B 108 -22.375 26.015 -2.747 1.00 0.58 O1-

ATOM 1927 N LYS B 109 -26.367 27.586 -1.748 1.00 0.58 N

ATOM 1928 CA LYS B 109 -27.038 27.303 -0.464 1.00 0.58 C

ATOM 1929 C LYS B 109 -26.295 26.298 0.430 1.00 0.58 C

ATOM 1930 O LYS B 109 -26.252 26.472 1.644 1.00 0.58 O

ATOM 1931 CB LYS B 109 -28.471 26.820 -0.710 1.00 0.58 C

ATOM 1932 CG LYS B 109 -28.525 25.505 -1.491 1.00 0.58 C

ATOM 1933 CD LYS B 109 -29.967 25.055 -1.705 1.00 0.58 C

ATOM 1934 CE LYS B 109 -30.027 23.720 -2.452 1.00 0.58 C

ATOM 1935 NZ LYS B 109 -29.426 22.632 -1.668 1.00 0.58 N1+

ATOM 1936 N ARG B 110 -25.706 25.273 -0.187 1.00 0.58 N

ATOM 1937 CA ARG B 110 -25.014 24.224 0.582 1.00 0.58 C

ATOM 1938 C ARG B 110 -23.668 24.696 1.157 1.00 0.58 C

ATOM 1939 O ARG B 110 -23.094 24.032 2.018 1.00 0.58 O

ATOM 1940 CB ARG B 110 -24.861 22.944 -0.241 1.00 0.58 C

ATOM 1941 CG ARG B 110 -23.755 23.033 -1.296 1.00 0.58 C

ATOM 1942 CD ARG B 110 -23.707 21.750 -2.114 1.00 0.58 C

ATOM 1943 NE ARG B 110 -24.876 21.715 -3.010 1.00 0.58 N

ATOM 1944 CZ ARG B 110 -24.809 21.677 -4.341 1.00 0.58 C

ATOM 1945 NH1 ARG B 110 -23.630 21.609 -4.954 1.00 0.58 N1+

ATOM 1946 NH2 ARG B 110 -25.914 21.779 -5.070 1.00 0.58 N

ATOM 1947 N SER B 111 -23.141 25.792 0.613 1.00 0.68 N

ATOM 1948 CA SER B 111 -21.882 26.376 1.105 1.00 0.68 C

ATOM 1949 C SER B 111 -22.165 27.277 2.304 1.00 0.68 C

ATOM 1950 O SER B 111 -22.165 28.505 2.208 1.00 0.68 O

ATOM 1951 CB SER B 111 -21.181 27.201 0.024 1.00 0.68 C

ATOM 1952 OG SER B 111 -20.622 26.327 -0.946 1.00 0.68 O

ATOM 1953 N ILE B 112 -22.346 26.639 3.453 1.00 0.69 N

ATOM 1954 CA ILE B 112 -22.646 27.358 4.706 1.00 0.69 C

ATOM 1955 C ILE B 112 -21.452 28.258 5.062 1.00 0.69 C

ATOM 1956 O ILE B 112 -20.297 27.827 4.999 1.00 0.69 O

ATOM 1957 CB ILE B 112 -22.930 26.360 5.841 1.00 0.69 C

ATOM 1958 CG1 ILE B 112 -21.691 25.507 6.165 1.00 0.69 C

ATOM 1959 CG2 ILE B 112 -24.142 25.484 5.475 1.00 0.69 C

ATOM 1960 CD1 ILE B 112 -21.866 24.593 7.377 1.00 0.69 C

ATOM 1961 N ARG B 113 -21.757 29.494 5.422 1.00 0.73 N

ATOM 1962 CA ARG B 113 -20.714 30.443 5.842 1.00 0.73 C

ATOM 1963 C ARG B 113 -20.938 30.836 7.301 1.00 0.73 C

ATOM 1964 O ARG B 113 -22.073 31.024 7.745 1.00 0.73 O

ATOM 1965 CB ARG B 113 -20.704 31.682 4.943 1.00 0.73 C

ATOM 1966 CG ARG B 113 -20.276 31.324 3.519 1.00 0.73 C

ATOM 1967 CD ARG B 113 -20.078 32.585 2.678 1.00 0.73 C

ATOM 1968 NE ARG B 113 -19.611 32.219 1.328 1.00 0.73 N

ATOM 1969 CZ ARG B 113 -18.340 32.010 0.966 1.00 0.73 C

ATOM 1970 NH1 ARG B 113 -17.352 32.109 1.849 1.00 0.73 N1+

ATOM 1971 NH2 ARG B 113 -18.048 31.721 -0.298 1.00 0.73 N

ATOM 1972 N ILE B 114 -19.828 30.982 8.006 1.00 0.78 N

ATOM 1973 CA ILE B 114 -19.851 31.176 9.468 1.00 0.78 C

ATOM 1974 C ILE B 114 -19.175 32.492 9.852 1.00 0.78 C

ATOM 1975 O ILE B 114 -18.263 32.935 9.158 1.00 0.78 O

ATOM 1976 CB ILE B 114 -19.142 30.004 10.158 1.00 0.78 C

ATOM 1977 CG1 ILE B 114 -17.669 29.969 9.741 1.00 0.78 C

ATOM 1978 CG2 ILE B 114 -19.865 28.681 9.844 1.00 0.78 C

ATOM 1979 CD1 ILE B 114 -16.908 28.954 10.557 1.00 0.78 C

ATOM 1980 N SER B 115 -19.673 33.082 10.925 1.00 0.82 N

ATOM 1981 CA SER B 115 -19.098 34.302 11.514 1.00 0.82 C

ATOM 1982 C SER B 115 -19.069 34.165 13.036 1.00 0.82 C

ATOM 1983 O SER B 115 -19.944 33.523 13.620 1.00 0.82 O

ATOM 1984 CB SER B 115 -19.959 35.504 11.122 1.00 0.82 C

ATOM 1985 OG SER B 115 -19.409 36.695 11.686 1.00 0.82 O

ATOM 1986 N LEU B 116 -18.026 34.722 13.643 1.00 0.83 N

ATOM 1987 CA LEU B 116 -17.963 34.858 15.108 1.00 0.83 C

ATOM 1988 C LEU B 116 -19.165 35.655 15.609 1.00 0.83 C

ATOM 1989 O LEU B 116 -19.654 36.560 14.935 1.00 0.83 O

ATOM 1990 CB LEU B 116 -16.712 35.642 15.513 1.00 0.83 C

ATOM 1991 CG LEU B 116 -15.427 34.860 15.296 1.00 0.83 C

ATOM 1992 CD1 LEU B 116 -14.205 35.756 15.491 1.00 0.83 C

ATOM 1993 CD2 LEU B 116 -15.397 33.719 16.312 1.00 0.83 C

ATOM 1994 N THR B 117 -19.649 35.266 16.775 1.00 0.90 N

ATOM 1995 CA THR B 117 -20.644 36.075 17.493 1.00 0.90 C

ATOM 1996 C THR B 117 -19.845 37.045 18.364 1.00 0.90 C

ATOM 1997 O THR B 117 -18.625 36.934 18.509 1.00 0.90 O

ATOM 1998 CB THR B 117 -21.548 35.198 18.366 1.00 0.90 C

ATOM 1999 CG2 THR B 117 -22.361 34.219 17.515 1.00 0.90 C

ATOM 2000 OG1 THR B 117 -20.745 34.520 19.334 1.00 0.90 O

ATOM 2001 N GLN B 118 -20.547 37.973 18.986 1.00 0.78 N

ATOM 2002 CA GLN B 118 -19.923 38.912 19.913 1.00 0.78 C

ATOM 2003 C GLN B 118 -19.133 38.229 21.036 1.00 0.78 C

ATOM 2004 O GLN B 118 -18.067 38.613 21.501 1.00 0.78 O

ATOM 2005 CB GLN B 118 -20.906 39.948 20.481 1.00 0.78 C

ATOM 2006 CG GLN B 118 -21.908 39.382 21.464 1.00 0.78 C

ATOM 2007 CD GLN B 118 -22.953 38.573 20.708 1.00 0.78 C

ATOM 2008 NE2 GLN B 118 -23.837 38.024 21.487 1.00 0.78 N

ATOM 2009 OE1 GLN B 118 -23.033 38.463 19.491 1.00 0.78 O

ATOM 2010 N SER B 119 -19.720 37.175 21.543 1.00 0.86 N

ATOM 2011 CA SER B 119 -19.062 36.380 22.588 1.00 0.86 C

ATOM 2012 C SER B 119 -17.832 35.645 22.042 1.00 0.86 C

ATOM 2013 O SER B 119 -16.765 35.691 22.652 1.00 0.86 O

ATOM 2014 CB SER B 119 -20.047 35.379 23.178 1.00 0.86 C

ATOM 2015 OG SER B 119 -20.495 34.493 22.153 1.00 0.86 O

ATOM 2016 N GLY B 120 -17.977 35.099 20.826 1.00 0.93 N

ATOM 2017 CA GLY B 120 -16.891 34.346 20.172 1.00 0.93 C

ATOM 2018 C GLY B 120 -15.692 35.262 19.912 1.00 0.93 C

ATOM 2019 O GLY B 120 -14.543 34.909 20.179 1.00 0.93 O

ATOM 2020 N LYS B 121 -16.011 36.486 19.494 1.00 0.79 N

ATOM 2021 CA LYS B 121 -14.996 37.522 19.243 1.00 0.79 C

ATOM 2022 C LYS B 121 -14.282 37.936 20.536 1.00 0.79 C

ATOM 2023 O LYS B 121 -13.056 38.014 20.562 1.00 0.79 O

ATOM 2024 CB LYS B 121 -15.614 38.737 18.537 1.00 0.79 C

ATOM 2025 CG LYS B 121 -16.603 39.482 19.419 1.00 0.79 C

ATOM 2026 CD LYS B 121 -17.309 40.665 18.779 1.00 0.79 C

ATOM 2027 CE LYS B 121 -18.268 41.439 19.692 1.00 0.79 C

ATOM 2028 NZ LYS B 121 -17.813 41.776 21.038 1.00 0.79 N1+

ATOM 2029 N GLU B 122 -15.057 38.055 21.616 1.00 0.80 N

ATOM 2030 CA GLU B 122 -14.531 38.445 22.929 1.00 0.80 C

ATOM 2031 C GLU B 122 -13.570 37.368 23.453 1.00 0.80 C

ATOM 2032 O GLU B 122 -12.548 37.640 24.069 1.00 0.80 O

ATOM 2033 CB GLU B 122 -15.691 38.723 23.890 1.00 0.80 C

ATOM 2034 CG GLU B 122 -15.220 39.176 25.282 1.00 0.80 C

ATOM 2035 CD GLU B 122 -14.589 38.044 26.109 1.00 0.80 C

ATOM 2036 OE1 GLU B 122 -15.018 36.883 25.919 1.00 0.80 O

ATOM 2037 OE2 GLU B 122 -13.705 38.363 26.933 1.00 0.80 O1-

ATOM 2038 N ILE B 123 -13.916 36.120 23.221 1.00 0.81 N

ATOM 2039 CA ILE B 123 -13.094 35.001 23.704 1.00 0.81 C

ATOM 2040 C ILE B 123 -11.755 34.935 22.968 1.00 0.81 C

ATOM 2041 O ILE B 123 -10.708 34.813 23.598 1.00 0.81 O

ATOM 2042 CB ILE B 123 -13.885 33.701 23.563 1.00 0.81 C

ATOM 2043 CG1 ILE B 123 -15.007 33.757 24.597 1.00 0.81 C

ATOM 2044 CG2 ILE B 123 -13.000 32.432 23.551 1.00 0.81 C

ATOM 2045 CD1 ILE B 123 -15.749 32.466 24.653 1.00 0.81 C

ATOM 2046 N ALA B 124 -11.824 34.978 21.641 1.00 0.88 N

ATOM 2047 CA ALA B 124 -10.613 34.844 20.813 1.00 0.88 C

ATOM 2048 C ALA B 124 -9.682 36.041 21.029 1.00 0.88 C

ATOM 2049 O ALA B 124 -8.462 35.888 21.069 1.00 0.88 O

ATOM 2050 CB ALA B 124 -10.991 34.735 19.337 1.00 0.88 C

ATOM 2051 N GLU B 125 -10.292 37.209 21.236 1.00 0.83 N

ATOM 2052 CA GLU B 125 -9.532 38.440 21.514 1.00 0.83 C

ATOM 2053 C GLU B 125 -8.779 38.336 22.849 1.00 0.83 C

ATOM 2054 O GLU B 125 -7.593 38.644 22.925 1.00 0.83 O

ATOM 2055 CB GLU B 125 -10.440 39.674 21.484 1.00 0.83 C

ATOM 2056 CG GLU B 125 -11.444 39.668 22.623 1.00 0.83 C

ATOM 2057 CD GLU B 125 -12.426 40.814 22.669 1.00 0.83 C

ATOM 2058 OE1 GLU B 125 -13.092 41.031 21.635 1.00 0.83 O

ATOM 2059 OE2 GLU B 125 -12.502 41.416 23.758 1.00 0.83 O1-

ATOM 2060 N THR B 126 -9.459 37.800 23.862 1.00 0.92 N

ATOM 2061 CA THR B 126 -8.860 37.611 25.195 1.00 0.92 C

ATOM 2062 C THR B 126 -7.725 36.594 25.118 1.00 0.92 C

ATOM 2063 O THR B 126 -6.661 36.734 25.715 1.00 0.92 O

ATOM 2064 CB THR B 126 -9.903 37.142 26.215 1.00 0.92 C

ATOM 2065 CG2 THR B 126 -9.277 36.781 27.569 1.00 0.92 C

ATOM 2066 OG1 THR B 126 -10.866 38.178 26.392 1.00 0.92 O

ATOM 2067 N ILE B 127 -7.983 35.533 24.392 1.00 0.82 N

ATOM 2068 CA ILE B 127 -6.989 34.494 24.283 1.00 0.82 C

ATOM 2069 C ILE B 127 -5.689 35.055 23.641 1.00 0.82 C

ATOM 2070 O ILE B 127 -4.579 34.751 24.088 1.00 0.82 O

ATOM 2071 CB ILE B 127 -7.690 33.395 23.515 1.00 0.82 C

ATOM 2072 CG1 ILE B 127 -8.750 32.602 24.298 1.00 0.82 C

ATOM 2073 CG2 ILE B 127 -6.507 32.537 23.389 1.00 0.82 C

ATOM 2074 CD1 ILE B 127 -9.438 31.493 23.481 1.00 0.82 C

ATOM 2075 N SER B 128 -5.887 35.658 22.478 1.00 0.91 N

ATOM 2076 CA SER B 128 -4.764 36.186 21.690 1.00 0.91 C

ATOM 2077 C SER B 128 -3.956 37.217 22.482 1.00 0.91 C

ATOM 2078 O SER B 128 -2.728 37.226 22.409 1.00 0.91 O

ATOM 2079 CB SER B 128 -5.275 36.819 20.397 1.00 0.91 C

ATOM 2080 OG SER B 128 -6.150 37.904 20.706 1.00 0.91 O

ATOM 2081 N GLN B 129 -4.640 38.009 23.310 1.00 0.86 N

ATOM 2082 CA GLN B 129 -3.962 39.039 24.114 1.00 0.86 C

ATOM 2083 C GLN B 129 -3.106 38.395 25.212 1.00 0.86 C

ATOM 2084 O GLN B 129 -1.997 38.848 25.488 1.00 0.86 O

ATOM 2085 CB GLN B 129 -4.959 40.040 24.706 1.00 0.86 C

ATOM 2086 CG GLN B 129 -5.906 39.408 25.719 1.00 0.86 C

ATOM 2087 CD GLN B 129 -6.909 40.380 26.300 1.00 0.86 C

ATOM 2088 NE2 GLN B 129 -6.878 40.482 27.611 1.00 0.86 N

ATOM 2089 OE1 GLN B 129 -7.647 41.046 25.588 1.00 0.86 O

ATOM 2090 N LEU B 130 -3.618 37.287 25.747 1.00 0.86 N

ATOM 2091 CA LEU B 130 -2.916 36.513 26.775 1.00 0.86 C

ATOM 2092 C LEU B 130 -1.693 35.802 26.177 1.00 0.86 C

ATOM 2093 O LEU B 130 -0.595 35.745 26.718 1.00 0.86 O

ATOM 2094 CB LEU B 130 -3.873 35.497 27.400 1.00 0.86 C

ATOM 2095 CG LEU B 130 -3.223 34.740 28.564 1.00 0.86 C

ATOM 2096 CD1 LEU B 130 -2.887 35.651 29.735 1.00 0.86 C

ATOM 2097 CD2 LEU B 130 -4.179 33.682 29.094 1.00 0.86 C

ATOM 2098 N TYR B 131 -1.872 35.264 24.989 1.00 0.84 N

ATOM 2099 CA TYR B 131 -0.791 34.541 24.311 1.00 0.84 C

ATOM 2100 C TYR B 131 0.352 35.476 23.903 1.00 0.84 C

ATOM 2101 O TYR B 131 1.521 35.149 24.087 1.00 0.84 O

ATOM 2102 CB TYR B 131 -1.362 33.841 23.087 1.00 0.84 C

ATOM 2103 CG TYR B 131 -0.258 33.097 22.356 1.00 0.84 C

ATOM 2104 CD1 TYR B 131 0.042 31.824 22.771 1.00 0.84 C

ATOM 2105 CD2 TYR B 131 0.358 33.640 21.234 1.00 0.84 C

ATOM 2106 CE1 TYR B 131 0.899 31.105 21.993 1.00 0.84 C

ATOM 2107 CE2 TYR B 131 1.328 32.942 20.539 1.00 0.84 C

ATOM 2108 CZ TYR B 131 1.603 31.660 20.937 1.00 0.84 C

ATOM 2109 OH TYR B 131 2.573 30.980 20.307 1.00 0.84 O

ATOM 2110 N GLN B 132 -0.008 36.656 23.408 1.00 0.80 N

ATOM 2111 CA GLN B 132 0.993 37.638 22.958 1.00 0.80 C

ATOM 2112 C GLN B 132 1.757 38.225 24.141 1.00 0.80 C

ATOM 2113 O GLN B 132 2.958 38.476 24.039 1.00 0.80 O

ATOM 2114 CB GLN B 132 0.339 38.764 22.158 1.00 0.80 C

ATOM 2115 CG GLN B 132 -0.212 38.278 20.813 1.00 0.80 C

ATOM 2116 CD GLN B 132 0.885 37.713 19.910 1.00 0.80 C

ATOM 2117 NE2 GLN B 132 1.557 38.607 19.210 1.00 0.80 N

ATOM 2118 OE1 GLN B 132 1.110 36.514 19.831 1.00 0.80 O

ATOM 2119 N ARG B 133 1.051 38.385 25.257 1.00 0.78 N

ATOM 2120 CA ARG B 133 1.678 38.898 26.487 1.00 0.78 C

ATOM 2121 C ARG B 133 2.712 37.902 27.032 1.00 0.78 C

ATOM 2122 O ARG B 133 3.806 38.306 27.422 1.00 0.78 O

ATOM 2123 CB ARG B 133 0.638 39.254 27.549 1.00 0.78 C

ATOM 2124 CG ARG B 133 -0.091 38.029 28.068 1.00 0.78 C

ATOM 2125 CD ARG B 133 -1.117 38.323 29.129 1.00 0.78 C

ATOM 2126 NE ARG B 133 -2.185 39.179 28.598 1.00 0.78 N

ATOM 2127 CZ ARG B 133 -3.149 39.693 29.355 1.00 0.78 C

ATOM 2128 NH1 ARG B 133 -3.212 39.410 30.655 1.00 0.78 N1+

ATOM 2129 NH2 ARG B 133 -4.048 40.507 28.821 1.00 0.78 N

ATOM 2130 N HIS B 134 2.406 36.613 26.881 1.00 0.80 N

ATOM 2131 CA HIS B 134 3.328 35.542 27.289 1.00 0.80 C

ATOM 2132 C HIS B 134 4.533 35.462 26.361 1.00 0.80 C

ATOM 2133 O HIS B 134 5.664 35.286 26.809 1.00 0.80 O

ATOM 2134 CB HIS B 134 2.632 34.180 27.319 1.00 0.80 C

ATOM 2135 CG HIS B 134 1.579 34.069 28.425 1.00 0.80 C

ATOM 2136 CD2 HIS B 134 1.426 34.869 29.479 1.00 0.80 C

ATOM 2137 ND1 HIS B 134 0.656 33.118 28.501 1.00 0.80 N

ATOM 2138 CE1 HIS B 134 -0.059 33.332 29.600 1.00 0.80 C

ATOM 2139 NE2 HIS B 134 0.410 34.420 30.202 1.00 0.80 N

ATOM 2140 N ILE B 135 4.273 35.677 25.074 1.00 0.76 N

ATOM 2141 CA ILE B 135 5.341 35.682 24.072 1.00 0.76 C

ATOM 2142 C ILE B 135 6.267 36.863 24.322 1.00 0.76 C

ATOM 2143 O ILE B 135 7.457 36.760 24.117 1.00 0.76 O

ATOM 2144 CB ILE B 135 4.786 35.719 22.648 1.00 0.76 C

ATOM 2145 CG1 ILE B 135 3.891 34.507 22.362 1.00 0.76 C

ATOM 2146 CG2 ILE B 135 5.922 35.835 21.630 1.00 0.76 C

ATOM 2147 CD1 ILE B 135 4.581 33.151 22.545 1.00 0.76 C

ATOM 2148 N GLU B 136 5.705 37.978 24.737 1.00 0.72 N

ATOM 2149 CA GLU B 136 6.483 39.175 25.067 1.00 0.72 C

ATOM 2150 C GLU B 136 7.393 38.883 26.265 1.00 0.72 C

ATOM 2151 O GLU B 136 8.533 39.326 26.276 1.00 0.72 O

ATOM 2152 CB GLU B 136 5.509 40.325 25.332 1.00 0.72 C

ATOM 2153 CG GLU B 136 6.214 41.636 25.709 1.00 0.72 C

ATOM 2154 CD GLU B 136 6.818 41.611 27.124 1.00 0.72 C

ATOM 2155 OE1 GLU B 136 6.240 40.915 27.991 1.00 0.72 O

ATOM 2156 OE2 GLU B 136 7.852 42.287 27.307 1.00 0.72 O1-

ATOM 2157 N SER B 137 6.866 38.166 27.251 1.00 0.78 N

ATOM 2158 CA SER B 137 7.640 37.791 28.448 1.00 0.78 C

ATOM 2159 C SER B 137 8.828 36.908 28.062 1.00 0.78 C

ATOM 2160 O SER B 137 9.970 37.196 28.412 1.00 0.78 O

ATOM 2161 CB SER B 137 6.768 37.020 29.438 1.00 0.78 C

ATOM 2162 OG SER B 137 5.672 37.840 29.837 1.00 0.78 O

ATOM 2163 N ILE B 138 8.539 35.896 27.248 1.00 0.71 N

ATOM 2164 CA ILE B 138 9.585 35.017 26.697 1.00 0.71 C

ATOM 2165 C ILE B 138 10.487 35.756 25.696 1.00 0.71 C

ATOM 2166 O ILE B 138 11.603 35.342 25.397 1.00 0.71 O

ATOM 2167 CB ILE B 138 8.968 33.752 26.071 1.00 0.71 C

ATOM 2168 CG1 ILE B 138 10.045 32.764 25.614 1.00 0.71 C

ATOM 2169 CG2 ILE B 138 8.081 34.051 24.869 1.00 0.71 C

ATOM 2170 CD1 ILE B 138 10.952 32.333 26.770 1.00 0.71 C

ATOM 2171 N ASP B 139 9.942 36.832 25.151 1.00 0.67 N

ATOM 2172 CA ASP B 139 10.615 37.648 24.135 1.00 0.67 C

ATOM 2173 C ASP B 139 11.007 39.000 24.747 1.00 0.67 C

ATOM 2174 O ASP B 139 10.998 40.063 24.135 1.00 0.67 O

ATOM 2175 CB ASP B 139 9.698 37.826 22.921 1.00 0.67 C

ATOM 2176 CG ASP B 139 10.324 38.528 21.718 1.00 0.67 C

ATOM 2177 OD1 ASP B 139 11.173 39.420 21.866 1.00 0.67 O

ATOM 2178 OD2 ASP B 139 9.887 38.248 20.591 1.00 0.67 O1-

ATOM 2179 N LYS B 140 11.310 38.985 26.028 1.00 0.59 N

ATOM 2180 CA LYS B 140 11.742 40.213 26.708 1.00 0.59 C

ATOM 2181 C LYS B 140 13.105 40.621 26.142 1.00 0.59 C

ATOM 2182 O LYS B 140 14.006 39.794 25.974 1.00 0.59 O

ATOM 2183 CB LYS B 140 11.818 39.967 28.214 1.00 0.59 C

ATOM 2184 CG LYS B 140 12.821 38.865 28.568 1.00 0.59 C

ATOM 2185 CD LYS B 140 12.867 38.628 30.071 1.00 0.59 C

ATOM 2186 CE LYS B 140 13.874 37.536 30.435 1.00 0.59 C

ATOM 2187 NZ LYS B 140 15.250 37.945 30.128 1.00 0.59 N1+

ATOM 2188 N VAL B 141 13.237 41.904 25.841 1.00 0.54 N

ATOM 2189 CA VAL B 141 14.511 42.457 25.344 1.00 0.54 C

ATOM 2190 C VAL B 141 15.593 42.280 26.420 1.00 0.54 C

ATOM 2191 O VAL B 141 15.339 42.473 27.608 1.00 0.54 O

ATOM 2192 CB VAL B 141 14.333 43.937 24.976 1.00 0.54 C

ATOM 2193 CG1 VAL B 141 13.922 44.794 26.183 1.00 0.54 C

ATOM 2194 CG2 VAL B 141 15.599 44.491 24.312 1.00 0.54 C

ATOM 2195 N GLY B 142 16.806 41.980 25.948 1.00 0.56 N

ATOM 2196 CA GLY B 142 17.923 41.668 26.863 1.00 0.56 C

ATOM 2197 C GLY B 142 17.804 40.232 27.397 1.00 0.56 C

ATOM 2198 O GLY B 142 18.761 39.682 27.929 1.00 0.56 O

ATOM 2199 N GLY B 143 16.619 39.648 27.186 1.00 0.61 N

ATOM 2200 CA GLY B 143 16.342 38.250 27.528 1.00 0.61 C

ATOM 2201 C GLY B 143 16.776 37.391 26.367 1.00 0.61 C

ATOM 2202 O GLY B 143 17.571 36.467 26.495 1.00 0.61 O

ATOM 2203 N LEU B 144 16.130 37.701 25.264 1.00 0.57 N

ATOM 2204 CA LEU B 144 16.759 37.482 23.985 1.00 0.57 C

ATOM 2205 C LEU B 144 16.715 38.757 23.173 1.00 0.57 C

ATOM 2206 O LEU B 144 15.788 39.551 23.304 1.00 0.57 O

ATOM 2207 CB LEU B 144 16.089 36.338 23.248 1.00 0.57 C

ATOM 2208 CG LEU B 144 16.798 36.044 21.951 1.00 0.57 C

ATOM 2209 CD1 LEU B 144 18.076 35.506 22.547 1.00 0.57 C

ATOM 2210 CD2 LEU B 144 16.319 34.843 21.168 1.00 0.57 C

ATOM 2211 N SER B 145 17.834 39.019 22.512 1.00 0.62 N

ATOM 2212 CA SER B 145 17.913 40.180 21.617 1.00 0.62 C

ATOM 2213 C SER B 145 16.936 39.946 20.461 1.00 0.62 C

ATOM 2214 O SER B 145 16.631 38.806 20.098 1.00 0.62 O

ATOM 2215 CB SER B 145 19.340 40.339 21.089 1.00 0.62 C

ATOM 2216 OG SER B 145 19.676 39.219 20.263 1.00 0.62 O

ATOM 2217 N VAL B 146 16.471 41.041 19.875 1.00 0.67 N

ATOM 2218 CA VAL B 146 15.633 40.952 18.665 1.00 0.67 C

ATOM 2219 C VAL B 146 16.395 40.212 17.557 1.00 0.67 C

ATOM 2220 O VAL B 146 15.795 39.488 16.779 1.00 0.67 O

ATOM 2221 CB VAL B 146 15.205 42.350 18.193 1.00 0.67 C

ATOM 2222 CG1 VAL B 146 16.404 43.229 17.805 1.00 0.67 C

ATOM 2223 CG2 VAL B 146 14.200 42.251 17.039 1.00 0.67 C

ATOM 2224 N ASP B 147 17.718 40.360 17.547 1.00 0.70 N

ATOM 2225 CA ASP B 147 18.575 39.701 16.554 1.00 0.70 C

ATOM 2226 C ASP B 147 18.469 38.179 16.677 1.00 0.70 C

ATOM 2227 O ASP B 147 18.374 37.434 15.710 1.00 0.70 O

ATOM 2228 CB ASP B 147 20.027 40.151 16.714 1.00 0.70 C

ATOM 2229 CG ASP B 147 20.923 39.498 15.656 1.00 0.70 C

ATOM 2230 OD1 ASP B 147 20.755 39.860 14.474 1.00 0.70 O

ATOM 2231 OD2 ASP B 147 21.746 38.656 16.072 1.00 0.70 O1-

ATOM 2232 N ASP B 148 18.509 37.696 17.897 1.00 0.72 N

ATOM 2233 CA ASP B 148 18.406 36.248 18.065 1.00 0.72 C

ATOM 2234 C ASP B 148 17.011 35.715 17.716 1.00 0.72 C

ATOM 2235 O ASP B 148 16.902 34.676 17.069 1.00 0.72 O

ATOM 2236 CB ASP B 148 18.864 35.902 19.457 1.00 0.72 C

ATOM 2237 CG ASP B 148 18.831 34.381 19.742 1.00 0.72 C

ATOM 2238 OD1 ASP B 148 18.015 33.659 19.136 1.00 0.72 O

ATOM 2239 OD2 ASP B 148 19.491 33.967 20.714 1.00 0.72 O1-

ATOM 2240 N PHE B 149 15.983 36.489 18.042 1.00 0.68 N

ATOM 2241 CA PHE B 149 14.603 36.110 17.696 1.00 0.68 C

ATOM 2242 C PHE B 149 14.363 36.118 16.192 1.00 0.68 C

ATOM 2243 O PHE B 149 13.681 35.250 15.650 1.00 0.68 O

ATOM 2244 CB PHE B 149 13.591 37.045 18.365 1.00 0.68 C

ATOM 2245 CG PHE B 149 13.492 36.889 19.885 1.00 0.68 C

ATOM 2246 CD1 PHE B 149 13.629 35.662 20.469 1.00 0.68 C

ATOM 2247 CD2 PHE B 149 13.243 37.949 20.713 1.00 0.68 C

ATOM 2248 CE1 PHE B 149 13.525 35.408 21.837 1.00 0.68 C

ATOM 2249 CE2 PHE B 149 13.171 37.734 22.088 1.00 0.68 C

ATOM 2250 CZ PHE B 149 13.278 36.485 22.657 1.00 0.68 C

ATOM 2251 N ILE B 150 14.992 37.079 15.528 1.00 0.73 N

ATOM 2252 CA ILE B 150 14.859 37.219 14.069 1.00 0.73 C

ATOM 2253 C ILE B 150 15.545 36.036 13.375 1.00 0.73 C

ATOM 2254 O ILE B 150 15.014 35.478 12.417 1.00 0.73 O

ATOM 2255 CB ILE B 150 15.422 38.553 13.549 1.00 0.73 C

ATOM 2256 CG1 ILE B 150 16.932 38.647 13.743 1.00 0.73 C

ATOM 2257 CG2 ILE B 150 14.671 39.740 14.162 1.00 0.73 C

ATOM 2258 CD1 ILE B 150 17.644 39.889 13.223 1.00 0.73 C

ATOM 2259 N ALA B 151 16.685 35.632 13.939 1.00 0.80 N

ATOM 2260 CA ALA B 151 17.447 34.476 13.448 1.00 0.80 C

ATOM 2261 C ALA B 151 16.630 33.197 13.619 1.00 0.80 C

ATOM 2262 O ALA B 151 16.552 32.376 12.707 1.00 0.80 O

ATOM 2263 CB ALA B 151 18.751 34.354 14.231 1.00 0.80 C

ATOM 2264 N MET B 152 15.914 33.125 14.737 1.00 0.76 N

ATOM 2265 CA MET B 152 15.053 31.973 14.996 1.00 0.76 C

ATOM 2266 C MET B 152 13.941 31.878 13.952 1.00 0.76 C

ATOM 2267 O MET B 152 13.707 30.808 13.402 1.00 0.76 O

ATOM 2268 CB MET B 152 14.415 32.118 16.349 1.00 0.76 C

ATOM 2269 CG MET B 152 13.484 30.924 16.551 1.00 0.76 C

ATOM 2270 SD MET B 152 14.366 29.385 16.991 1.00 0.76 S

ATOM 2271 CE MET B 152 13.017 28.246 17.219 1.00 0.76 C

ATOM 2272 N ASN B 153 13.322 33.016 13.664 1.00 0.76 N

ATOM 2273 CA ASN B 153 12.229 33.052 12.689 1.00 0.76 C

ATOM 2274 C ASN B 153 12.736 32.694 11.289 1.00 0.76 C

ATOM 2275 O ASN B 153 12.139 31.870 10.601 1.00 0.76 O

ATOM 2276 CB ASN B 153 11.615 34.441 12.634 1.00 0.76 C

ATOM 2277 CG ASN B 153 10.392 34.461 11.702 1.00 0.76 C

ATOM 2278 ND2 ASN B 153 9.511 35.396 11.886 1.00 0.76 N

ATOM 2279 OD1 ASN B 153 10.217 33.670 10.801 1.00 0.76 O

ATOM 2280 N LYS B 154 13.858 33.303 10.913 1.00 0.75 N

ATOM 2281 CA LYS B 154 14.461 33.107 9.586 1.00 0.75 C

ATOM 2282 C LYS B 154 14.801 31.629 9.376 1.00 0.75 C

ATOM 2283 O LYS B 154 14.411 31.035 8.381 1.00 0.75 O

ATOM 2284 CB LYS B 154 15.729 33.954 9.487 1.00 0.75 C

ATOM 2285 CG LYS B 154 16.376 33.886 8.101 1.00 0.75 C

ATOM 2286 CD LYS B 154 17.715 34.619 8.113 1.00 0.75 C

ATOM 2287 CE LYS B 154 18.428 34.516 6.763 1.00 0.75 C

ATOM 2288 NZ LYS B 154 19.789 35.069 6.852 1.00 0.75 N1+

ATOM 2289 N LEU B 155 15.411 31.025 10.382 1.00 0.78 N

ATOM 2290 CA LEU B 155 15.834 29.628 10.291 1.00 0.78 C

ATOM 2291 C LEU B 155 14.629 28.681 10.355 1.00 0.78 C

ATOM 2292 O LEU B 155 14.581 27.689 9.629 1.00 0.78 O

ATOM 2293 CB LEU B 155 16.859 29.372 11.391 1.00 0.78 C

ATOM 2294 CG LEU B 155 17.450 27.986 11.196 1.00 0.78 C

ATOM 2295 CD1 LEU B 155 18.706 27.850 11.981 1.00 0.78 C

ATOM 2296 CD2 LEU B 155 16.579 26.924 11.789 1.00 0.78 C

ATOM 2297 N LEU B 156 13.682 28.999 11.228 1.00 0.79 N

ATOM 2298 CA LEU B 156 12.475 28.180 11.386 1.00 0.79 C

ATOM 2299 C LEU B 156 11.719 28.066 10.055 1.00 0.79 C

ATOM 2300 O LEU B 156 11.356 26.968 9.635 1.00 0.79 O

ATOM 2301 CB LEU B 156 11.628 28.766 12.519 1.00 0.79 C

ATOM 2302 CG LEU B 156 10.417 27.876 12.766 1.00 0.79 C

ATOM 2303 CD1 LEU B 156 9.849 27.782 14.179 1.00 0.79 C

ATOM 2304 CD2 LEU B 156 9.372 28.557 11.941 1.00 0.79 C

ATOM 2305 N GLN B 157 11.505 29.213 9.416 1.00 0.77 N

ATOM 2306 CA GLN B 157 10.828 29.252 8.109 1.00 0.77 C

ATOM 2307 C GLN B 157 11.640 28.588 6.994 1.00 0.77 C

ATOM 2308 O GLN B 157 11.084 27.873 6.159 1.00 0.77 O

ATOM 2309 CB GLN B 157 10.448 30.683 7.728 1.00 0.77 C

ATOM 2310 CG GLN B 157 11.675 31.575 7.561 1.00 0.77 C

ATOM 2311 CD GLN B 157 11.300 32.983 7.144 1.00 0.77 C

ATOM 2312 NE2 GLN B 157 11.681 33.940 7.963 1.00 0.77 N

ATOM 2313 OE1 GLN B 157 10.707 33.195 6.097 1.00 0.77 O

ATOM 2314 N ARG B 158 12.958 28.780 7.046 1.00 0.74 N

ATOM 2315 CA ARG B 158 13.866 28.205 6.038 1.00 0.74 C

ATOM 2316 C ARG B 158 13.895 26.687 6.137 1.00 0.74 C

ATOM 2317 O ARG B 158 13.868 25.980 5.134 1.00 0.74 O

ATOM 2318 CB ARG B 158 15.282 28.746 6.195 1.00 0.74 C

ATOM 2319 CG ARG B 158 15.350 30.219 5.797 1.00 0.74 C

ATOM 2320 CD ARG B 158 16.754 30.780 5.984 1.00 0.74 C

ATOM 2321 NE ARG B 158 17.676 30.135 5.037 1.00 0.74 N

ATOM 2322 CZ ARG B 158 18.995 30.330 5.004 1.00 0.74 C

ATOM 2323 NH1 ARG B 158 19.577 31.155 5.869 1.00 0.74 N1+

ATOM 2324 NH2 ARG B 158 19.742 29.702 4.104 1.00 0.74 N

ATOM 2325 N LEU B 159 13.839 26.219 7.370 1.00 0.78 N

ATOM 2326 CA LEU B 159 13.663 24.811 7.620 1.00 0.78 C

ATOM 2327 C LEU B 159 12.472 24.087 7.119 1.00 0.78 C

ATOM 2328 O LEU B 159 12.562 23.017 6.520 1.00 0.78 O

ATOM 2329 CB LEU B 159 13.768 24.526 9.102 1.00 0.78 C

ATOM 2330 CG LEU B 159 15.188 24.435 9.478 1.00 0.78 C

ATOM 2331 CD1 LEU B 159 15.056 24.293 11.035 1.00 0.78 C

ATOM 2332 CD2 LEU B 159 15.773 23.481 8.348 1.00 0.78 C

ATOM 2333 N ASN B 160 11.396 24.798 7.350 1.00 0.77 N

ATOM 2334 CA ASN B 160 10.101 24.296 6.914 1.00 0.77 C

ATOM 2335 C ASN B 160 10.099 24.150 5.394 1.00 0.77 C

ATOM 2336 O ASN B 160 9.707 23.121 4.852 1.00 0.77 O

ATOM 2337 CB ASN B 160 9.061 25.317 7.286 1.00 0.77 C

ATOM 2338 CG ASN B 160 7.663 24.828 6.954 1.00 0.77 C

ATOM 2339 ND2 ASN B 160 6.842 25.785 6.617 1.00 0.77 N

ATOM 2340 OD1 ASN B 160 7.349 23.648 6.919 1.00 0.77 O

ATOM 2341 N ARG B 161 10.677 25.168 4.767 1.00 0.71 N

ATOM 2342 CA ARG B 161 10.835 25.252 3.312 1.00 0.71 C

ATOM 2343 C ARG B 161 11.761 24.134 2.809 1.00 0.71 C

ATOM 2344 O ARG B 161 11.394 23.355 1.930 1.00 0.71 O

ATOM 2345 CB ARG B 161 11.440 26.629 3.075 1.00 0.71 C

ATOM 2346 CG ARG B 161 11.116 27.215 1.705 1.00 0.71 C

ATOM 2347 CD ARG B 161 10.827 28.714 1.833 1.00 0.71 C

ATOM 2348 NE ARG B 161 11.919 29.422 2.529 1.00 0.71 N

ATOM 2349 CZ ARG B 161 11.765 30.371 3.458 1.00 0.71 C

ATOM 2350 NH1 ARG B 161 10.557 30.772 3.843 1.00 0.71 N1+

ATOM 2351 NH2 ARG B 161 12.832 30.962 3.981 1.00 0.71 N

ATOM 2352 N PHE B 162 12.877 23.965 3.517 1.00 0.71 N

ATOM 2353 CA PHE B 162 13.920 22.984 3.179 1.00 0.71 C

ATOM 2354 C PHE B 162 13.410 21.540 3.226 1.00 0.71 C

ATOM 2355 O PHE B 162 13.588 20.786 2.269 1.00 0.71 O

ATOM 2356 CB PHE B 162 15.105 23.172 4.129 1.00 0.71 C

ATOM 2357 CG PHE B 162 16.205 22.136 3.892 1.00 0.71 C

ATOM 2358 CD1 PHE B 162 17.099 22.270 2.845 1.00 0.71 C

ATOM 2359 CD2 PHE B 162 16.349 21.086 4.776 1.00 0.71 C

ATOM 2360 CE1 PHE B 162 18.131 21.353 2.697 1.00 0.71 C

ATOM 2361 CE2 PHE B 162 17.362 20.150 4.632 1.00 0.71 C

ATOM 2362 CZ PHE B 162 18.258 20.289 3.583 1.00 0.71 C

ATOM 2363 N TRP B 163 12.782 21.174 4.338 1.00 0.59 N

ATOM 2364 CA TRP B 163 12.218 19.823 4.482 1.00 0.59 C

ATOM 2365 C TRP B 163 11.095 19.573 3.485 1.00 0.59 C

ATOM 2366 O TRP B 163 10.980 18.472 2.967 1.00 0.59 O

ATOM 2367 CB TRP B 163 11.729 19.601 5.909 1.00 0.59 C

ATOM 2368 CG TRP B 163 12.850 19.600 6.920 1.00 0.59 C

ATOM 2369 CD1 TRP B 163 14.136 19.674 6.649 1.00 0.59 C

ATOM 2370 CD2 TRP B 163 12.712 19.506 8.274 1.00 0.59 C

ATOM 2371 CE2 TRP B 163 13.984 19.510 8.749 1.00 0.59 C

ATOM 2372 CE3 TRP B 163 11.652 19.510 9.150 1.00 0.59 C

ATOM 2373 NE1 TRP B 163 14.852 19.647 7.761 1.00 0.59 N

ATOM 2374 CZ2 TRP B 163 14.115 19.498 10.092 1.00 0.59 C

ATOM 2375 CZ3 TRP B 163 11.841 19.528 10.528 1.00 0.59 C

ATOM 2376 CH2 TRP B 163 13.121 19.528 11.018 1.00 0.59 C

ATOM 2377 N GLY B 164 10.354 20.643 3.168 1.00 0.78 N

ATOM 2378 CA GLY B 164 9.217 20.571 2.236 1.00 0.78 C

ATOM 2379 C GLY B 164 9.738 20.212 0.843 1.00 0.78 C

ATOM 2380 O GLY B 164 9.217 19.401 0.092 1.00 0.78 O

ATOM 2381 N ASP B 165 10.857 20.811 0.494 1.00 0.65 N

ATOM 2382 CA ASP B 165 11.483 20.549 -0.808 1.00 0.65 C

ATOM 2383 C ASP B 165 12.065 19.145 -0.894 1.00 0.65 C

ATOM 2384 O ASP B 165 11.940 18.483 -1.916 1.00 0.65 O

ATOM 2385 CB ASP B 165 12.568 21.581 -1.097 1.00 0.65 C

ATOM 2386 CG ASP B 165 11.982 22.983 -1.283 1.00 0.65 C

ATOM 2387 OD1 ASP B 165 10.772 23.082 -1.587 1.00 0.65 O

ATOM 2388 OD2 ASP B 165 12.785 23.934 -1.179 1.00 0.65 O1-

ATOM 2389 N GLN B 166 12.639 18.694 0.212 1.00 0.61 N

ATOM 2390 CA GLN B 166 13.216 17.350 0.247 1.00 0.61 C

ATOM 2391 C GLN B 166 12.133 16.274 0.131 1.00 0.61 C

ATOM 2392 O GLN B 166 12.309 15.277 -0.556 1.00 0.61 O

ATOM 2393 CB GLN B 166 14.000 17.190 1.531 1.00 0.61 C

ATOM 2394 CG GLN B 166 14.634 15.798 1.569 1.00 0.61 C

ATOM 2395 CD GLN B 166 15.742 15.587 0.595 1.00 0.61 C

ATOM 2396 NE2 GLN B 166 16.904 16.072 0.971 1.00 0.61 N

ATOM 2397 OE1 GLN B 166 15.528 14.956 -0.421 1.00 0.61 O

ATOM 2398 N ILE B 167 10.992 16.542 0.750 1.00 0.54 N

ATOM 2399 CA ILE B 167 9.848 15.615 0.704 1.00 0.54 C

ATOM 2400 C ILE B 167 9.277 15.498 -0.712 1.00 0.54 C

ATOM 2401 O ILE B 167 8.812 14.436 -1.111 1.00 0.54 O

ATOM 2402 CB ILE B 167 8.734 16.003 1.683 1.00 0.54 C

ATOM 2403 CG1 ILE B 167 8.064 17.320 1.343 1.00 0.54 C

ATOM 2404 CG2 ILE B 167 9.332 16.216 3.046 1.00 0.54 C

ATOM 2405 CD1 ILE B 167 6.951 17.848 2.248 1.00 0.54 C

ATOM 2406 N ALA B 168 9.270 16.630 -1.414 1.00 0.56 N

ATOM 2407 CA ALA B 168 8.738 16.708 -2.781 1.00 0.56 C

ATOM 2408 C ALA B 168 9.742 16.229 -3.836 1.00 0.56 C

ATOM 2409 O ALA B 168 9.349 15.804 -4.920 1.00 0.56 O

ATOM 2410 CB ALA B 168 8.317 18.148 -3.079 1.00 0.56 C

ATOM 2411 N TYR B 169 11.027 16.299 -3.502 1.00 0.35 N

ATOM 2412 CA TYR B 169 12.106 16.097 -4.484 1.00 0.35 C

ATOM 2413 C TYR B 169 13.044 14.932 -4.132 1.00 0.35 C

ATOM 2414 O TYR B 169 13.713 14.447 -5.069 1.00 0.35 O

ATOM 2415 CB TYR B 169 12.882 17.408 -4.632 1.00 0.35 C

ATOM 2416 CG TYR B 169 13.994 17.299 -5.675 1.00 0.35 C

ATOM 2417 CD1 TYR B 169 13.688 17.439 -7.021 1.00 0.35 C

ATOM 2418 CD2 TYR B 169 15.307 17.093 -5.269 1.00 0.35 C

ATOM 2419 CE1 TYR B 169 14.703 17.388 -7.967 1.00 0.35 C

ATOM 2420 CE2 TYR B 169 16.321 17.043 -6.215 1.00 0.35 C

ATOM 2421 CZ TYR B 169 16.017 17.188 -7.563 1.00 0.35 C

ATOM 2422 OH TYR B 169 17.004 17.123 -8.492 1.00 0.35 O

ATOM 2423 OXT TYR B 169 13.141 14.615 -2.932 1.00 0.35 O1-

END
